# Positional cues and cell division dynamics drive meristem development and archegonium formation in Ceratopteris gametophytes

**DOI:** 10.1038/s42003-022-03627-y

**Published:** 2022-07-01

**Authors:** Yuan Geng, An Yan, Yun Zhou

**Affiliations:** 1 Department of Botany and Plant Pathology, Purdue University, West Lafayette, IN 47907 USA; 2 Purdue Center for Plant Biology, Purdue University, West Lafayette, IN 47907 USA; 3 Division of Biology and Biological Engineering, California Institute of Technology, Pasadena, CA 91125 USA; 4 Howard Hughes Medical Institute, California Institute of Technology, Pasadena, CA 91125 USA

**Keywords:** Plant stem cell, Plant cell cycle, Plant regeneration

## Abstract

Fern gametophytes are autotrophic and independent of sporophytes, and they develop pluripotent meristems that drive prothallus development and sexual reproduction. To reveal cellular dynamics during meristem development in fern gametophytes, we performed long-term time-lapse imaging and determined the real-time lineage, identity and division activity of each single cell from meristem initiation to establishment in gametophytes of the fern *Ceratopteris richardii*. Our results demonstrate that in Ceratopteris gametophytes, only a few cell lineages originated from the marginal layer contribute to meristem initiation and proliferation, and the meristem lacks a distinguishable central zone or apical cell with low division activity. Within the meristem, cell division is independent of cell lineages and cells at the marginal layer are more actively dividing than inner cells. Furthermore, the meristem triggers differentiation of adjacent cells into egg-producing archegonia in a position-dependent manner. These findings advance the understanding of diversified meristem and gametophyte development in land plants.

## Abstract (web summary)

Time-lapse imaging of the fern Ceratopteris richardii during meristem initiation and proliferation provides insights into the lineage, identity and division activity of each cell throughout the growth of gametophytes.

## Introduction

The lifecycle of land plants alternates between two generations: the asexual sporophyte and the sexual gametophyte^1,2^. In seed plants, the sporophytes, representing the dominant generation, develop pluripotent apical meristems (including shoot apical meristems and root apical meristems), which sustain the growth and development of plant body during their life span^3,4^. The gametophytes of seed plants are greatly reduced in size, devoid of a meristem, and dependent on their sporophytes^1,5,6^. By contrast, in seed-free vascular plants, including ferns, the gametophyte and sporophyte are mutually independent generations^7–9^. Fern gametophytes develop meristems that renew themselves through continuous cell division and produce new cells that differentiate into a photosynthetic prothallus or into cells that form the gametangia (egg-bearing archegonia and sperm-bearing antheridia)^10–12^. The timing of meristem initiation and maintenance plays a key role in shaping gametophyte morphology^10,13^. Compared to the well-characterized cell behaviors and regulatory circuits identified in the meristems of sporophytes in seed plants, especially in Arabidopsis^3,14–18^, the mechanisms underlying meristem development in fern gametophytes are just beginning to be understood in only a few fern species^9–11,13,19–25^.

The homosporous fern *Ceratopteris richardii* (hereafter ‘Ceratopteris’) has been developed and widely used as a model system for studying many aspects of evolutionary and developmental questions in ferns^11,20–22,26–40^. Like many other homosporous ferns, the sex of Ceratopteris gametophytes is determined by a pheromone called antheridiogen^11,37,41^. A spore germinates and then develops into a hermaphroditic or male gametophyte, depending on the absence or presence of antheridiogen^11,37^. Male gametophytes are ameristic, differentiating multiple antheridia that produce sperm^11,19,22,39^. The hermaphroditic gametophyte develops one multicellular meristem, which was also called the lateral meristem, marginal meristem, or notch meristem^11,13,19,22,39^. Once the multicellular meristem is established, the egg-bearing organ archegonia initiate next to the meristem notch until fertilization^11,19,22^. To date, the dynamic cell behaviors responsible for meristem initiation and maintenance in Ceratopteris gametophytes, and the cellular mechanism by which the meristem promotes organogenesis (e.g., archegonium formation) have yet to be identified. Cell lineages and spatiotemporal patterns of cell divisions during gametophyte development are also completely lacking. For these reasons, we have generated Ceratopteris stable transgenic plants that allow the labeling of each individual cell (nucleus) and performed long-term time-lapse confocal imaging during meristem initiation and proliferation in haploid gametophytes. We then established a computational pipeline to quantitatively determine the lineage, identity, and division activity of each cell throughout the growth of gametophytes. Through mechanical perturbations, we also revealed cell fate re-specification and cell-cell communications during the *de novo* formation of meristems and archegonia. Our work reveals the cellular basis of a multicellular meristem in gametophytes and help in understanding the diversified meristem development and organ formation in land plants.

## Results

### Stably transformed Ceratopteris lines expressing a fluorescent nuclear marker

**Fig. 1:**
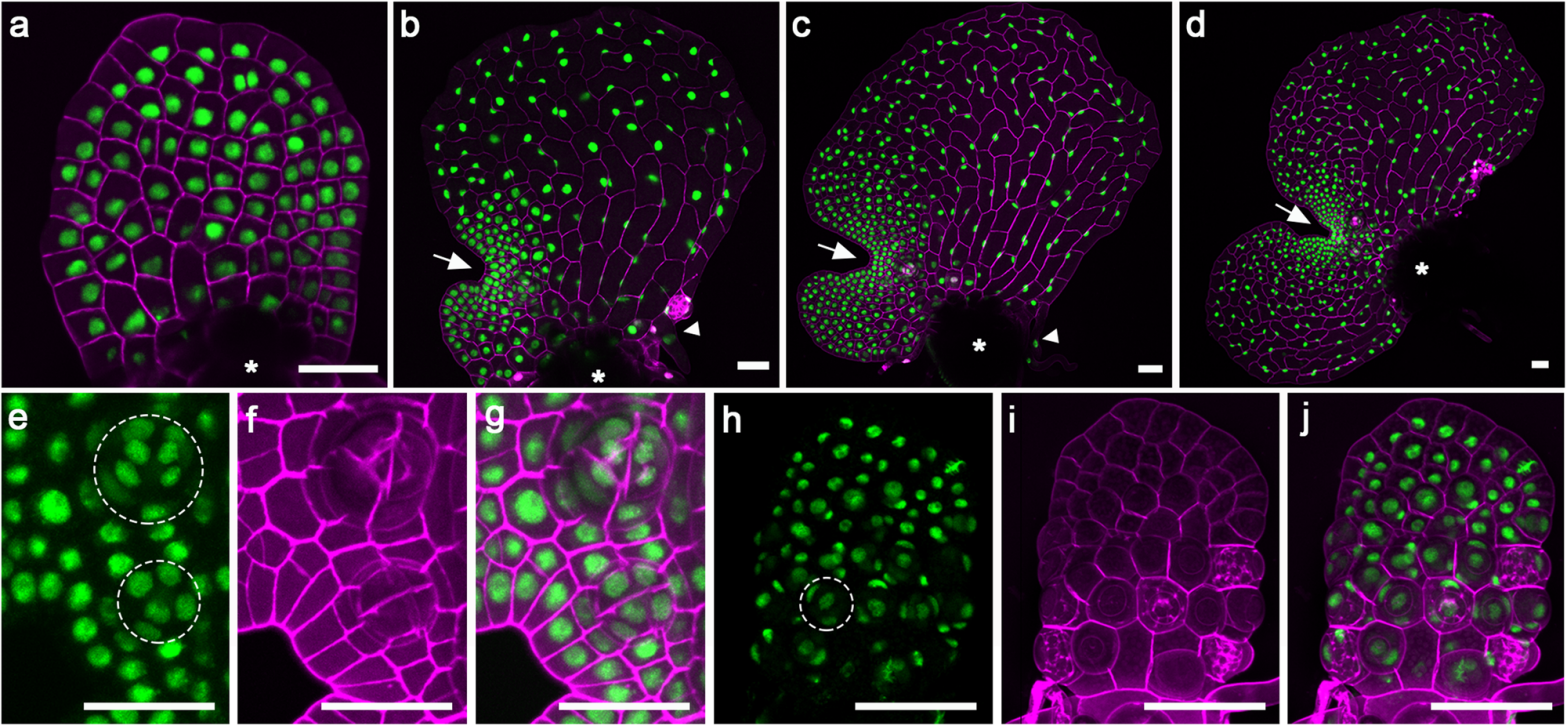
Confocal imaging of Ceratopteris gametophytes expressing a fluorescent nuclear marker at different developmental stages. **a**–**j** Ceratopteris gametophytes expressing the nuclear marker Histone 2B (H2B)-GFP were stained and imaged using laser scanning confocal microscopy. **a**–**d** Z-projection views of different hermaphroditic gametophytes at 7 days after inoculation, DAI (**a**), 9 DAI (**b**), 11 DAI (**c**), and 12 DAI (**d**). The white arrow indicates the meristem notch of each gametophyte. Asterisks indicate areas where spore coats are located. White arrowheads indicate rhizoids. **e**–**g** A close-up view of the meristem and adjacent archegonia in one hermaphroditic gametophyte at 13 DAI. Each archegonium is composed of multiple cells (nuclei) and highlighted with the dashed circle. **h**–**j** The z-projection view of a male gametophyte at 8 DAI. One antheridium is highlighted with the dashed circle. **a**–**d**, **g**, **j** Merge of GFP (green) and propidium iodide (PI, purple) channels. **e**, **h** GFP channel (green). **f**, **i** PI channel (purple). The transgenic line 24 was used for the confocal imaging in this figure. Scale bars: 50 μm.

To determine cellular dynamics and cell cycle progression during meristem initiation and proliferation in gametophytes, we generated a fluorescent reporter that marks each nucleus in Ceratopteris gametophytes except for gametes (Fig. 1; Supplementary Fig. 1). Specifically, we stably transformed and identified Ceratopteris transgenic plants with a Histone 2B-GFP (H2B-GFP) reporter^42^ under the control of endogenous 5’ promoter and 3’ terminator of the Ceratopteris *HAIRY MERISTEM* (*CrHAM*) gene^43,44^ (See Methods for details). Through laser scanning confocal imaging, we found that this H2B-GFP reporter was uniformly expressed in the nuclei of both male and hermaphroditic prothalli in the transgenic lines, throughout their developmental stages (Fig. 1; Supplementary Fig. 1; Supplementary Movies 1 and 2). Starting from 6 days after inoculation (DAI), the meristem in hermaphroditic gametophytes actively and continuously proliferated, resulting in a notch at one side of the prothallus (Fig. 1a–d). The notch separated two wings of the prothallus, with one developed first, evident in Fig. 1b and c. At these stages, the H2B-GFP reporter was highly expressed in every single nucleus of the multicellular meristems (Fig. 1a–d). The H2B-GFP reporter also clearly labeled all the nuclei from the differentiated cells that compose archegonia (Fig. 1e–g; Supplementary Movie 3), antheridia (Fig. 1h–j), and rhizoids (Fig. 1b, c). In addition, the morphology of gametophytes from these transgenic lines was comparable to that of wild-type hermaphroditic gametophytes (Supplementary Fig. 2), suggesting that the transgene did not interfere with normal growth and development of gametophytes. At least three independent transgenic lines showed comparable expression levels and patterns in the Ceratopteris gametophytes, and one line was included in the following experiments (Fig. 1; Supplementary Fig. 3). Together these results demonstrated that the reporter is suitable for studying cell fates and lineages in fern gametophytes.

### Long term time-lapse imaging reveals dynamic cell behaviors in Ceratopteris gametophytes from formation to maintenance of a multicellular meristem

**Fig. 2:**
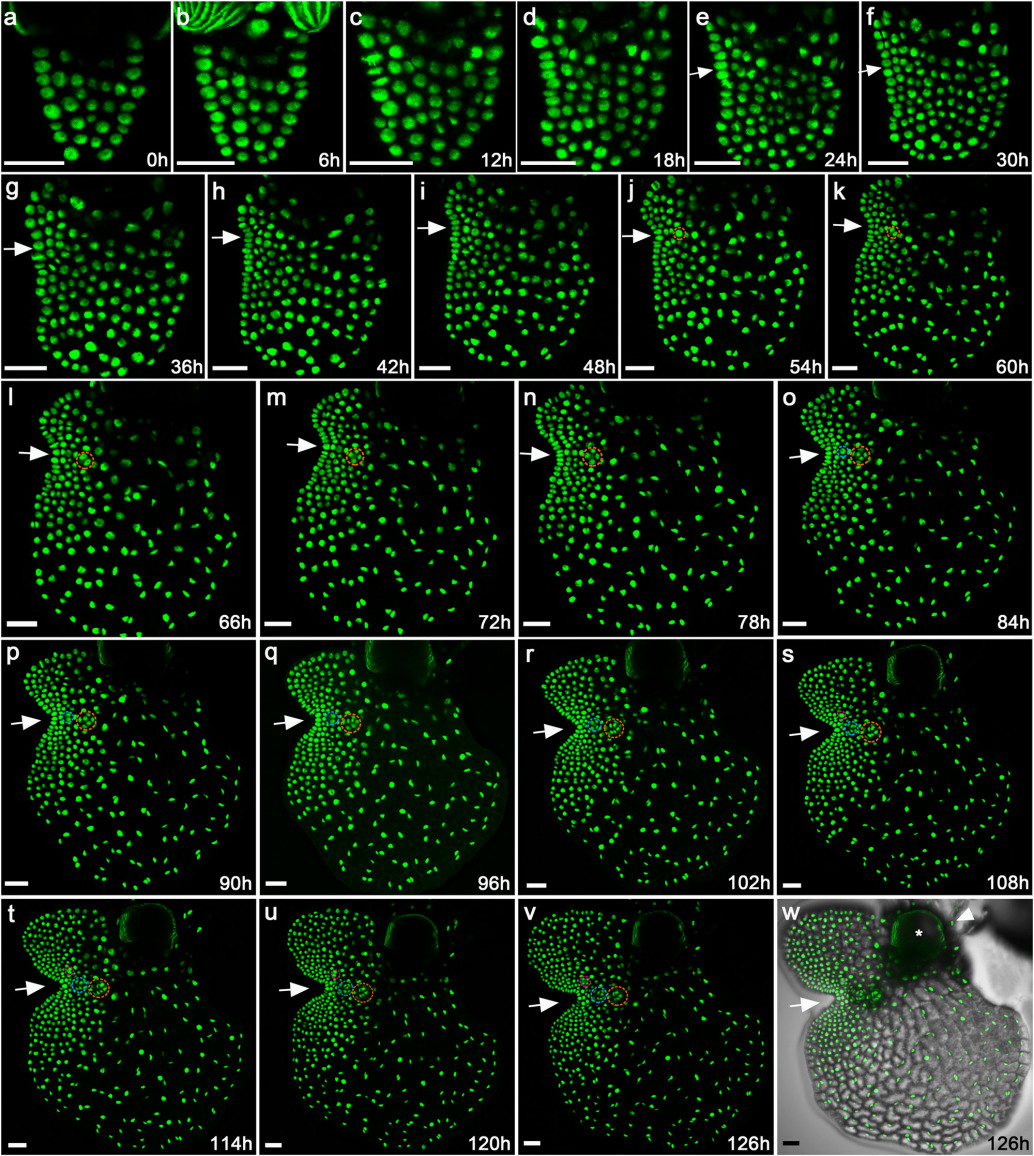
Long-term time-lapse confocal imaging reveals cellular dynamics of meristem initiation and proliferation in the Ceratopteris gametophyte. **a**–**v** One hermaphroditic gametophyte expressing the nuclear marker H2B-GFP (at 5 DAI) was live-imaged through laser scanning confocal microscopy. The non-invasive time-lapse imaging was performed at 6-h intervals, from 0 h to 126 h. **a**–**v** Z-projection views of confocal images at the indicated time points are shown. White arrows indicate formation and proliferation of the multicellular meristem over time. Three archegonia subsequently formed next to the meristem, which are highlighted with the circles in different colors (orange, blue and pink). **w** Merge of the GFP (**v**) and DIC (showing the cell outlines) channels. The white asterisk indicates ruptured spore coat, and the white arrowhead indicates rhizoids. Scale bars: 50 μm. Three independent biological replicates were included in this time-lapse imaging experiment with the same interval and duration, all showing similar patterns of prothallus cell proliferation. Two other samples were shown in Supplementary Figs. 4 and 5.

With the established transgenic reporter line, a non-invasive time-lapse imaging experiment was performed to reveal dynamic cell behaviors during the formation and proliferation of the multicellular meristem. Spores of the H2B-GFP transgenic reporter line were inoculated and germinated on solidified growth medium (FM plates, see Methods for details). At 5 days after inoculation (DAI) that corresponded to an intermediate stage between the G3 and G4h defined by Conway and Di Stilio^19^, hermaphroditic gametophytes were imaged as the first time point (0 h) using laser scanning confocal microscopy (Fig. 2a). The full stack of optical sections from top to bottom of the living gametophytes were taken and the Z-projection of the sections was generated to visualize all GFP-labeled nuclei from each prothallus (Fig. 2a). After the first time point was taken, the FM plates of gametophytes were returned to the growth chamber, cultured under the same conditions, and the same samples were imaged again six hours later as the second time point (6 h) (Fig. 2b). This live-imaging process was continuously repeated until a prothallus fully developed (Fig. 2c–v), with a total of 22 time points acquired for each gametophyte, all at six-hour intervals (Fig. 2a–v). Three independent hermaphroditic gametophytes were live-imaged at the same time with the same interval and duration (Fig. 2; Supplementary Figs. 4 and 5).

### A cell lineage map of the gametophyte and meristem

**Fig. 3:**
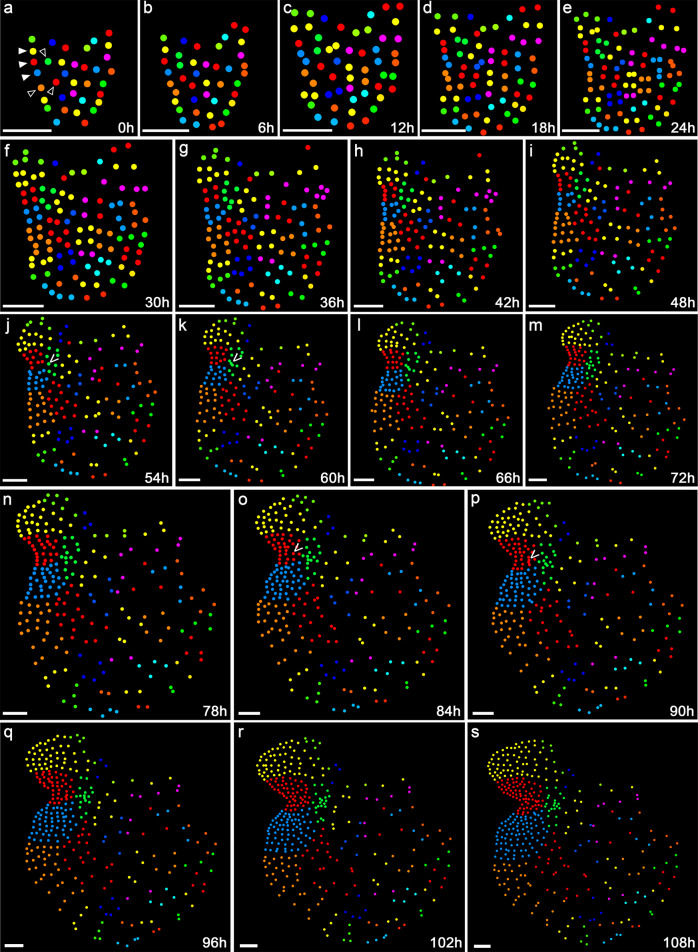
Cell lineage dynamics of the Ceratopteris gametophyte. The nuclei in the confocal images from 0–108 h (Fig. 2) were segmented and each segmented nucleus was labelled with the unique ID as shown in Supplementary Fig. 19. **a**–**s** Each circle represents the location of the segmented individual nucleus shown in Fig. 2. The cells at 0 h are labeled with different colors, as the progenitors for each lineage over the 108 h of cell division and prothallus development. In the subsequent time points, the same color has been used for labeling each progenitor cell and its descendants. The solid arrowheads in (**a**) indicate three marginal cell lineages contributing to the majority of cells of the meristem (named as meristem progenitor cells). The open arrowheads in (**a**) indicate the adjacent lineages in which cells showed relatively lower cell division activity than the three marginal lineages but higher division activity than the other lineages (shown in Supplementary Data 1 and 2). The “V” indicates the progenitor cells of the firstly (**j**, **k**) and secondly (**o**, **p**) initiated archegonia, which are also highlighted with orange circles (in Fig. 2j, k) and blue circles in the confocal images (in Fig. 2o, p), respectively. These two archegonia belong to two different lineages. Scale bars: 50 μm. Three independent samples were analyzed, showing similar patterns of cell lineages during gametophyte development (shown in Fig. 3; Supplementary Figs. 6 and 7).

To determine the fate of each cell of the gametophyte (starting at 5 DAI) as the prothallus developed, we performed two-dimensional (2D) image analysis to segment and detect each nucleus from the images taken at different time points and automatically label each segmented nucleus with a unique ID. Following that, we traced the fate and descendants of each labelled nucleus of all the three gametophytes from the first time point (0 h) to the 19th time point (108 h), when the meristem had been fully established and two archegonia were evident in each gametophyte (Fig. 3a–s; Supplementary Figs. 6a–s, 7a–s). From this data, we constructed lineage maps of all the segmented nuclei over time. Specifically, each nucleus identified at 0 h (Fig. 3a; Supplementary Figs. 6a and 7a) represented a progenitor cell of one lineage. In the following time points, clonally related cells (nuclei) were labeled with the same color throughout the development of the hermaphroditic gametophytes (Fig. 3b–s; Supplementary Figs. 6b–s, 7b–s).

As shown in the color-coded lineage map (Fig. 3a–s), it appears that only a few progenitor cells (three in Fig. 3a, pointed by solid arrowheads) at the marginal/outermost layer of the young gametophyte (at 5 DAI, 0 h in the time-lapse) contributed to the vast majority of meristem cells of the fully developed prothallus (108 h in the time-lapse). Their lineage progression was indicated by the continuously expanding sectors over time (Fig. 3a–s), resulting in the dominant yellow, red, and blue sectors at 108 h (Fig. 3s). In addition, a few adjacent cells (pointed by open arrowheads in Fig. 3a) also divided and contributed to prothallus proliferation (Fig. 3a–s) as their divisions were outpaced by the meristem progenitor cells (pointed by solid arrowheads) and their descendants. In contrast, cells from all the other lineages in each gametophyte did not or rarely divide over the 108h-time frame, representing the meiotically inactive region in the developing gametophyte (Fig. 3a–s). Three independent gametophyte samples were analyzed (Fig. 3a–s; Supplementary Figs. 6a–s, 7a–s), and they all showed similar lineage dynamics during gametophyte development.

### Spatial and temporal dynamics of cell division during meristem formation and proliferation

**Fig. 4:**
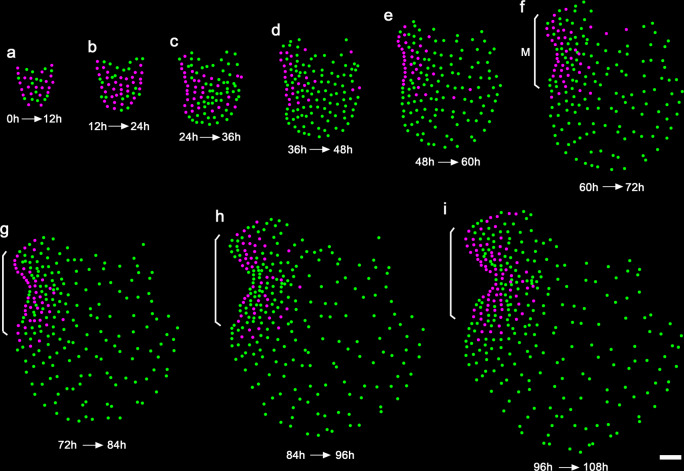
Spatial and temporal dynamics of cell division in the Ceratopteris gametophyte. **a**–**i** Each colored dot represents the detected individual nucleus of the gametophyte shown in Fig. 2. Magenta dots represent cells that divided within the indicated frame; green dots represent cells that did not divide within the indicated time frame. The cell division events of the live-imaged sample are shown in the images from the first time points of the indicated intervals (every 12 h). The brackets indicate the multicellular meristem (M) in the gametophyte. Scale bar: 50 μm. Three independent samples were analyzed, showing similar patterns during gametophyte development.

**Fig. 5:**
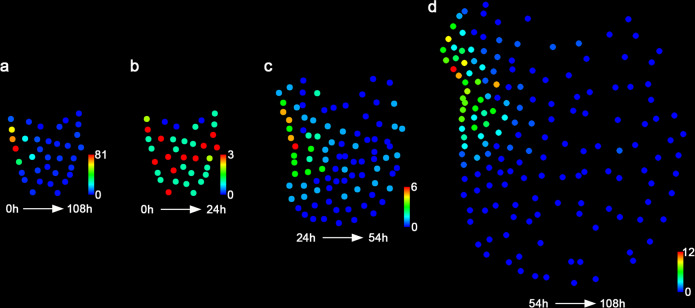
Quantification of cell division events in the Ceratopteris gametophyte. **a**–**d** Quantification of cell division events at different developmental stages, including the time frames of 0–108 h (**a**), 0–24 h (**b**), 24–54 h (**c**), and 54–108 h (**d**). Each colored dot represents the detected individual nuclei of the gametophyte shown in Fig. 2. The quantified cell division events are mapped to the images from the first time points of the indicated intervals. Colors indicate the total number of cell division events for each cell lineage during the indicated time frames, with the scale from blue (0) to red (81) in **a**; from blue (0) to red (3) in **b**; from blue (0) to red (6) in **c**; and from blue (0) to red (12) in **d**. Three independent samples were analyzed, showing similar results (Fig. 5; Supplementary Figs. 8 and 9).

Cell division patterns in the developing hermaphroditic gametophyte were then examined within different time frames over the 108 h (Figs. 4a–i, 5a–d; Supplementary Figs. 8a–d, 9a–d). Three consecutive phases of cell proliferation were defined, including the initiation phase (0–24 h), the transition phase (24–54 h), and the maturation phase (54–108 h) (Fig. 5b–d; Supplementary Figs. 8b–d, 9b–d). During the initiation stage when a meristem was not morphologically distinguishable yet, most progenitor cells showed 1–3 division events (Figs. 4a, b, 5b; Supplementary Data 1 and 2). The active division at the central region and reduced division at the spore end of the gametophyte led to expansion of the prothallus in both lateral and apical directions (Figs. 4a, b and 5b). Starting from the transition phase, division took place more frequently in the cells located on one side of the prothallus, resulting in the formation of the multicellular meristem and its associated notch (Figs. 4c–e and 5c). During the following maturation phase, cell division was restricted to the cells surrounding the meristem notch, whereas most cells outside of the meristem stopped dividing (Figs. 4f–i and 5d). These divisions eventually resulted in the fully established meristem with a distinct meristem notch and two prominent wings. Furthermore, the meristem did not show a distinguishable central zone or apical cell with low cell division activity compared to the surrounding cells (Figs. 4f–i, 5d; Supplementary Figs. 8d and 9d). During the maturation phase, marginal cells (as highlighted with red dashed circles in Supplementary Fig. 10a–c) displayed significantly higher division activity (with the averaged division events of 7.0, *n* = 43 cells from three gametophytes) than those located inside (as highlighted with white dashed circles in Supplementary Fig. 10a–c, with the averaged division events of 4.3, *n* = 32 cells from three gametophytes) in the meristems (Supplementary Fig. 10d).

### Cell division activity within meristems is regulated in a lineage-independent and position-dominated manner

**Fig. 6:**
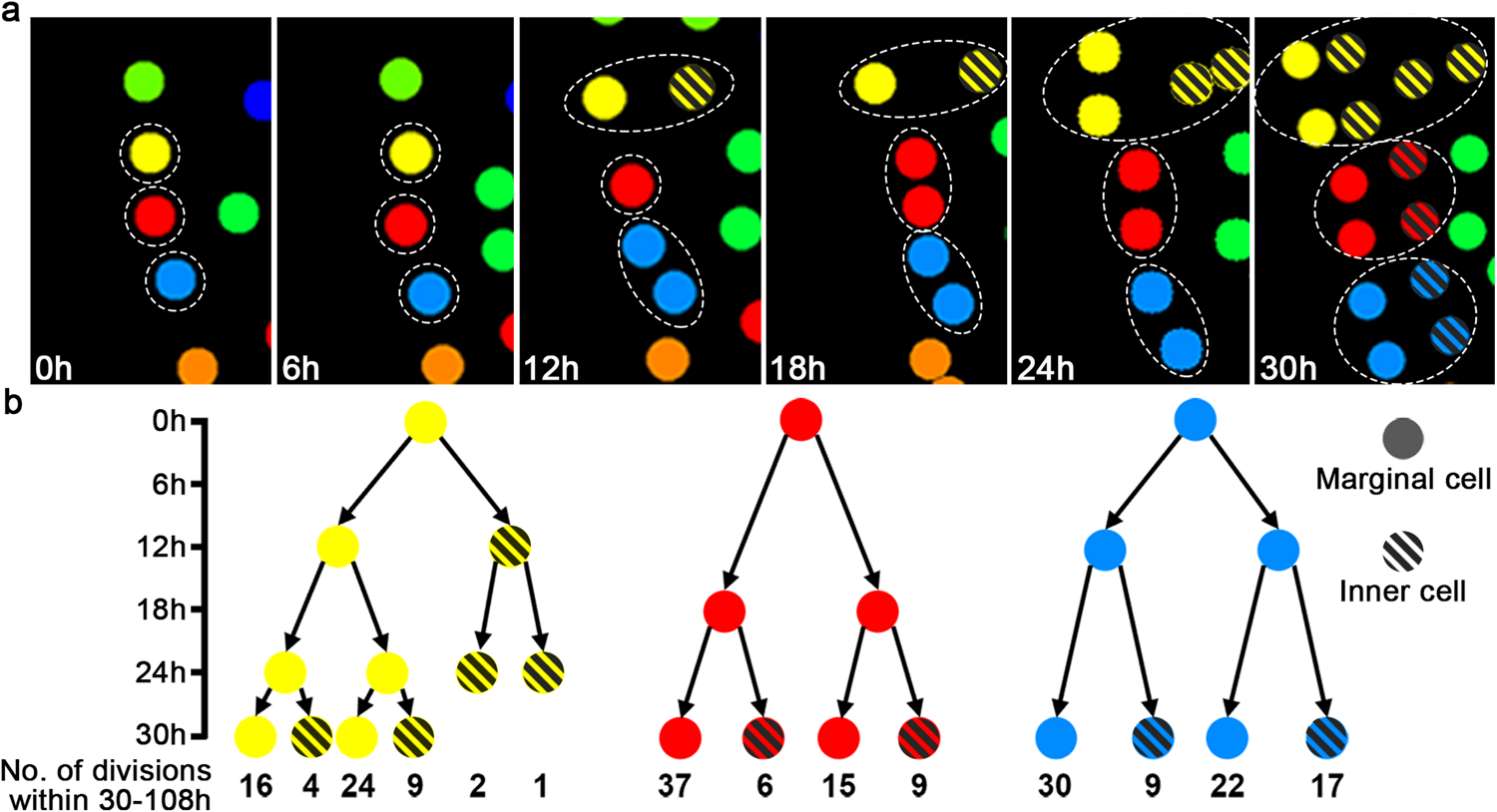
The impact of lineage and position on division activities of meristem progenitor cells. **a** Cell division and lineage progression of three meristem progenitor cells from 0 h to 30 h. The images are part of Fig. 3a–f (0–30 h), highlighting the three cell lineages that contribute to the majority of cells in the meristem with the dashed lines, and labelling marginal cells with solid circles and inner cells with the circles filled with diagonal stripes. The complete lineage progression of the three meristem progenitor cells and their descendants (the yellow, red and blue sectors) is shown in Fig. 3a–s. **b** Relationships among cell lineages, cell position and quantified division events. Each circle of one family tree represents one cell at the indicated time point from 0–30 h. Two black arrows from one cell represent one round of cell division and illustrate the relationship between the cell and its two immediate progenies. *Y*-axis: the time-lapse from 0 h to 30 h. *X*-axis: the total number of cell division events for each cell (at 30 h) and its progenies during the following 30–108 h time frame (the quantified division events with the cell IDs shown in Supplementary Data 3, and cell position with IDs shown in Supplementary Fig. 19). **b** labels the clonally related cells with the same color in each family tree (as yellow, red and blue shown in (**a**) and highlights the positional information of each cell (as solid circles and circles filled with diagonal stripes shown in (**a**). During the 30–108 h time frame, cells from the same progenitor cell display variable division activities, and cells located at the marginal layer (solid circles) of the meristem show more division events than the cells located at the inner (or submarginal) layer (circles filled with diagonal stripes). All the meristem progenitor cells in the three independent gametophyte samples were analyzed (in Fig. 6; Supplementary Figs. 11 and 12), showing similar results.

Our results demonstrate that the meristem is the active division site and localized source of new cells in developing hermaphroditic gametophytes. However, within the meristem, it is not clear whether the cells with high division activity originate from one or a few progenitor cells or whether cell division activities are independent of their origins. To address this question, we further analyzed the three progenitor cells that eventually contributed to the majority of meristem cells (or called meristem progenitor cells) (Figs. 3a–s and 6a). We built the family trees of the clonally related cells from each meristem progenitor cell (yellow, red, and blue, respectively) during the 0–30 h time frame and then quantified the number of cell divisions of each cell (at 30 h) and its progenies during the following 30–108 h time frame (Fig. 6b). We found that cells derived from a common progenitor displayed various division activities within the 30–108 h time frame (Fig. 6b; Supplementary Data 3). Many siblings derived from the same parental cells still showed distinct division activities (Fig. 6b). These results suggest that within the meristem, cell lineages do not directly control division activity of each individual cell. Furthermore, we analyzed the relationships between the cell position and division activity in the same population. Within each sector, marginal cells generally showed higher division activity than inner cells during the 30–108 h time period (Fig. 6b). For example, after one round of division, a new cell located at the marginal layer (solid circles) had higher division activity than its sibling located at the inner layer (circles filled with diagonal stripes) during the following time period (Fig. 6b). These results also aligned with the quantified division events within 54–108 h time frame, the maturation phase (Supplementary Fig. 10; Supplementary Data 5), suggesting that position plays a role in determining division activity of meristem cells during meristem proliferation. Such patterns were consistent among all the analyzed meristem progenitor cells from three independent gametophyte samples (Fig. 6a, b; Supplementary Figs. 11a, b, 12a, b), suggesting that cell division within the meristems is regulated by positional cues, likely initiated from the marginal layer of the meristem.

### Origin and fate of cells forming differentiated archegonia

In Ceratopteris gametophytes, once a multicellular meristem is established, egg-forming archegonia always initiate near the meristem notch (Fig. 2; Supplementary Figs. 4 and 5). To identify the cell or population of cells from which an archegonium arises, we quantitatively analyzed cell behaviors underlying archegonium initiation and maturation in the three independent gametophyte samples (Fig. 2; Supplementary Figs. 4 and 5). The first archegonium of each gametophyte started to initiate at 54–60 h and then developed into a multicellular structure after several rounds of cell divisions (highlighted with orange dotted circles in Fig. 2j–v; Supplementary Figs. 4j–v, 5k–v). After around 30 h, the second archegonium (highlighted with blue dotted circles in Fig. 2o–v; Supplementary Figs. 4p–v, 5o–v) initiated and then followed similar cell division patterns. We then examined the origins of these archegonia. To our surprise, although they both were specified next to the center of the meristem, these two sequentially formed archegonia had different origins (Fig. 3k, p; Supplementary Figs. 5k, q, 6l, p). The first archegonium of each gametophyte belonged to one cell lineage that was adjacent to the meristem but did not mainly contribute to meristem proliferation (Fig. 3j, k; Supplementary Figs. 6j, k, 7k, l), whereas the second one shared the same lineage with a group of cells composing the meristem (Fig. 3o, p; Supplementary Figs. 6p, q, 7o, p). These results demonstrate that not all of the archegonia are derived from the meristem lineages.

**Fig. 7:**
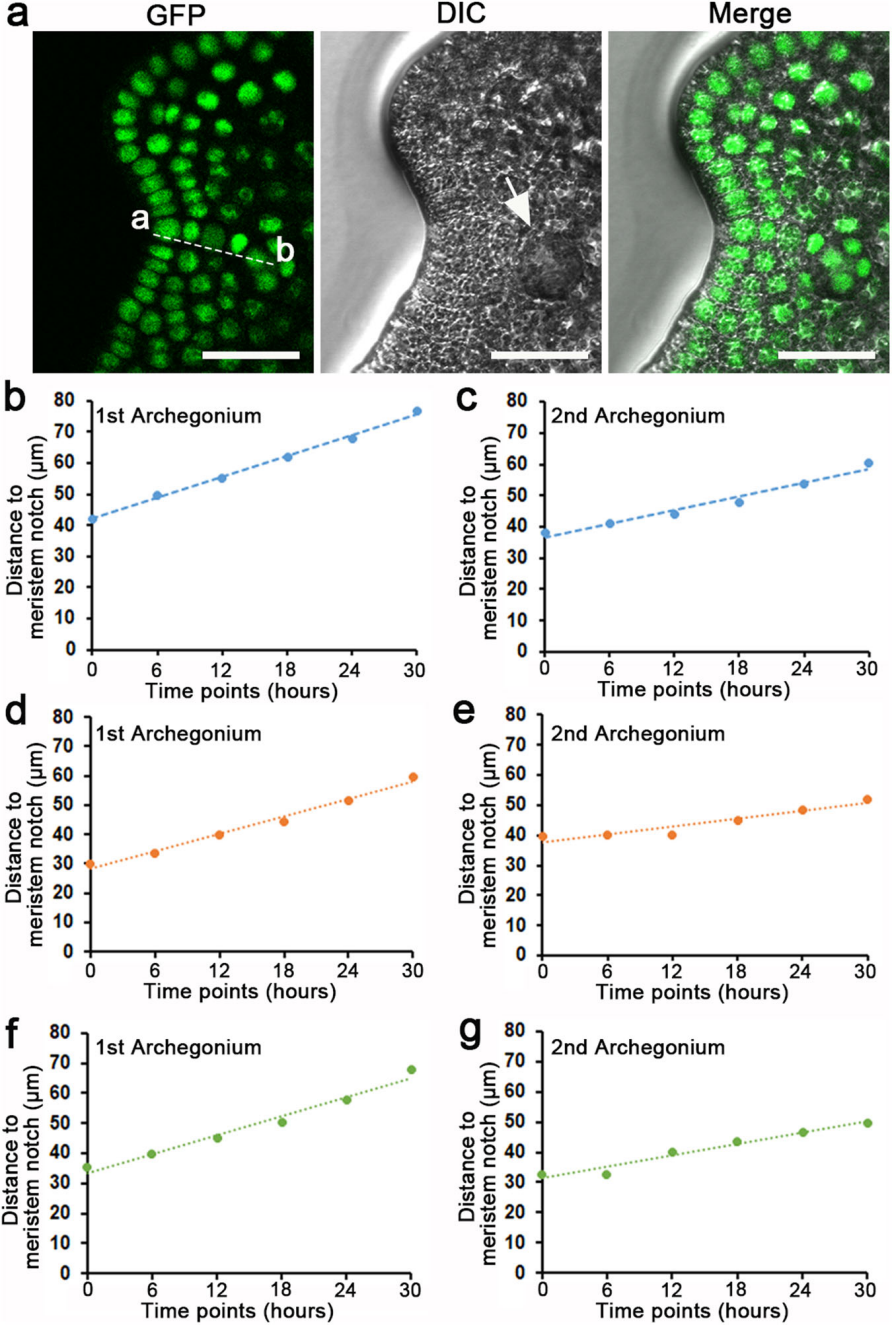
The dynamic distance between archegonia and the meristem notch during gametophyte development. **a** The calculation of distance between a meristem notch (or the location where the meristem notch will be formed) (point a) and the center of an archegonium (point b) in one representative image. The image in (**a**) is the part of one optical section of the sample shown in Fig. 2m. Scale bar: 50 μm. **b**–**g** Dynamic distances between archegonia and meristem notches. *Y* axis: Distance between a meristem notch and an archegonium. *X* axis: six consecutive time points starting from initiation (shown as 0 h in the graphs) of the archegonium. The distance in **b** and **c** was calculated based on the confocal images of the gametophyte shown in Fig. 2, and the first and second archegonia started to initiate at 54 h and 84 h, respectively. The distance in **d** and **e** was calculated based on the confocal images of the gametophyte shown in Supplementary Fig. 4, and the first and second archegonia started to initiate at 54 h and 90 h, respectively. The distance in **f** and **g** was calculated based on the confocal images of the gametophyte shown in Supplementary Fig. 5, and the first and second archegonia started to initiate at 60 h and 84 h, respectively. The source data for (**b**–**g**) are included in Supplementary Data 4.

To further test the dynamics of archegonium initiation and maturation, we quantified the distance between the meristem notch and the archegonia during gametophyte development (Fig. 7a–g; Supplementary Data 4). We found that the first archegonium was specified at the place close to the center of the meristem notch, with an average distance of 35.8 ± 3.5 μm (mean ± standard error, *n* = 3) away from the initiation site of the notch (indicated by ‘a’ in Fig. 7a). After that, the distance between the meristem notch and the center of the first archegonium gradually increased (Fig. 7b, d, f). Interestingly, the second archegonium also initiated adjacent to the center of the meristem notch, with an average distance of 36.7 ± 2.1 μm (mean ± standard error, *n* = 3) away from the notch, and then gradually moved away from the meristem (Fig. 7c, e, g). These quantitative results demonstrate that archegonia, regardless of their lineages, are initiated in close proximity to the meristem notch.

### Cell fates and cell division dynamics during ablation-induced *de novo* formation of a new meristem

In addition to normal growth, we further dissected how perturbing the gametophyte affected the cell fate and proliferation in gametophytes. Earlier studies showed that, once their multicellular meristems were removed, prothalli in several fern species (such as *Anemia phyllitidis*, *Pteris longigolia* and *Gymnogramme chrysophyll*) were able to regenerate new meristems on the remaining meristem-less prothalli^45–47^. We performed microsurgical experiments to ablate only a few cells in the meristems or at the initiation sites of meristems in wild-type hermaphroditic gametophytes in Ceratopteris (*n* = 5). Once the meristem was ablated, at least one new meristem regenerated from each prothallus (Supplementary Fig. 13). These results not only showed that Ceratopteris prothalli possess the regeneration capacity similar to other ferns, but, more importantly, demonstrated a robust approach for us to determine cell behaviors during *de novo* formation of new meristems in gametophytes.

**Fig. 8:**
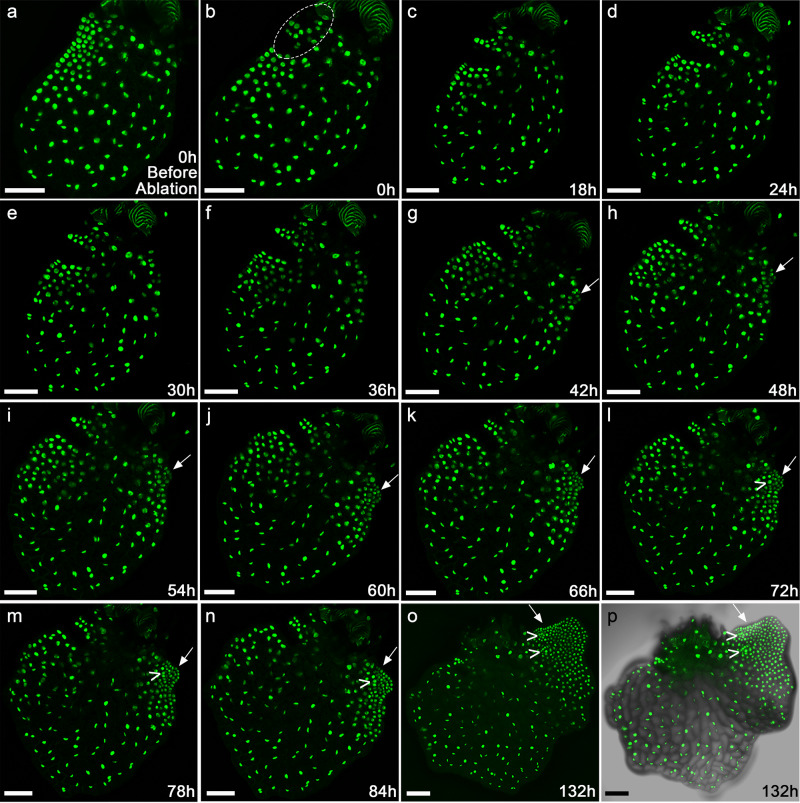
Time-lapse confocal imaging of the ablation-induced new meristem formation in the Ceratopteris gametophyte. **a**–**o** The meristem in one hermaphroditic gametophyte expressing the nuclear marker H2B-GFP (at 8 DAI) was disrupted by mechanical ablation. The *de novo* formation of a new meristem in response to ablation was live-imaged by laser scanning confocal microscopy. **a**–**o** Z-projection views of confocal images taken at the indicated time points before (**a**) and after (**b**–**o**) the ablation. The time-lapse imaging was performed at the 6-hour interval, from 0 h to 132 h. The ablated area is highlighted in **b**. The white arrow indicates the newly initiated meristem after the ablation. The “V” indicates the archegonium formed adjacent to the newly initiated meristem. **p** Merge of the GFP (**o**) and DIC (showing the cell outlines) channels. Scale bars: 100 μm. Three independent biological replicates were included in the mechanical ablation and time-lapse imaging experiment with the same interval and duration, all showing the *de novo* formation of new meristems and archegonia.

We then examined real-time cell dynamics during the ablation-induced new meristem formation by live-imaging Ceratopteris gametophytes expressing the H2B-GFP reporter (Fig. 8a–p). At 8 DAI when the meristem was distinguishable, the hermaphroditic gametophyte (*n* = 3) was imaged before and immediately after ablating a few cells within the meristem (as 0 h). Time-lapse imaging was then performed at six-hour intervals, with full stacks of optical sections acquired (with one representative sample shown in Fig. 8). The Z-projection view showed that disruption of the established meristem led to *de novo* formation of a new meristem (indicated by arrows in Fig. 8g–p). As the control, a similar microsurgical perturbation was performed to ablate a few cells at a non-meristematic region of the hermaphroditic gametophyte (*n* = 3). After wounding, the time-lapse imaging was performed with the same interval and duration (Supplementary Fig. 14a–p). The mechanical ablation of non-meristematic regions did not cause any noticeable change of meristem proliferation and notch formation in these gametophytes (Supplementary Fig. 14a–p), demonstrating that the switch of growth patterns is not a response to wounding in general but is specific to ablation of the meristem.

**Fig. 9:**
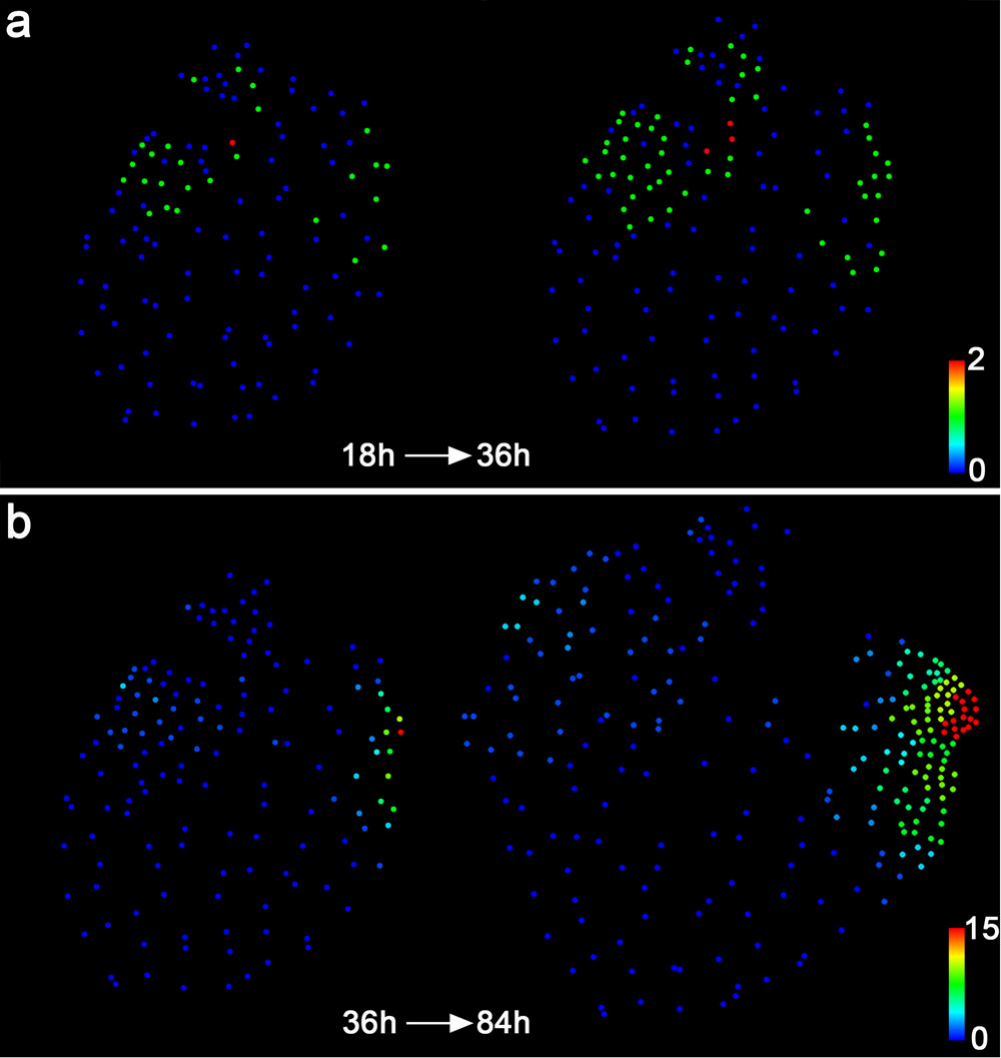
Quantification of cell division events in the ablated Ceratopteris gametophyte. Each dot represents the location of the segmented individual nucleus shown in Fig. 8. Quantification of cell division events in the following time frames from 18–36 h (**a**) and 36–84 h (**b**). The quantified cell division events are shown in the images from the first (left panel) and last (right panel) time points of the indicated intervals. Colors indicate the number of cell division events in each cell lineage during the indicated time intervals, with the scale from blue (0) to red (2) (**a**) and from blue (0) to red (15) (**b**).

We also performed nucleus segmentation, lineage analysis (Supplementary Fig. 15) and quantification of division activities (Fig. 9a, b; Supplementary Fig. 16) on the representative gametophyte shown in Fig. 8. These results showed that the division activity of cells surrounding the ablated meristem dropped rapidly (Fig. 9a, b). As mentioned above, during normal developmental process of hermaphroditic gametophytes, cells located outside of the meristem gradually lost division activity once the meristem was well established (Figs. 4c–i, 5c, d). However, after ablating the meristem, a few marginal cells located on the non-ablated region of the prothallus re-gained division activity, forming a few actively proliferating lineages (Fig. 9; Supplementary Figs. 15 and 16). Their descendants formed a new meristem, with the center of active dividing zones having shifted from the original meristem to the newly initiated meristematic region (Fig. 9a, b). Taken together, these results show that ablation of the meristem promotes non-meristematic cells to regain division activity and eventually form a new meristem.

### Meristem provides a positional cue that determines initiation of archegonia

The live-imaging results (Fig. 2; Supplementary Figs. 4 and 5) led to a hypothesis that the meristem promotes archegonium initiation in its surrounding cells, likely via a position dependent way. To test this hypothesis, we examined the patterns of archegonium initiation and maturation in the eight gametophyte samples after ablating their meristems, which included three transgenic gametophytes expressing the H2B-GFP reporter (with one representative gametophyte shown in Fig. 8) and five wild-type gametophytes (with one representative gametophyte shown in Supplementary Fig. 13). In line with the hypothesis, live-imaging results showed that once the original meristem was ablated, the initiation of new archegonia around the ablated meristem was rapidly abolished (Fig. 7; Supplementary Fig. 13); after the new meristem formed *de novo* at a different location, new archegonia initiated at the position adjacent to the new meristem and continued to develop through sustained cell division over time (Fig.7; Supplementary Fig. 13). Interestingly, if there were already specified archegonia existing in the prothalli at the time of the ablation, these determined archegonium cells were able to continue the cell division and complete the maturation process after ablating the meristem (*n* = 3, with one representative gametophyte shown in Supplementary Fig. 17). However, the cells surrounding the ablated meristem no longer differentiated new archegonia regardless of whether any mature archegonia had existed nearby (*n* = 3, with one representative gametophyte shown in Supplementary Fig. 17), suggesting that archegonia themselves were not sufficient to promote the initiation of new archegonia. Collectively, these results indicate that the meristem provides a positional cue that is required for the initiation of archegonia from the cells next to the meristem notch.

## Discussion

The life cycle of land plants alternates between the sexual gametophyte and asexual sporophyte phases^1,2^. Indeterminate meristems have evolved in gametophytes from bryophytes and ferns, and sporophytes from vascular plants^8^. In this study, through quantitative live imaging, we reconstruct cell lineage and real-time dynamics of cell division during initiation and proliferation of meristems in gametophytes of the model fern *Ceratopteris richardii*, suggesting both conserved and unique features and regulations in the meristem of Ceratopteris gametophytes, in comparison to other types of indeterminate meristems in land plants.

**Fig. 10:**
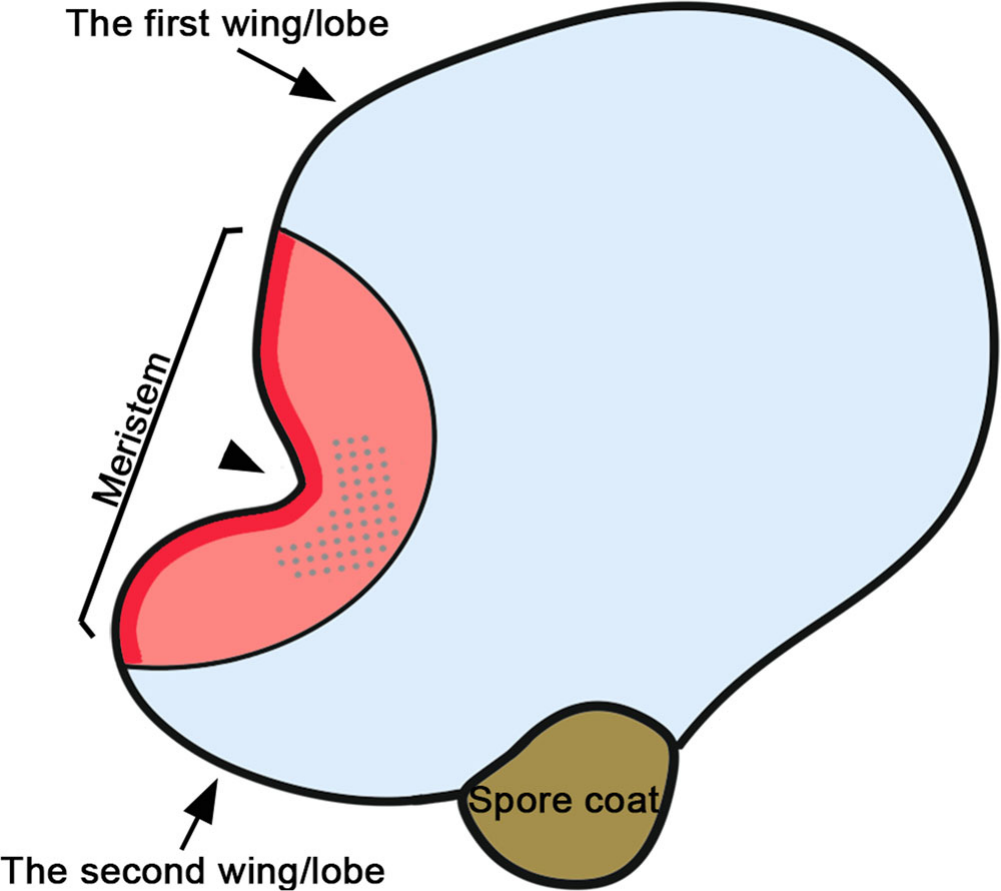
A diagram illustrates a developing Ceratopteris gametophyte with a meristem and functional zones. Based on the long-term time-lapse imaging and cell division analyses, different functional zones in Ceratopteris hermaphroditic gametophytes are defined. The multicellular meristem in Ceratopteris gametophytes is maintained as an actively dividing zone, compared with the mitotically inactive cell groups outside of the meristem. The red and pink area represents the zone of cell division where cells undergo active mitotic division to either renew themselves or move away from the meristem notch (as pointed with the black arrowhead). The red area represents the zone of marginal cells within the meristem, maintaining higher division activity than inner cells. The dotted pink area represents the zone of division and archegonium initiation where the archegonium progenitor cells are specified adjacent to the meristem notch. Each progenitor cell undergoes multiple rounds of cell division to finally develop into a multicellular mature archegonium. The blue area represents the zone of cell expansion where most cells stop dividing (but increasing in size). In general, the multicellular meristem promotes quick proliferation of the second, less developed wing, eventually resulting in a heart-shaped prothallus.

Our long-term time-lapse imaging of developing gametophytes and quantitative analysis demonstrate that the meristem of Ceratopteris gametophytes is devoid of a distinguishable central zone or apical cell with low cell division activity compared to the surrounding cells (Figs. 4, 5; Supplementary Figs. 8, 9; illustrated in Fig. 10). Within the meristem, marginal cells show significantly higher division activity than inner cells, regardless of their lineages (Supplementary Fig. 10; illustrated in Fig. 10). In addition, division of meristem progenitor cells in Ceratopteris gametophytes does not follow a predictable orientation or invariable pattern (Fig. 6a; Supplementary Figs. 11a and 12a), which is similar to the post-embryonic cell division instead of embryonic cell division in flowering plants^48^. All these findings are different from the previous interpretation of Ceratopteris meristem development based on the observation of different fixed gametophyte samples harvested at different growth stages^22^.

Meristem organization in Ceratopteris gametophytes is different from that in the gametophytes of bryophytes and several other fern species and is also different from that in the sporophytes of lycophytes and ferns, in which wedge-shaped apical cells (ACs) play a key role in sustaining cell proliferation^9,10,23,25,49–55^. Even though the meristems from several different species are morphologically comparable, the underlying cellular bases seem to be different. For example, ACs drive proliferation of notch meristems in thalli of the liverwort *Marchantia polymorpha* (hereafter ‘Marchantia’) and in young prothalli of the fern *Colysis decurrens*^25,56,57^, whereas the formation of meristem notch in Ceratopteris gametophytes is independent of an AC. In addition, meristem organization in Ceratopteris gametophytes is also different from that in the sporophytes of flowering plants (such as Arabidopsis). The sporophytes of flowering plants develop shoot apical meristems and root apical meristems, which do not contain a distinguishable AC. However, shoot apical meristems maintain a group of slowly dividing undifferentiated stem cells as the conserved central zone^3,15,17^, and root apical meristems also contain rarely dividing stem cells that compose the quiescent center^3,58^. Thus, the distinct organization and cell behavior identified in the meristem of Ceratopteris gametophytes advance our understanding of diversified meristem development in land plants.

In land plants, continuous organ initiation is largely dependent on meristem activity, despite that the process is achieved via different ways. In this study, quantitative live imaging during both normal and disturbed growth suggests that the multicellular meristem in Ceratopteris gametophytes promotes organogenesis—archegonium formation—in its surrounding cells, via a position-dependent manner (Figs. 7 and 8; Supplementary Figs. 13 and 15). The initiation of archegonia likely relies on positional cues triggered by the multicellular meristem instead of the lineage of initial cells, which is different from the AC-dominant organ formation reported in a few seed-free plants. For example, in gametophytes of the moss *Physcomitrium (Physcomitrella) patens* and in sporophytes of the lycophyte *Selaginella kraussiana* and the fern *Nephrolepis exaltata*, histological and clonal analyses show that a single or few ACs cleave in different facets to produce initial cells, and each of them follows predictable cell fates and finally develops into a whole organ (e.g., leaf-like organs or fronds)^9,53–55^. Interestingly, in gametophytes of the liverwort Marchantia, although the flattened thallus also grows from ACs in an apical notch, the apical notch specifies growth rates in surrounding cells, likely through a diffusive morphogen^57^. In addition, the formation of air chambers and gemma cups in Marchantia thalli likely relies on positional information rather than cell lineages^59^. Furthermore, the position-dependent organ differentiation is prevalent in sporophytes of flowering plants^60–62^. For example, the localized concentration of the phytohormone auxin dictates primordium initiation in shoot apical meristems with conserved phyllotactic patterns^63,64^. Thus, it seems that the position-dependent and lineage-independent organ formation mechanism is shared among several different systems, including Marchantia gametophytes, Ceratopteris gametophytes, and angiosperm sporophytes. Future studies are needed to better interpret the positional cue that determines archegonium initiation in Ceratopteris gametophytes.

This study also uncovers the spatial-temporal dynamics of archegonium development, from initiation to maturation, which provides insights into key strategies that ferns have developed to facilitate fertilization. Different from seed plants, the efficiency and success of fertilization in ferns are limited by many factors. For example, fern gametophytes grow independently of their sporophytes^9,11^, with limited protection against mechanical damage. In addition, in the absence of water or other media to swim through, the motile sperm is not able to fertilize the egg^11,12^. Moreover, in Ceratopteris, one archegonium produces one egg and each egg is only viable for less than 48 hours if it is not fertilized^11^. The time-lapse imaging and quantitative results in this study demonstrate that once a hermaphroditic gametophyte develops the multicellular meristem, it continuously and sequentially initiates multiple archegonia surrounding the meristem (Fig. 2; Supplementary Figs. 4 and 5). The time interval between the initiation of first two archegonia is less than 36 h (Fig. 2j, o; Supplementary Figs. 4j, p, 5k, o). As the prothallus further proliferates, the interval between the initiation of the following two archegonia is even shorter. Thus, the meristem drives the indeterminate growth of prothalli and the constant formation of new archegonia, which ensures that there is always an egg available for fertilization throughout gametophyte development. In addition, once the meristem is damaged, the hermaphroditic gametophyte quickly reactivates cell proliferation in a non-wounded region and regenerates at least a new meristem there (Fig. 8). The newly initiated meristem then induces continuous initiation of archegonia (Fig. 8). This strategy also sustains egg formation until fertilization and helps to overcome hurdles of fertilization in response to potential damage to the hermaphroditic gametophyte.

The quantitative pipeline established in this work integrates long-term time-lapse imaging, reconstruction and visualization of lineage dynamics, and quantification of division activities at high spatiotemporal resolution, in both undifferentiated meristems and differentiated archegonia in Ceratopteris gametophytes. This pipeline can be broadly used and adapted for determining lineage and fate alterations in fern gametophytes in response to various developmental cues and environmental signals, or in mutants and transgenic lines with the genetic perturbation^20,21,28,29,31^. In addition, future studies incorporating cell growth dynamics into the cell division and lineage datasets will reveal the cellular basis of shape generation (e.g., notch formation) in Ceratopteris gametophytes and provide a comprehensive cell atlas of gametophyte development in ferns.

## Methods

### Plant materials and growth conditions

*Ceratopteris richardii* strain Hn-n^40^ was used in this study to generate the transgenic plants. Gametophytes were grown on FM plates (pH 6.0) containing 0.5 × MS salts (PhytoTechnology Laboratories) and 0.7% (w/v) agar (Sigma-Aldrich). Sporophytes were formed on fertilized gametophytes and were transferred to soil, typically after 3–4 weeks of fertilization. Both gametophytes and young sporophytes were grown under continuous light at 28 °C. Adult sporophytes were grown in the LILY greenhouse facility at Purdue for harvesting spores.

Ceratopteris calli were induced from young sporophytes (shoot tips or fronds) on the callus induction medium (pH 5.8) that contains 1 × MS salts (PhytoTechnology Laboratories), 2% (w/v) sucrose, 1 mg/L benzylaminopurine (BAP), and 0.7% agar (Sigma–Aldrich). Calli were cultivated under continuous light at 28 °C in the growth chamber (Percival).

### DNA constructs and plant transformation

To generate a fluorescent nuclear reporter specific for *Ceratopteris richardii*, the *pCrHAM::H2B-GFP::3’CrHAM* expression cassette was constructed, in which the *Histone 2B (H2B)-GFP* fusion is driven by the endogenous 5’ promoter and 3’ terminator of the *CrHAM* gene. Specifically, the *H2B-GFP* DNA fragment was described previously^42^. A 1014-bp *CrHAM* 3’ terminator (*3’CrHAM*) was amplified from the *Ceratopteris* genome using the primers 5’-TACAggcgcgccAAGGTAGTTGATATAAGACGTT-3’ and 5’- TACAggcgcgccTGCTACTCGGAACTTAATATCTACCC-3’ and cloned into the 3’ end of the *H2B-GFP* fragment using the restriction enzyme digestion and ligation (restriction recognition sites are in lower case). Then, a 2272-bp *CrHAM* promoter was amplified from the *Ceratopteris* genome using the primers 5’-ACAAgcggccgc GTTGTTTGGATACTTAGGTGGAGG-3’ and 5’- ACAAgcggccgcTCAAAAGGATCAAACCCAAAATGCA-3’ and cloned into the 5’ end of the *H2B-GFP::3’CrHAM* fragment. Sequence of the *pCrHAM::H2B-GFP::3’CrHAM* expression cassette is shown in Supplementary Fig. 18. The *pCrHAM::H2B-GFP::3’CrHAM* fragment was then introduced into the pMOA34 binary vector.

To generate transgenic lines, the pMOA34 *pCrHAM::H2B-GFP::3’CrHAM* vector was transformed into *Ceratopteris* calli through the microparticle bombardment following the detailed procedure described previously^65,66^. Bombardment was performed using the Bio-Rad Biolistic PDS-1000/He particle delivery system. Plasmid-coated tungsten microparticles were delivered at 1100 psi. The regenerated T_0_ sporophytes from calli were selected based on their hygromycin resistance. The spores from each individual T_0_ sporophyte were harvested and the stable transformation of the construct in these lines was confirmed through testing the hygromycin resistance in their T_1_ gametophytes. The expression of H2B-GFP was also determined in the T_1_ gametophytes using a Zeiss LSM880 upright confocal microscope. At least three independent transgenic lines (including the line 12, line 24 and line 45) showed comparable expression levels and patterns in the *Ceratopteris* gametophytes. As shown in Fig. 1 (for the line 24) and Supplementary Fig. 3 (for the line 12 and line 45), *pCrHAM::H2B-GFP::3’CrHAM* was highly and ubiquitously expressed in the transgenic gametophytes (except in gametes) at the indicated days after inoculation in this study, which was consistent with the previous report that the *AtHAM2* (Arabidopsis *HAM2*) transcriptional reporter is constitutively expressed in Arabidopsis shoot apical meristems and primordia^42^. The reporter line 24 of *pCrHAM::H2B-GFP::3’CrHAM* (shown in Fig. 1) was used for all the cell division analyses and mechanical perturbation experiments.

### Sample preparation and live imaging

Spores of *Ceratopteris* transgenic plants were surface sterilized and sown on FM plates to produce gametophytes. The FM plates were sealed in Ziploc bags to maintain humidity and grown in the Percival growth chamber with the settings of continuous light, 28 °C and 80% humidity. Gametophytes from 5–16 DAI were imaged in this study (described specifically in figure legends). To visualize cell morphology, gametophytes were stained with propidium iodide (PI) for 1 min. Then, they were rinsed with sterilized water two or three times and transferred to new FM plates for imaging. To perform the mechanical ablation, a few cells in the meristem (as shown in Fig. 7; Supplementary Figs. 13 and 15) or at a non-meristematic region (as shown in Supplementary Fig. 14) from a hermaphroditic gametophyte were pierced under a Nikon SMZ1000 stereoscope, using a sterilized micro-needle (Electron Microscopy Sciences). The confocal images of the same sample immediately before and after the ablation were taken for the comparison. For the time-lapse imaging, gametophytes were transferred to new FM plates and imaged every 6 h, which was sufficient to capture each cell division event. After imaging, these FM plates were sealed in Ziploc bags again and moved back to the growth chamber (Percival) that was located next to the confocal microscope, and the samples were grown under the same condition until the next time point.

Gametophytes shown in Supplementary Figs. 2 and 13 were imaged on FM plates using a stereoscope with a digital camera MU1803. All the other gametophytes were imaged using a Zeiss LSM880 upright confocal microscope. The settings of confocal imaging in Zen black software (Zeiss) were described previously in detail^67^ with a few modifications in this study. Specifically, all the gametophytes were live-imaged on FM plates, using a Plan-Apochromat 10×/0.45 objective lens. Scanning interval of confocal optical sections was set to 1.0 μm for all the samples except 0.45 μm for imaging Archegonia with high resolution (Fig. 1e–g). For the confocal snapshots, GFP was excited using a 488-nm laser line and the emission was collected from the 491–562 nm. The detector gain for the GFP signal was set within the range of 769–782 and the detector digital gain was 1.0. PI was excited using a 514-nm laser line and the emission was collected from 587–669 nm. The detector gain for the PI signal was set within a range of 569–620 and the detector digital gain was 1.0. For time-lapse imaging, GFP was excited using a 488-nm laser line with the detection wavelength from 491–562 nm. The detector gain was set within a range of 769–780 and the detector digital gain was 1.0. The confocal images were processed using the Fiji/ Image J software to generate maximum intensity projection (z-projection) views with slight adjustment of brightness and contrast.

### DAPI stain and confocal imaging

To confirm nuclear localization of H2B-GFP protein, Ceratopteris gametophytes expressing the *pCrHAM::H2B-GFP::3’CrHAM* reporter were stained with 4′,6-diamidine-2′-phenylindole dihydrochloride (DAPI) and imaged through the ZEISS 880 confocal microscope (shown in Supplementary Fig. 1). Specifically, the gametophytes were briefly treated with ethanol for ~3 min and then stained with DAPI (Sigma-Aldrich) for 3 min. After that, the stained gametophytes were rinsed with sterilized water and imaged on FM plates. DAPI was excited using a 405-nm laser line with the detection wavelength from 436–475 nm. GFP was excited using a 488-nm laser line with the detection wavelength from 490–553 nm. The DIC channel was also collected for the visualization of the gametophyte cell outline. Scanning interval of confocal optical sections was set to 0.8 μm. The merge of GFP and DAPI channels was generated in Fiji / Image J.

### Statistics and reproducibility

The statistical significance between two groups (shown in Supplementary Fig. 10d) was evaluated by Student’s two-tailed *t*-test. The sample sizes for each experiment are indicated in the figure legends. Source data files for each graph are included in Supplementary Data.

### Nucleus segmentation and detection, cell lineage and division analysis

The pipeline using Matlab software for the nucleus segmentation and detection and for the quantitative analyses of cell lineage and division consists of three parts. First, Ceratopteris prothalli develop as a flat sheet of cells (Supplementary Movies 1 and 2), which are suitable for the 2D imaging analysis. Nucleus segmentation was carried out on confocal images with the maximum intensity projection (z-projection) of the H2B-GFP reporter signal, using an established watershed method with distance transform^68^. The watershed was performed using the built-in implementation of Matlab following the manual (MATLAB, MathWorks) and the code is available upon request. An immature (developing) archegonium consisted of only a few nuclei, which can be visualized from the z-projection view of the confocal stacks (as examples shown in Fig. 1e–g and indicated in Fig. 2j–k) and then segmented and labeled (as indicated in Fig. 3j–k). In contrast, as characterized previously^39^, a mature archegonium formed the complex 3D structure (after 108 h of the live imaging in this study, as shown in Fig. 2v–w), which was not analyzed in this study. One example of complete nucleus segmentation and identification from 0 h to 108 h was shown in Supplementary Fig. 19. Then, the unique label was automatically assigned to each segmented nucleus, and one small circle was placed at the center of each segmented nucleus to define and mark the nucleus location within the gametophyte. The errors in nucleus segmentation were corrected through the deletion, merging, or separation of nuclei using Matlab software. Second, for the lineage analysis, cell lineage files were manually generated for each of two consecutive time points over the whole-time frame (e.g., the first lineage file from 0 h to 6 h and the second lineage file from 6 h to 12 h over the 108-hour frame for the segmented sample shown in Supplementary Fig. 19). Based on the cell lineage files, all descendants from each progenitor cell were tracked and recorded at all the subsequent time points within the analyzed time frame. Different colors were randomly assigned to different cell lineages to generate the lineage maps, representing the progression of individual progenitor cells over time (as shown in Fig. 3). In the third part of the pipeline, the numbers of cell division events for the cells originated from the same progenitor cell were quantified as shown in Supplementary Data 1, based on the cell lineages at six-hour intervals. The total number of cell division events for each cell lineage was quantitatively indicated by color, with the range from blue (zero division event) to red (highest number of division events) (as shown in Fig. 5). The scales of each color bar and the time frames for each division map were specified in the figures and figure legends.

### Reporting summary

Further information on research design is available in the Nature Research Reporting Summary linked to this article.

## Supplementary information

Supplementary information

Description of Additional Supplementary Files

Supplementary Data 1

Supplementary Data 2

Supplementary Data 3

Supplementary Data 4

Supplementary Data 5

Supplementary Movie 1

Supplementary Movie 2

Supplementary Movie 3

Reporting Summary

## Data Availability

The data that support the results and conclusions of this study are available within the paper, Supplementary Information and Supplementary Data 1–5. DNA sequence of the expression cassette for the *pCrHAM::H2B-GFP::3’CrHAM* reporter was deposited in NCBI with the accession number ON787967 and is also shown in Supplementary Fig. 18. Any other supporting information is available from the corresponding author upon request.

## Code availability

The code is available from the corresponding author upon request.
