# Supplementary material for: Positional cues and cell division dynamics drive meristem development and archegonium formation in Ceratopteris gametophytes
Source: Commun Biol. 2022 Jul 1;5:650. doi: 10.1038/s42003-022-03627-y (PMC9249879; doi:10.1038/s42003-022-03627-y)

Positional cues and cell division dynamics drive meristem development and archegonium formation in Ceratopteris gametophytes

Yuan Geng[1,2], An Yan[3,4], Yun Zhou[1,2 *]

[1]Department of Botany and Plant Pathology, Purdue University, West Lafayette, IN 47907, USA
[2]Purdue Center for Plant Biology, Purdue University, West Lafayette, IN 47907, USA
[3]Division of Biology and Biological Engineering, California Institute of Technology, Pasadena, CA 91125, USA
[4]Howard Hughes Medical Institute, California Institute of Technology, Pasadena, CA 91125, USA

*To whom correspondence should be addressed: zhouyun@purdue.edu

Supplementary Figures 1-19

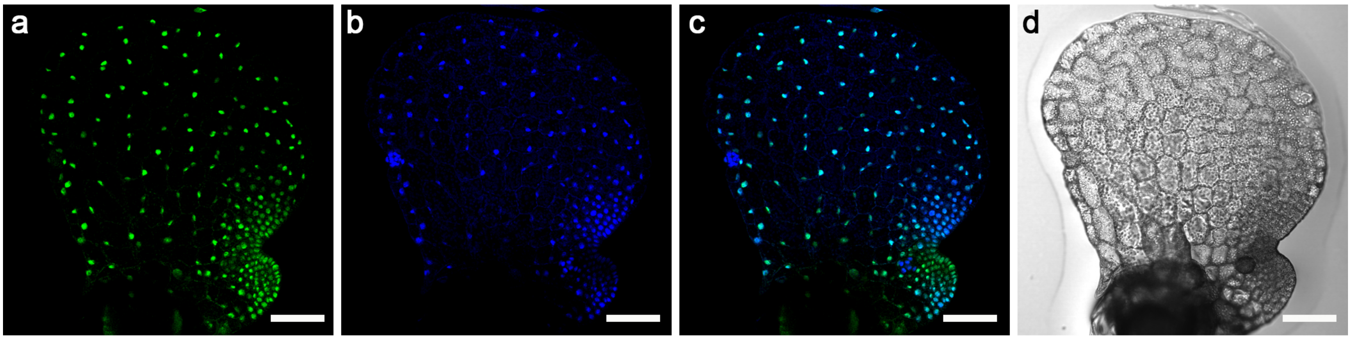

Supplementary Fig. 1 Nuclear localization of Histone 2B (H2B)-GFP in Ceratopteris gametophytes. A transgenic hermaphroditic gametophyte expressing H2B-GFP was stained with 4′,6-diamidine-2′-phenylindole dihydrochloride (DAPI) and imaged at 8 days after inoculation (DAI) using laser scanning confocal microscopy. (a) A Z-projection view of GFP-labeled nuclei; (b) A Z-projection view of DAPI-stained nuclei; (c) Merge of (a) and (b); (d) DIC channel showing cell outlines of the same gametophyte. Scale bars: 100 μm.

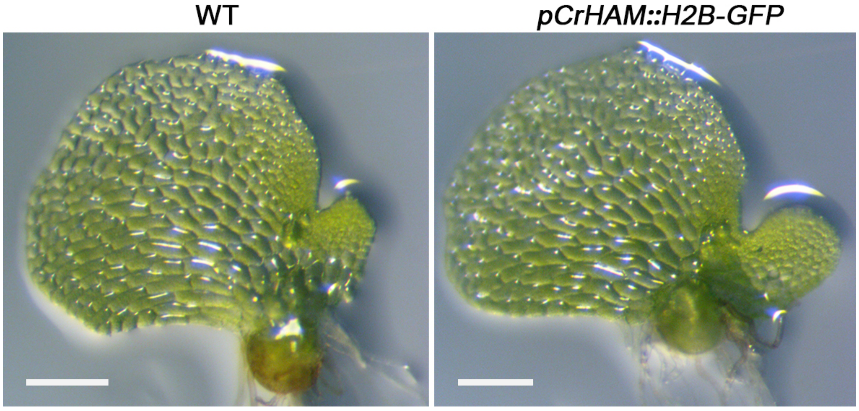

Supplementary Fig. 2 Ceratopteris wild-type and transgenic hermaphroditic gametophytes show comparable morphology. Hermaphroditic gametophytes were imaged at 9 DAI. Scale bars: 0.2 mm.

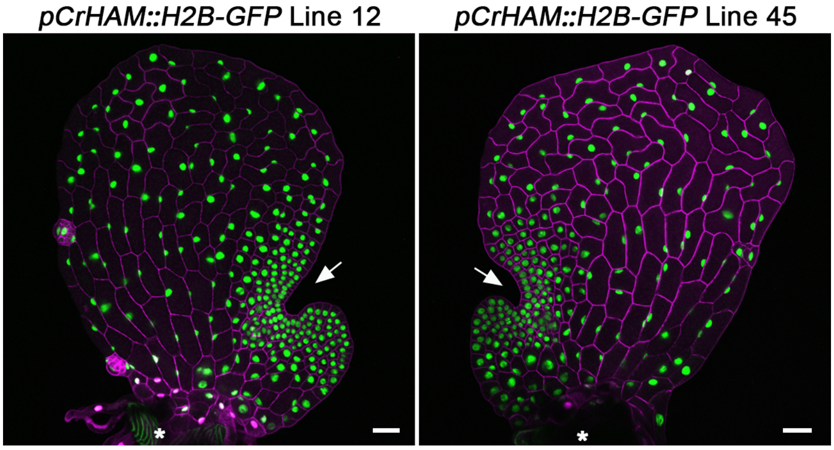

Supplementary Fig. 3 Confocal imaging of independent transgenic lines expressing the nuclear marker H2B-GFP. Hermaphroditic gametophytes of transgenic line 12 and line 45 (the other two independent reporter lines) were stained with propidium iodide (PI) and imaged at 9 DAI using laser scanning confocal microscopy. The white arrow indicates the meristem notch of each gametophyte. Asterisks indicates areas where spore coats are located. Green: H2B-GFP; Purple: PI stain. Scale bars: 50 µm.

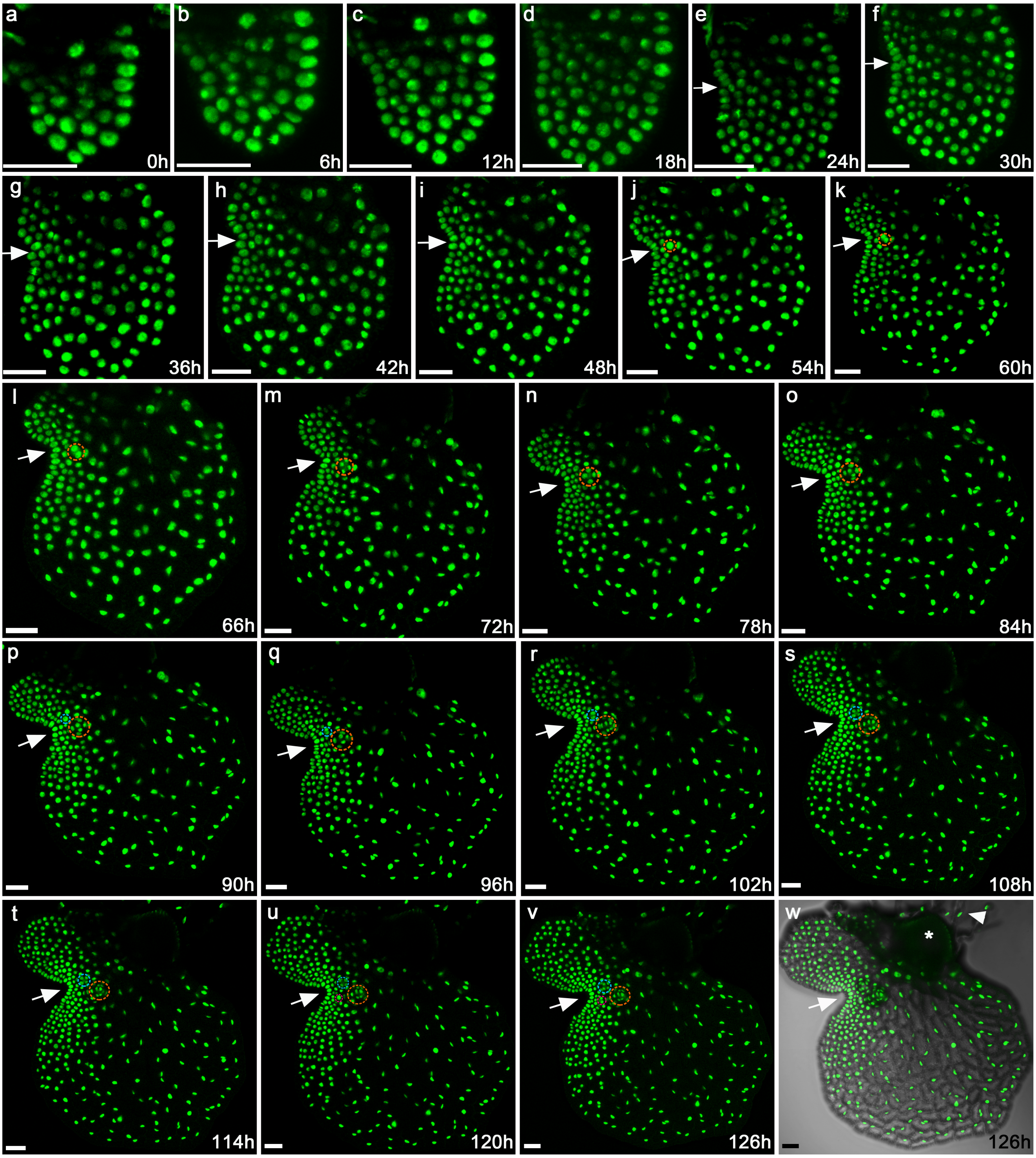

Supplementary Fig. 4 The second independent biological replicate included in the time-lapse imaging experiment. (a-v) The hermaphroditic gametophyte (at 5 DAI) was live-imaged through laser scanning confocal microscopy. The imaging was performed at 6-hour intervals. (a-v) Z-projection views of confocal images at the indicated time points are shown. White arrows indicate meristem initiation and proliferation over time. Three archegonia subsequently formed next to the meristem, which are highlighted with the circles in different colors (orange, blue and pink). (w) Merge of the GFP (v) and DIC (showing the cell outlines) channels. The white asterisk indicates ruptured spore coat and the white arrowhead indicates rhizoids. Scale bars: 50 μm.

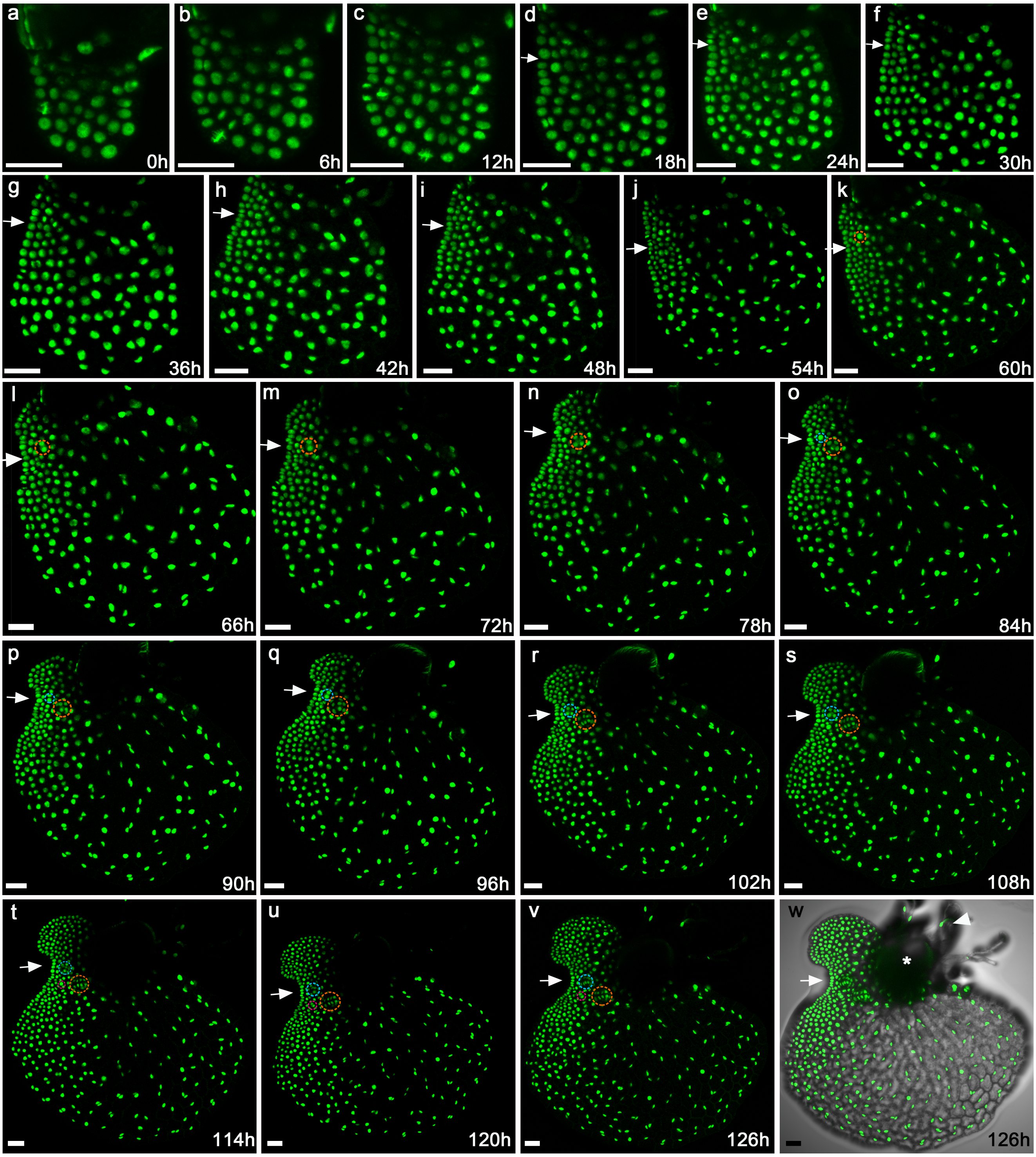

Supplementary Fig. 5 The third independent biological replicate included in the time-lapse imaging experiment. (a-v) The hermaphroditic gametophyte (at 5 DAI) was live-imaged through laser scanning confocal microscopy. The imaging was performed at 6-hour intervals. (a-v) Z-projection views of confocal images at the indicated time points are shown. White arrows indicate meristem initiation and proliferation over time. Three archegonia subsequently formed next to the meristem, which are highlighted with the circles in different colors (orange, blue and pink). (w) Merge of the GFP (v) and DIC (showing the cell outlines) channels. The white asterisk indicates ruptured spore coat and the white arrowhead indicates rhizoids. Scale bars: 50 μm.

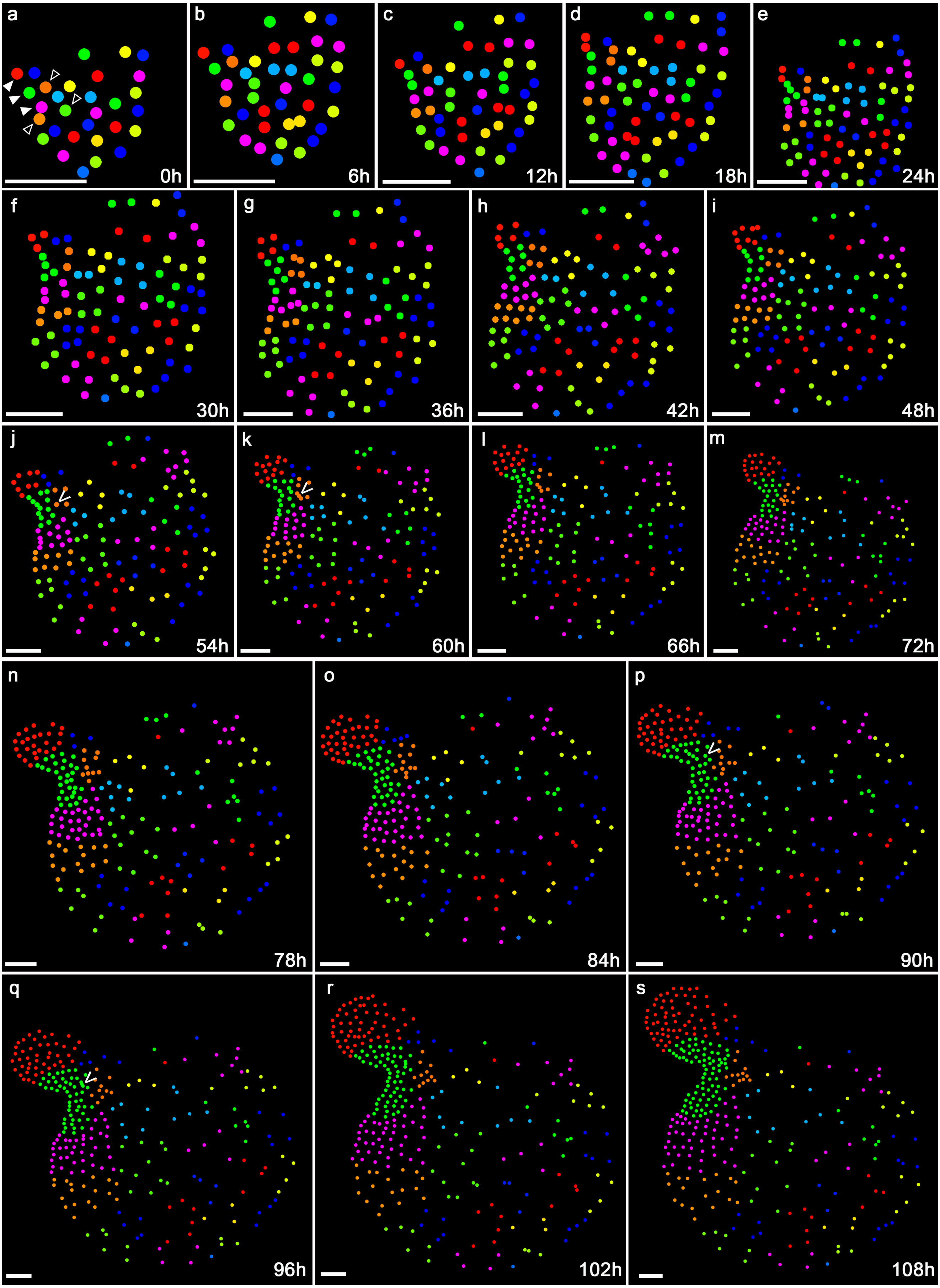

Supplementary Fig. 6 Cell lineages of the Ceratopteris gametophyte shown in Supplementary Fig. 4. (a-s) Each circle represents the location of the segmented individual nucleus shown in Supplementary Fig. 4. The cells at 0h are labeled with different colors, as the progenitors for each lineage over the 108 hours of cell division and prothallus proliferation. In the subsequent time points, the same color has been used for labeling each progenitor cell and its descendants. The solid arrowheads in (a) indicate three marginal

lineages contributing to the majority of cells in the meristem. The open arrowheads in (a) indicate the adjacent lineages in which cells showed relatively lower cell division activity than the three marginal lineages but higher division activity than the other lineages. The “V” indicates the progenitor cells of the firstly (j, k) and secondly (p, q) initiated archegonia, which belong to two different lineages. Scale bars: 50 μm.

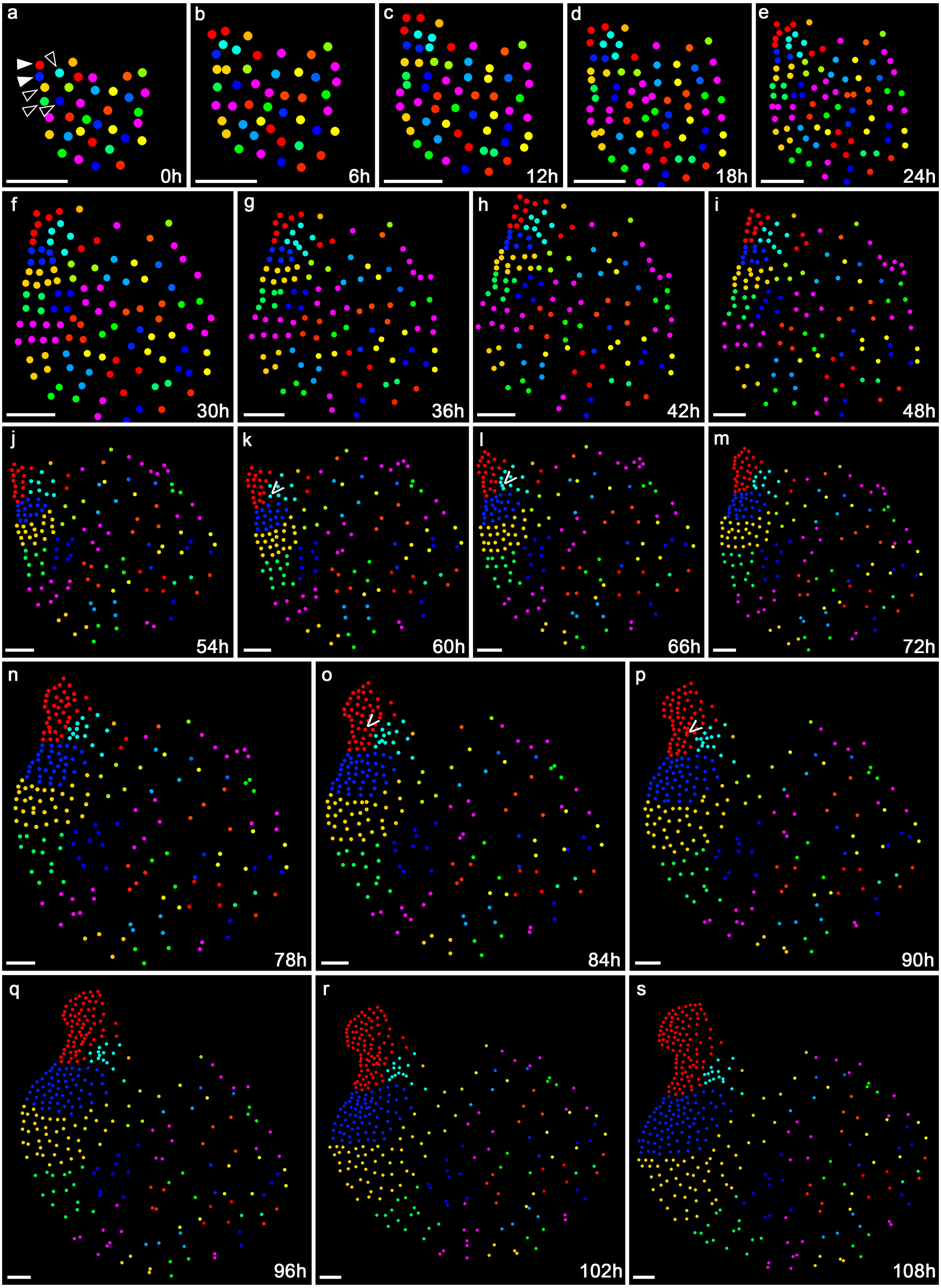

Supplementary Fig. 7 Cell lineages of the Ceratopteris gametophyte shown in Supplementary Fig. 5. (a-s) Each circle represents the location of the segmented individual nucleus shown in Supplementary Fig. 5. The cells at 0h are labeled with different colors, as the progenitors for each lineage over the 108 hours of cell division and prothallus proliferation. In the subsequent time points, the same color has been used for labeling each progenitor cell and its descendants. The solid arrowheads in (a) indicate two marginal lineages

contributing to the majority of cells of the meristem. The open arrowheads in (a) indicate the adjacent lineages in which cells showed relatively lower cell division activity than the two marginal lineages but higher division activity than the other lineages. The "V" indicates the progenitor cells of the firstly (k, l) and secondly (o, p) initiated archegonia, which belong to two different lineages. Scale bars: 50 μm.

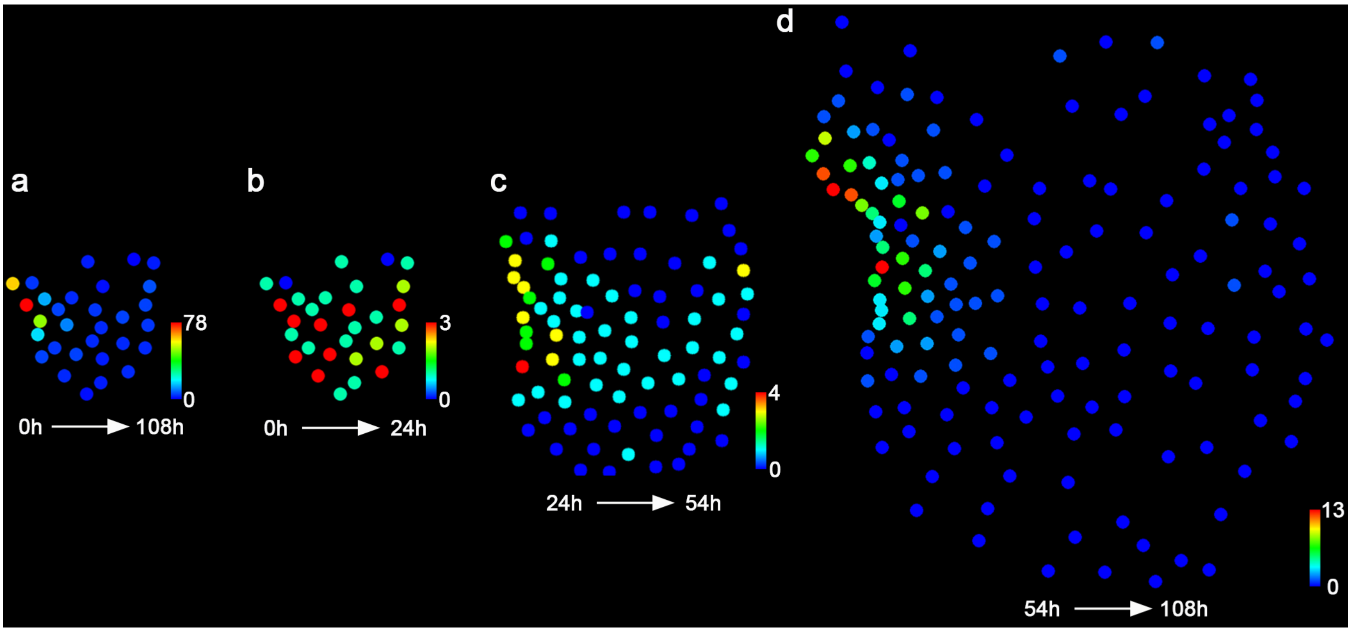

Supplementary Fig. 8 Quantification of cell division events in the Ceratopteris gametophyte shown in Supplementary Fig. 4. (a-d) Quantification of cell division events at different developmental stages, including the time frames of 0-108h (a), 0-24h (b), 24-54h (c), and 54-108h (d). Each colored dot represents the detected individual nuclei of the gametophyte shown in Supplementary Fig. 4. The quantified cell division events are mapped to the images from the first time points of the indicated intervals. Colors indicate the total number of cell division events for each cell lineage during the indicated time frames, with the scale from blue (0) to red (78) in (a); from blue (0) to red (3) in (b); from blue (0) to red (4) in (c); and from blue (0) to red (13) in (d).

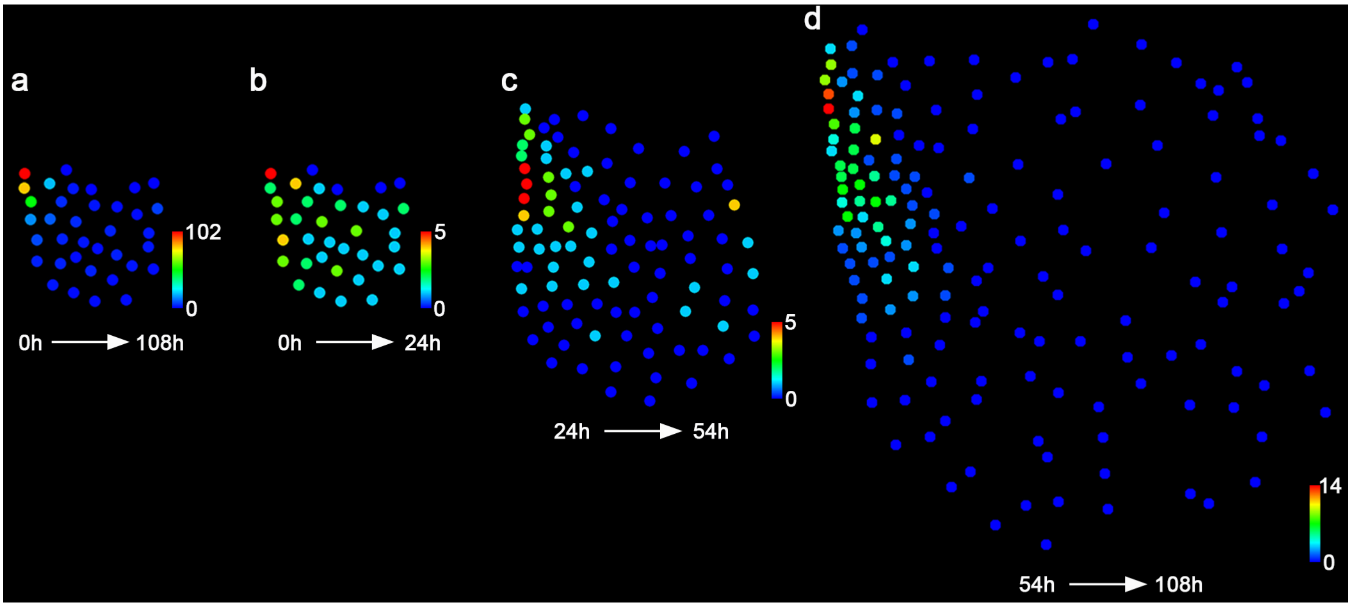

Supplementary Fig. 9 Quantification of cell division events in the Ceratopteris gametophyte shown in Supplementary Fig. 5. (a-d) Quantification of cell division events at different developmental stages, including the time frames of 0-108h (a), 0-24h (b), 24-54h (c), and 54-108h (d). Each colored dot represents the detected individual nuclei of the gametophyte shown in Supplementary Fig. 5. The quantified cell division events are mapped to the images from the first time points of the indicated intervals. Colors indicate the total number of cell division events for each cell lineage during the indicated time frames, with the scale from blue (0) to red (102) in (a); from blue (0) to red (5) in (b); from blue (0) to red (5) in (c); and from blue (0) to red (14) in (d).

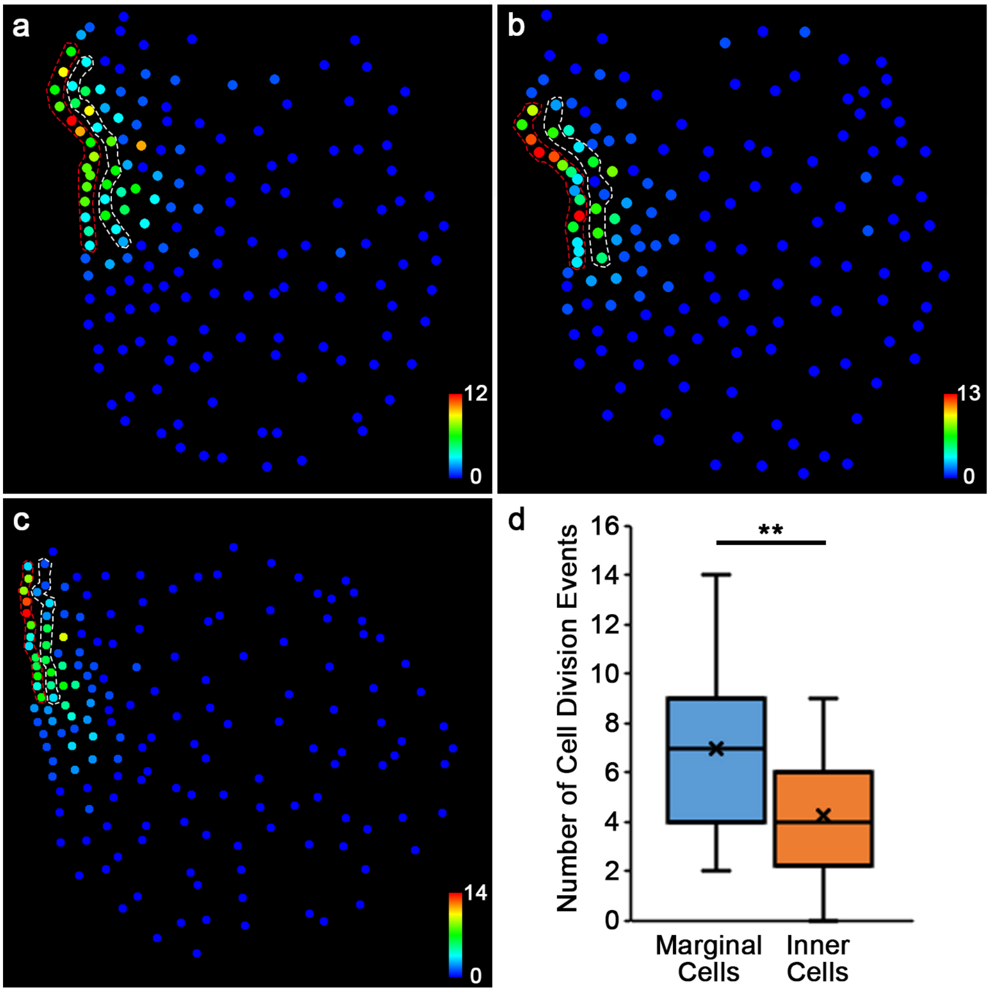

Supplementary Fig. 10 Cell division in marginal cells and their adjacent inner cells from the meristems of three gametophytes. (a-c) Quantification of cell division events within 54-108h. The quantified cell division events are mapped to the images at 54h with the scale from blue (0) to red (12) in (a); from blue (0) to red (13) in (b); from blue (0) to red (14) in (c). Marginal cells and their adjacent inner cells in a meristem at 54h are defined and highlighted with red and white dashed circles, respectively. (a) is also shown in Fig. 5d (54-108h), representing the analyzed sample shown in Fig. 2. (b) is also shown in Supplementary Fig. 8d (54-108h), representing the analyzed sample shown in Supplementary Fig. 4. (c) is also shown in Supplementary Fig. 9d (54-108h), representing the analyzed sample shown in Supplementary Fig. 5. (d) Statistical analysis of cell division events within the 54-108h time frame in the marginal cells (n = 43) and their adjacent inner cells (n = 32) from the meristems of three gametophytes. Boxplot: lower vertical bar = minimum value, lower box = lower quartile, central line = median, upper box = upper quartile, upper vertical bar = maximum value, black x = mean value. ** indicates a significant difference (p = 0.000164, < 0.001, Student's two-tailed t-test). The source data for (d) are included in Supplementary Data 5.

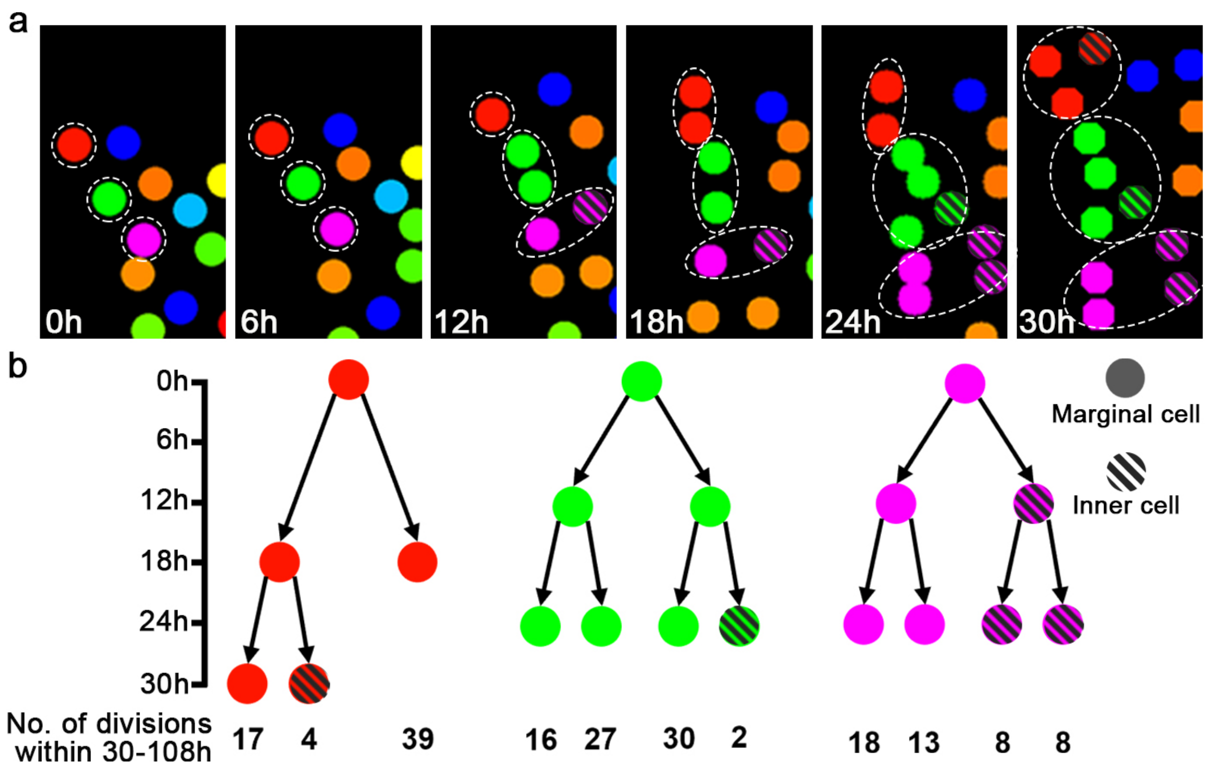

Supplementary Fig. 11 The impact of lineage and position on division activities of meristem progenitor cells in the gametophyte sample shown in Supplementary Fig. 4. (a) Cell division and lineage progression of three meristem progenitor cells from 0h to 30h. The images are part of Supplementary Fig. 6a-f (0-30h), highlighting the three cell lineages that contribute to the majority of cells in the meristem with the dashed lines and labelling marginal cells with solid circles and inner cells with the circles filled with diagonal stripes. The complete lineage progression of the three meristem progenitor cells and their descendants (the red, green and magenta sectors) is shown in Supplementary Fig. 6a-s. (b) Relationships among cell lineages, cell position and quantified division events. Each circle of one family tree represents one cell at the indicated time point from 0-30h. Two black arrows from one cell represent one round of cell division and illustrate the relationship between the cell and its two immediate progenies. Y-axis: the time-lapse from 0h to 30h. X-axis: the total number of cell division events for each cell (at 30h) and its progenies during the following 30-108h time frame. (b) labels the clonally related cells with the same color in each family tree (as red, green and magenta shown in a) and highlights the positional information of each cell (as solid circles and circles filled with diagonal stripes shown in a). During the 30-108h time frame, cells from the same progenitor cell display variable division activities, and cells located at the marginal layer (solid circles) of the meristem show more division events than the cells located at the inner (or submarginal) layer (circles filled with diagonal stripes).

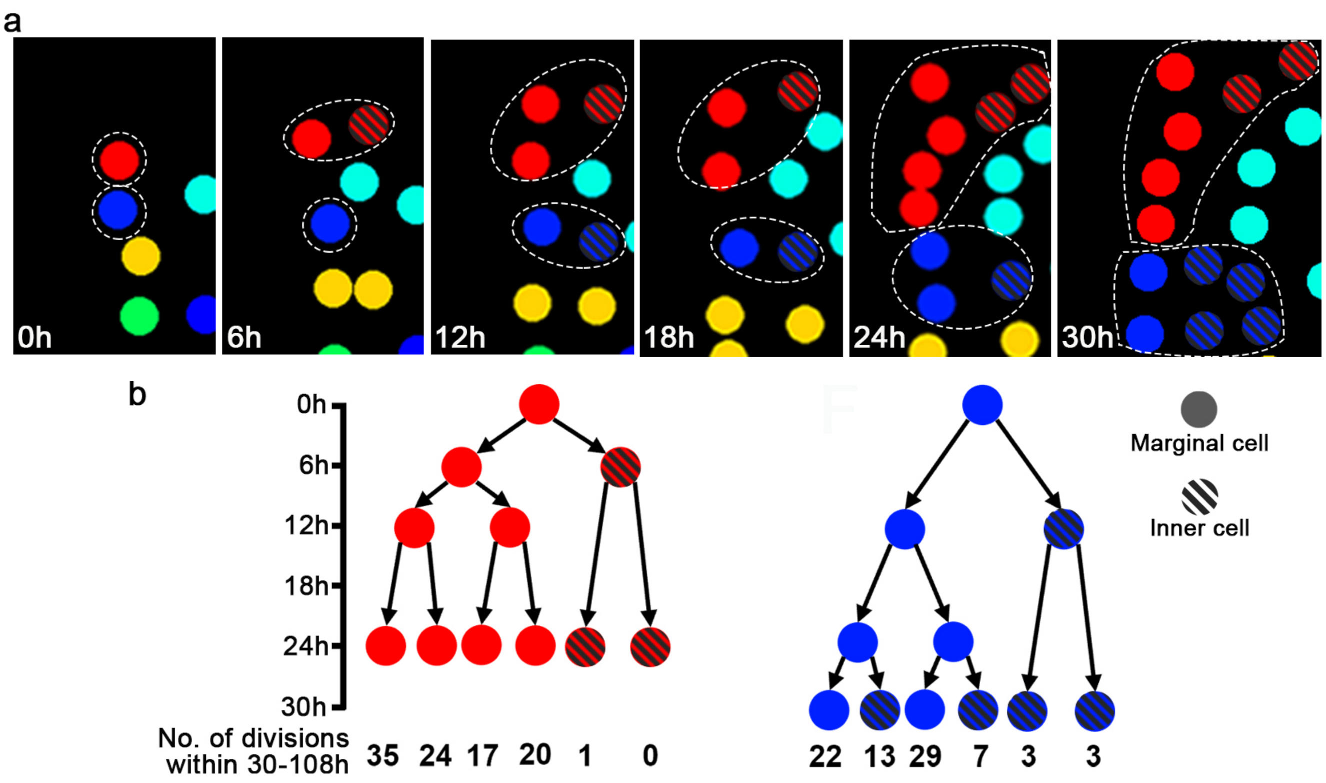

Supplementary Fig. 12 The impact of lineage and position on division activities of meristem progenitor cells in the gametophyte sample shown in Supplementary Fig. 5. (a) Cell division and lineage progression of two meristem progenitor cells from 0h to 30h. The images are part of Supplementary Fig. 7a-f (0-30h), highlighting the two cell lineages that contribute to the majority of cells in the meristem with the dashed lines and labelling marginal cells with solid circles and inner cells with the circles filled with diagonal stripes. The complete lineage progression of the two meristem progenitor cells and their descendants (the red and blue sectors) is shown in Supplementary Fig. 7a-s. (b) Relationships among cell lineages, cell position and quantified division events. Each circle of one family tree represents one cell at the indicated time point from 0-30h. Two black arrows from one cell represent one round of cell division and illustrate the relationship between the cell and its two immediate progenies. Y-axis: the time-lapse from 0h to 30h. X-axis: the total number of cell division events for each cell (at 30h) and its progenies during the following 30-108h time frame. (b) labels the clonally related cells with the same color in each family tree (as red and blue shown in a) and highlights the positional information of each cell (as solid circles and circles filled with diagonal stripes shown in a). During the 30-108h time frame, cells from the same progenitor cell display variable division activities, and cells located at the marginal layer (solid circles) of the meristem show more division events than the cells located at the inner (or submarginal) layer (circles filled with diagonal stripes).

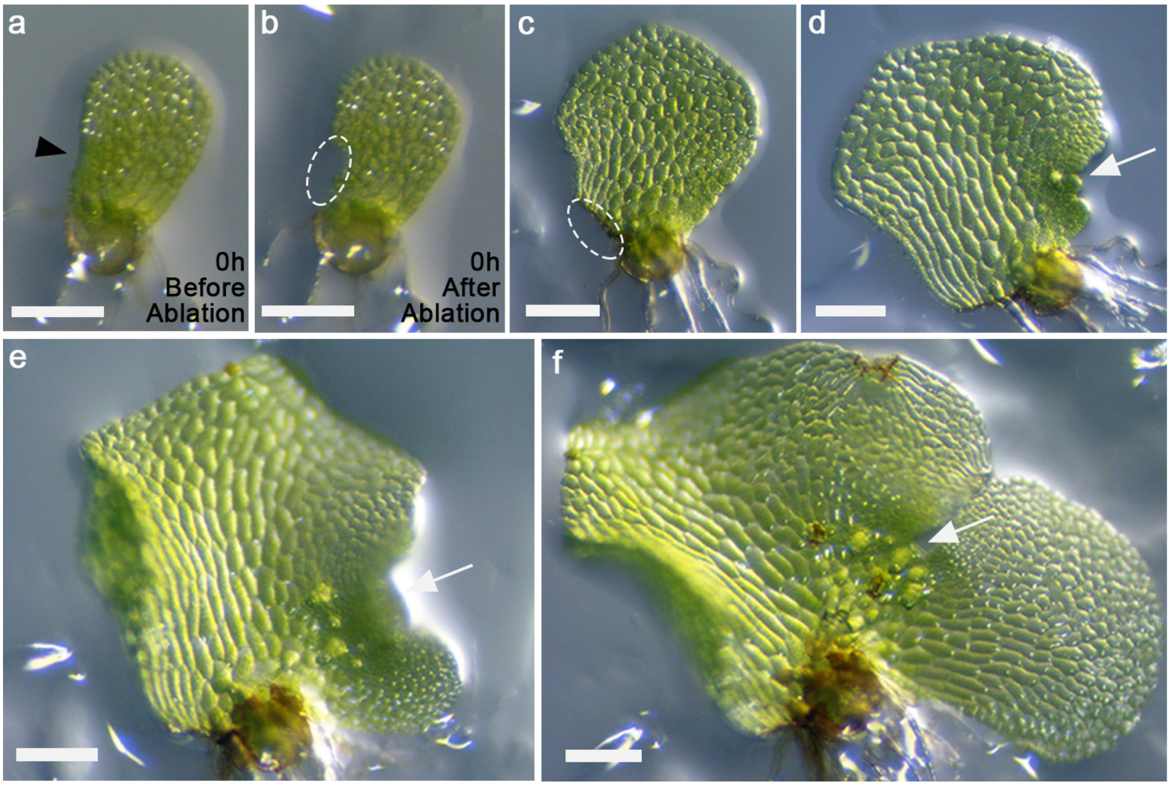

Supplementary Fig. 13 The ablation-induced *de novo* meristem formation in a wild-type hermaphroditic gametophyte. (a-f) Developmental process of one representative hermaphroditic gametophyte before (a) and after (b-f) ablating the meristem initiation site. The black arrowhead indicates the original meristem initiation site (a). The ablation area is highlighted in (b) and (c) with dashed circles. The white arrows indicate *de novo* formation of a new meristem at the region different from the original meristem initiation site (d-f). Scale bars: 0.2 mm. Five independent biological replicates were included in this experiment, and all showed *de novo* formation of new meristems and archegonia after the ablation.

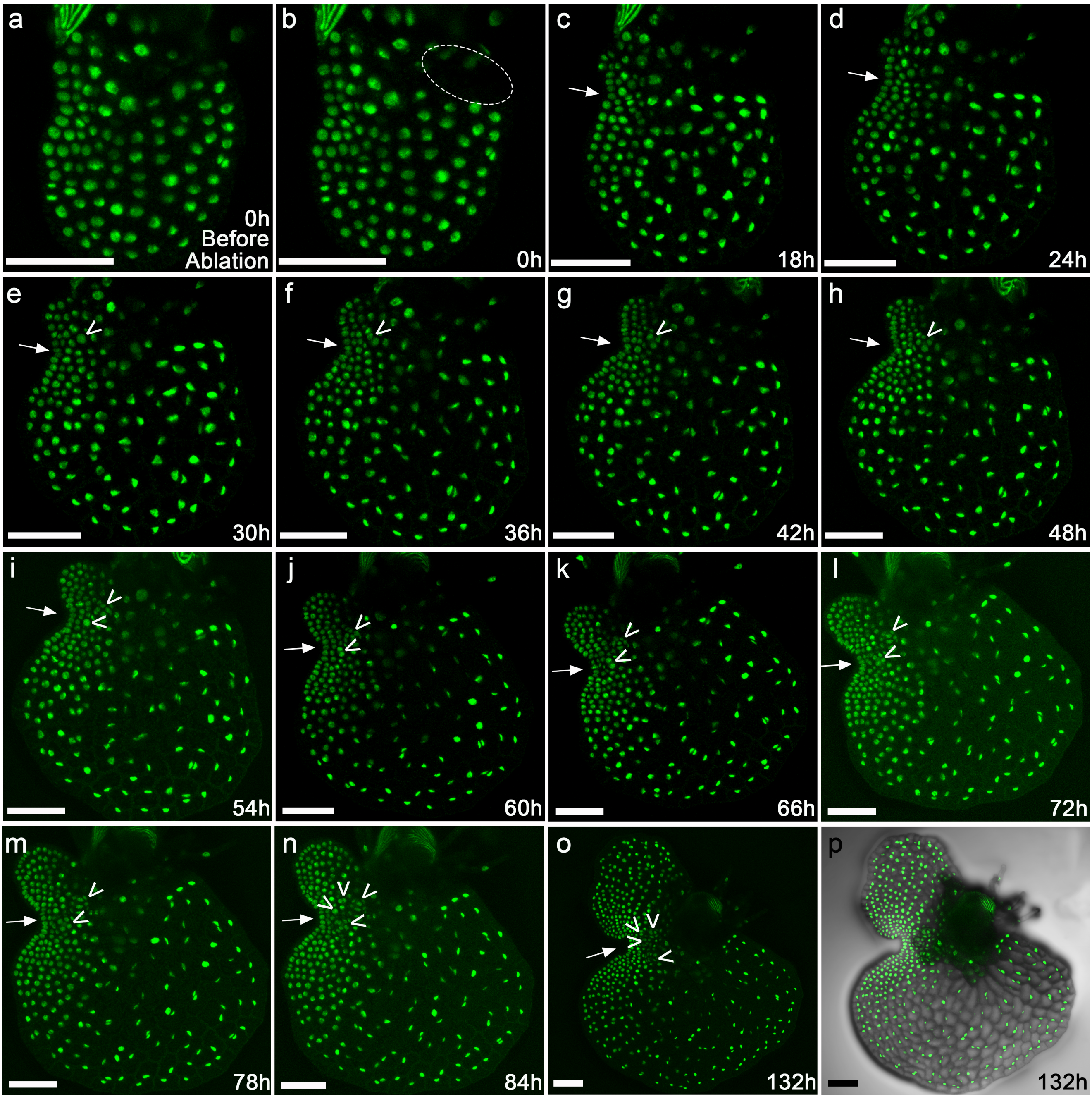

Supplementary Fig. 14 Time-lapse confocal imaging of the Ceratopteris hermaphroditic gametophyte after ablating the non-meristematic region. (a-o) Z-projection views of a hermaphroditic gametophyte expressing H2B-GFP (at 8 DAI) before (a) and after (b-o) ablating a non-meristematic area. The time-lapse imaging was performed at the 6-hour interval, from 0h to 132h. The ablated area is highlighted in (b). The white arrow indicates the continuous growth of the meristem. The "V" indicates the archegonium formed around the original meristem notch. (p) Merge of the GFP (o) and DIC (showing the cell outlines) channels. Scale bars: 100 μm. Three independent biological replicates were included in this experiment, and all showed similar developmental patterns.

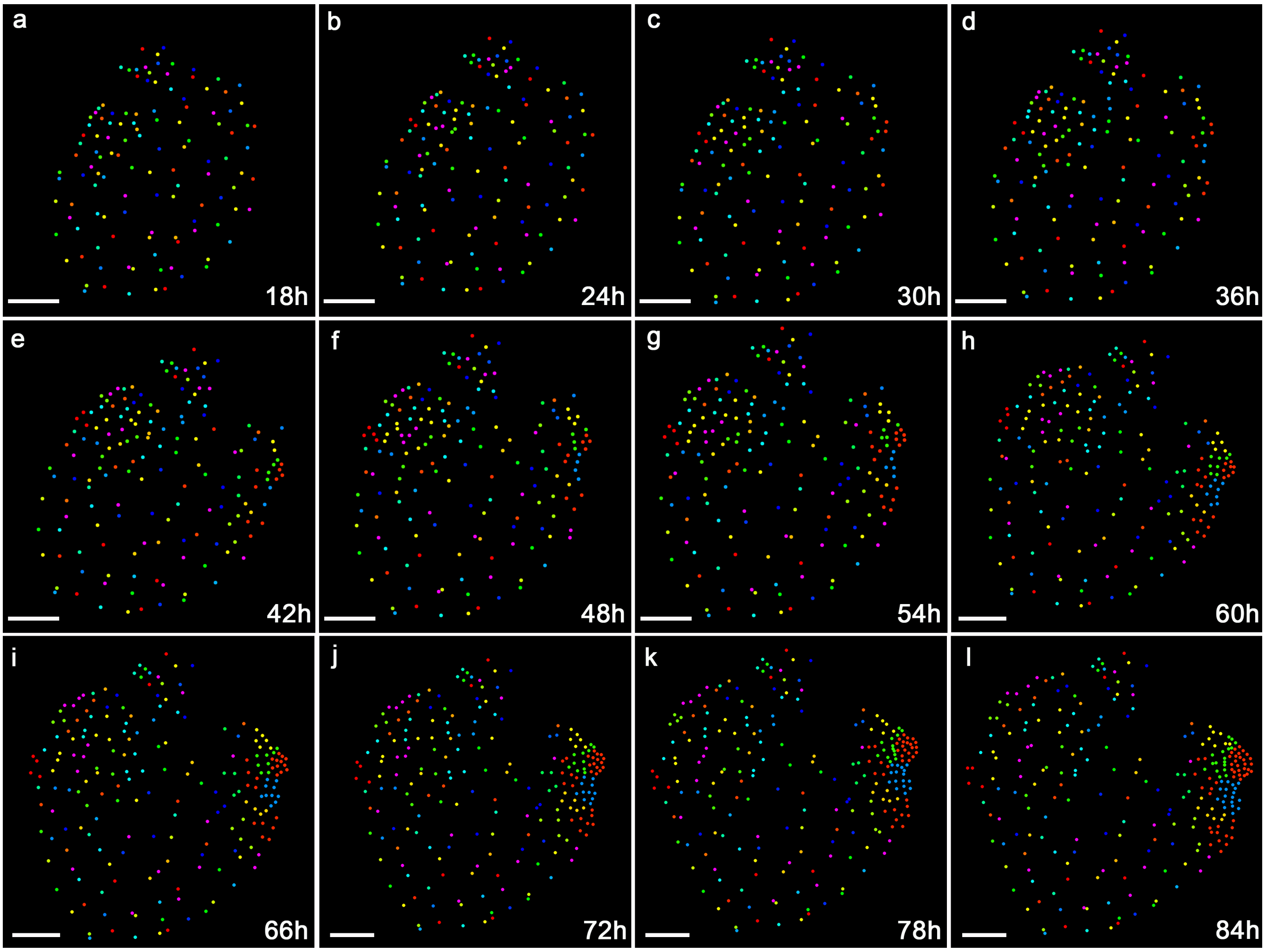

Supplementary Fig. 15 Cell lineages of the ablated Ceratopteris gametophyte. The nuclei in the confocal images from 18-84h after the ablation (Fig. 8) were segmented and detected. (a-l) Each dot represents the location of the segmented individual nucleus shown in Fig. 8. The cells present 18h after the ablation are labeled with different colors and are considered as progenitors of different lineages over the following 66 hours of cell division and new meristem formation. In the subsequent time points, the same color has been used for labeling each progenitor cell and its descendants. Scale bars: 100 µm.

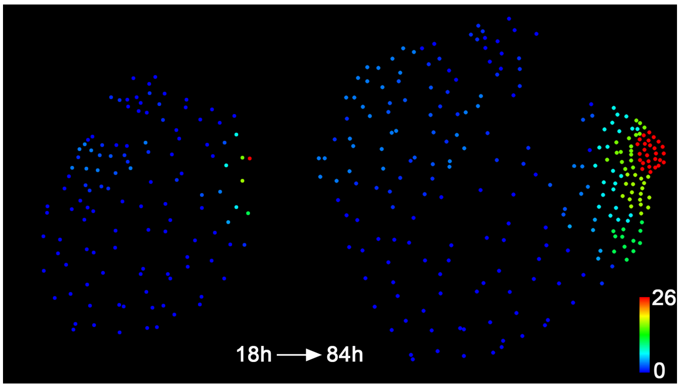

Supplementary Fig. 16 Quantification of cell division events in the ablated Ceratopteris gametophyte from 18h to 84h. Each dot represents the location of the segmented individual nucleus shown in Fig. 8. The quantified cell division events are shown in the images from the first (left panel) and last (right panel) time points of the indicated interval. Colors indicate the number of cell division events in each cell lineage with the scale from blue (0) to red (26).

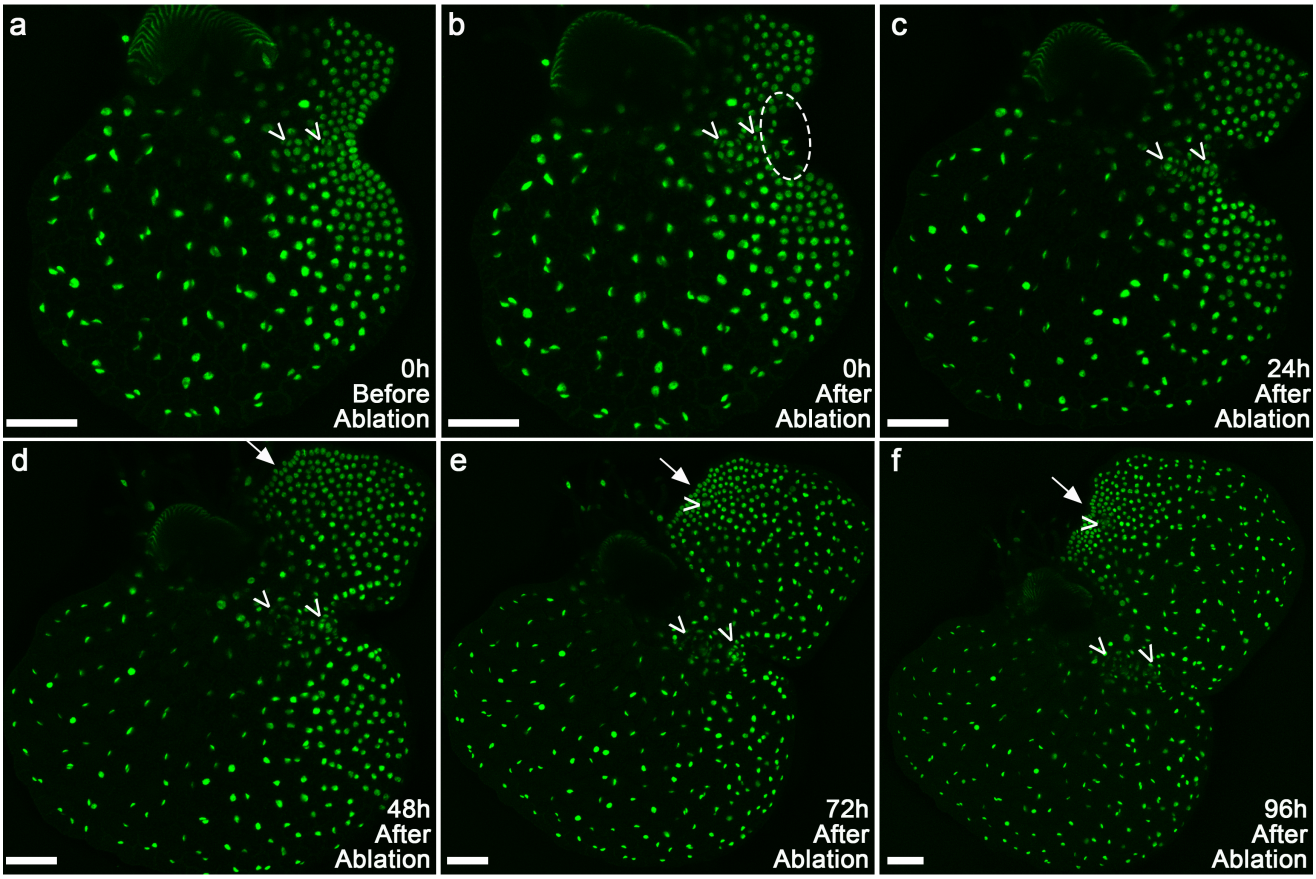

Supplementary Fig. 17 Time-lapse confocal imaging of ablation-induced new meristem formation in the gametophyte with the established meristem and determined archegonia. (a-f) The meristem in one hermaphroditic gametophyte expressing the nuclear marker H2B-GFP (at 9 DAI) was ablated. The *de novo* formation of a new meristem in response to the ablation was live-imaged by laser scanning confocal microscopy. (a-f) Z-projection views of confocal images taken at the indicated time points before (a) and after (b-f) the ablation. The ablated area is highlighted in (b). The white arrow indicates the newly initiated meristem after the ablation. The "V" indicates the archegonium formed adjacent to either the original meristem or the newly initiated meristem. Scale bars: 100 µm. Three independent biological replicates were included in this experiment, and all showed similar developmental patterns.

Supplementary Fig. 18 Nucleotide sequence of the *pCrHAM::H2B-GFP::3'CrHAM* expression cassette. The 5' *CrHAM* promoter sequence is labeled in blue. The *H2B-GFP* coding sequence is labeled in green. The 3' *CrHAM* terminator sequence is labeled in purple. All the restriction recognition sites are in lower case.

gcggccgcGTTGTTTGGATACTTAGGTGGAGGTTGTGTGTTCAATGCTTGCGGGCTATTGAACATGCT
TGAGCCAGTGTGGCCAATCTACCCTTGCAATGCCTTCGGGCCATTGAAACTATTTGTCTTCAAGT
AGCCTTTTCCTTGTGATGCTTTGACAGTCTAACTCTTGCTTTATGAGATATGGAGGATCTCATGAT
AACGATGCCCATACAATTGTTTTTTCAATATGTTTAAGCCCAAAGAAATTAAACTTCAACTGAA
CTTCTAAAAAAAAATTATATCTCCTAAAGATATGTTTTCACCTAATGTATACACTTGTGCATAAGCA
TGCATGTATGAGGCATGTATGCATGCATACATACATCTCTCACACATGTTCATGTGTGAGATGTATA
CACTTGTGCATAAGCATGCATGTATGAGGCATGTATGCATGCATACATACATCTCTCACACATGTT
CATGTGTGAGGCATGTATGCATGCATACATACATCTCTCTTACACATGTTCATGGTTCACCCATTCT
TTTCCTATCCGGAGACGTGCTCCAACTCTCCAATGTGAACCCAACCTAATACATCACACACGTTT
GTCCAGAGAGACATACAATATGTCGCAGACATCTTCATTCTCCCCCCACTTTTGTGCACTCTTCTT
TTCTCCCTTTGTGCGGCAGCAATTGGGGGGGCACTTTTAGAAAGCAAAGTGATGGCTATGTGAC
TCGAGAAAAGGCTTCCTCGTAGCAGGGCGGGAACGCAGGCCAAGAAAGAGGGTGACGTCATC
GAGTCGCTCCCTCAAGGCCCCACGGCATTGTCCCAATGAAGGGCGCGCCTTTCAATGCGACAGA
GAAAGAGCCTGAGCGAGACGCAGACAAGGAGAGACAGTGGAGAGTCCAGAGTCCACACGCGA
GGCTCCATGGTCCATGGGGCGGGGCCGCAATCGGGGACGTGAGAACCGCCGCTTAACCGGCCA
CGTCAGTCCGTCAACATGAGCGGTAAAAGCGCTTCCTCCTCCCATCATCCGTCGTATGGAACCCA
CAGCGCCTCTGCACTGACACCGCTGCTGCTACGACCGCCCGCTGATTGGCCATTCCTTCTCAGA
GCTGGAGCAAGTCAGTCATGCAGGTCACCGTCATCGGGCCTGCTGCTCCTCTACAGTTTCTCACT
GCTGCACGGTGCACCTCCTCTTCCGCCCGGCTGCCCGCCCGCCAGGCGCGCGCTTGTCTCTCGT
GTCCTCATGCGCTGCCAAGCGCGCGTGTGCAGTTCGCCGAAGCCGGAGGACAAGTTTGGCGTC
GTTAAAATTGGGGTCTTTCGCTATCTATAAGCCACCAAATCAAGAGGTGAACGCCAAATTGGCGG
TCCTCTATTATCTCATCGGACGAAGAAACTGTGTTCCTCTTGGCTAAGAAGAAAAGAAAAATTGG
CGTGAAAAGAGTAAGAAGGAAAAAAAATTCTGCAGCTCTCCAGAGGCTTTTTTGCTTTTCGATG
TTGTATGTTCACACGGTCACCGGGCAGCGGCCAGTCGTCCGCCCATACACCCCCCCTCGCGGAT
CTAAGGATTGGTGGTGCAGTACCTTCACGATTGGTTCATGTAATCCGACAGGCAGCCCTCCCGAG
CGGTCTCGCGGTAATCCGTCCCCCCGAGGACTCGCATCGCATCCTCCCGTTTAGTGCTTCCCCTC
TCTCTCTCTGGCTCTCTCCCCTGAGTTCCCTCCCTGCAAGCTTCCATGTCCAAGCAGTGAACACA
GAAGCTGCCACACGCGCGTAGCCAAGCGCACAGACCCACCCCCGCATAGTTTCTCACAGATAGG
AGGGCAGGCAATTCTGTCGAGCCCCTCCAATATTTTCTGCACATCACTAAAACCAAAATGCATTG
TCCCTGTCCGCTCTAACCCCATGCCGGCCCACTCACTGCCCTGTCCGTCTCCTTCTGAATCCTCTC
CTCCCCCTCCGCGGTCCAAAAGCCCTCCTTTACCTTCTCATTTGTCCTGTTCTAGCCTCCGTCTTG
CAACAATGGCGTCCTCAGCAGGCTTCACTCCTACCCATCCATCATCCGCATTCGCATCCCTCGATC
CTGTTGCTGAACTTTCATTACTCTGCTAATCTGCTGCCTCATGGTCCAATCCCGCCGCGTCTAATG
CTTTGTCTCCGGAATTTCGCCTTCTTCCTTTCTGCAAATGCGAGATCTGCTGCTGCGTCCACTAAT
CTTCATTCCACTTTACGGTGTGCATTCGAGCGCGAGTCGTAGCTTTGCATTTTGGGTTTGATCCTT
TTGAgcggccgcCCCCTTCACCGGATCCACAATGGCGAAGGCAGATAAGAAACCAGCGGAGAAGA
AACCGGCAGAGAAAACTCCGGCAGTCGAACCAGCAGCAGCGGCAGAGAAGAAACCAAAAGCC
GGAAAGAAACTCCCCAAGGAACCAGCCGTCGCCGGAGACAAGAAGAAGAAGAGATCAAAGAA
GAACGTTGAGACATACAAGATCTACATCTTCAAGGTGTTGAAGCAAGTTCATCCAGACATCGGA
ATCTCAAGCAAAGCCATGGGAATCATGAACAGTTTCATCAACGATATCTTTGAGAAGCTTGCTGG
TGAGTCTTCGAAGCTTGCGAGGTACAACAAGAAGCCGACGATTACTTCCAGGGAGATTCAGACT
GCGGTTAGACTTGTGTTGCCCGGTGAGTTGGCGAAACATGCTGTGTCTGAAGGGACTAAGGCGG
TTACGAAGTTTACGAGTTCTCCTGCTGCGGCTGCTGCCGCTGCGGCAGCCCGATCCATGGCTGC
AGCCAAGGGCGAGGAGCTGTTCACCGGGGTGGTGCCCATCCTGGTCGAGCTGGACGGCGACGT

AACGGCCACAAGTTCAGCGTGTCCGGCGAGGGCGAGGGCGATGCCACCTACGGCAAGCTGAC
CCTGAAGTTCATCTGCACCACCGGCAAGCTGCCCGTGCCCTGGCCCACCCTCGTGACCACCCTG
ACCTACGGCGTGCAGTGCTTCAGCCGCTACCCCGACCACATGAAGCAGCACGACTTCTTCAAGT
CCGCCATGCCCGAAGGCTACGTCCAGGAGCGCACCATCTTCTTCAAGGACGACGGCAACTACAA
GACCCGCGCCGAGGTGAAGTTCGAGGGCGACACCCTGGTGAACCGCATCGAGCTGAAGGGCAT
CGACTTCAAGGAGGACGGCAACATCCTGGGGCACAAGCTGGAGTACAACTACAACAGCCACAA
CGTCTATATCATGGCCGACAAGCAGAAGAACGGCATCAAGGTGAACTTCAAGATCCGCCACAAC
ATCGAGGACGGCAGCGTGCAGCTCGCCGACCACTACCAGCAGAACACCCCCATCGGCGACGGC
CCCGTGCTGCTGCCCGACAACCACTACCTGAGCACCCAGTCCAAACTGAGCAAAGACCCCAAC
GAGAAGCGCGATCACATGGTCCTGCTGGAGTTCGTGACCGCCGCCGGGATCACTCTCGGCATGG
ACGAGCTGTACAAGTAAGAGCTCCCAAGGGTGggcgcgccAAGGTAGTTGATATAAGACGTTTATTC
CGGGCCCTTCTTACTACTTCACCCTCAGCACCGTCCTCACCACTACAGCTACCACCATCATAGCC
TTCAACCCCGTGTTCTCTATAATCTCACTTCCCTTTACCTCTTTCATCCTCTTACAGCTTCTTTTGT
CATGTGCTTTCTTTTCTGTCTCCTCGTATATGCTTTCCTGGTATACTCACGTGTAGCTCATGTAGCT
GCAGCACGAGCACAACTCCCCTACATCGTCATCATAGTCAGAGGCAATCTTTATCTGCCTCTTTG
TTATTTTGGTTAATGTGCAGGATTCTGATTCCACCTTGGTTCAGCCTATATGCTGGATGTTTGGGG
TCACAGTTGTTGAAAGTGCAATAATCTGTCACTGGACTTGTGCTTCCATTCTTATAATTAATCTTG
TGCATTGTTTCAAATGGATTTAGTTTATTCTTTATGACTTGAGCTGATGTGAAAAAAGGCAGTGA
TTAGAGTGGGTTGCTGAATATTTCATTCCCCAGTGAAGGTCAGATGAATGGAATGTGTTTCAGTG
AATATCTATCCCACATCTACAGGTGCAGACTTGGATAATGCATTTATCTGATGGTAGATTGTGACT
GGCTACCGGTAACTCTACATGATGTGCAATGGTCAGAAGGCCGTTTGTCAAAGAATGTCTCATAT
TTAGAGACTGTAGTAGACCTTGGCATGGCTGACTAACCTTCAAATCTGATGCGGTATTTCTTGTAT
TCATGTCTGCATTTGTTGAGGACACTTGTCTCATAGAGAATGTGCTGATGTAGTGGGGGCTGGGA
ATGGCAGCATAGGCATCATAATTGTTGATTTGGTTGTGGCTAAGCTCTTTATATCTTGAGAATTGT
GGTTCCAGCTCACTTCATACAAAGGATATTTTGGGACTACACATTTTAGATGCTTTTCCACTATCT
TATATGGCTTGCAGTTAACAACTCCAAATGGTGATGGTAAGGGTAGATATTAAGTTCCGAGTAGC
Aggcgcgcc

Supplementary Fig. 19 Segmented nuclei from the images shown in Fig. 2. Each nucleus in the confocal images from 0-108h was segmented and labelled with the unique ID.

0h

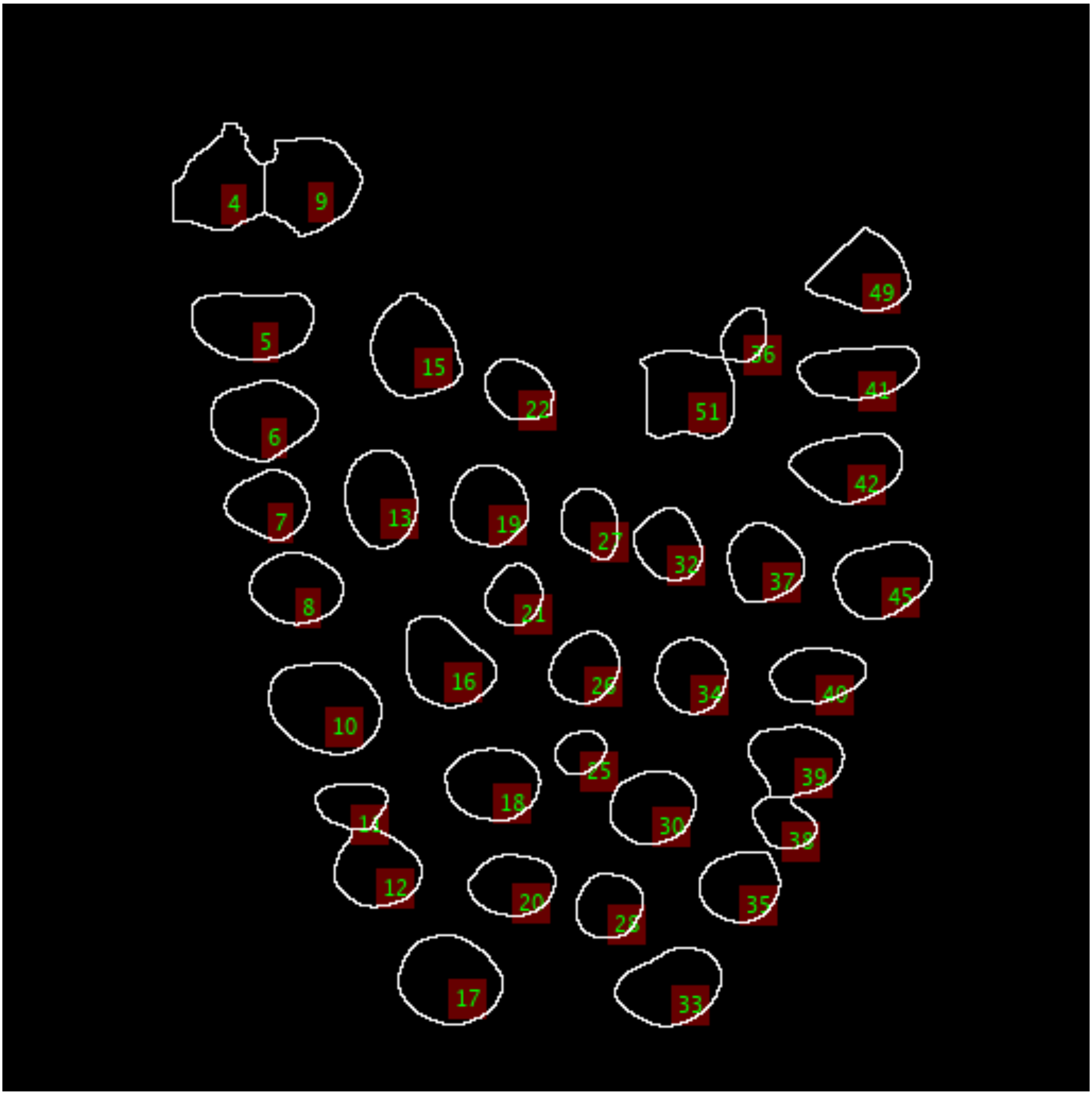

6h

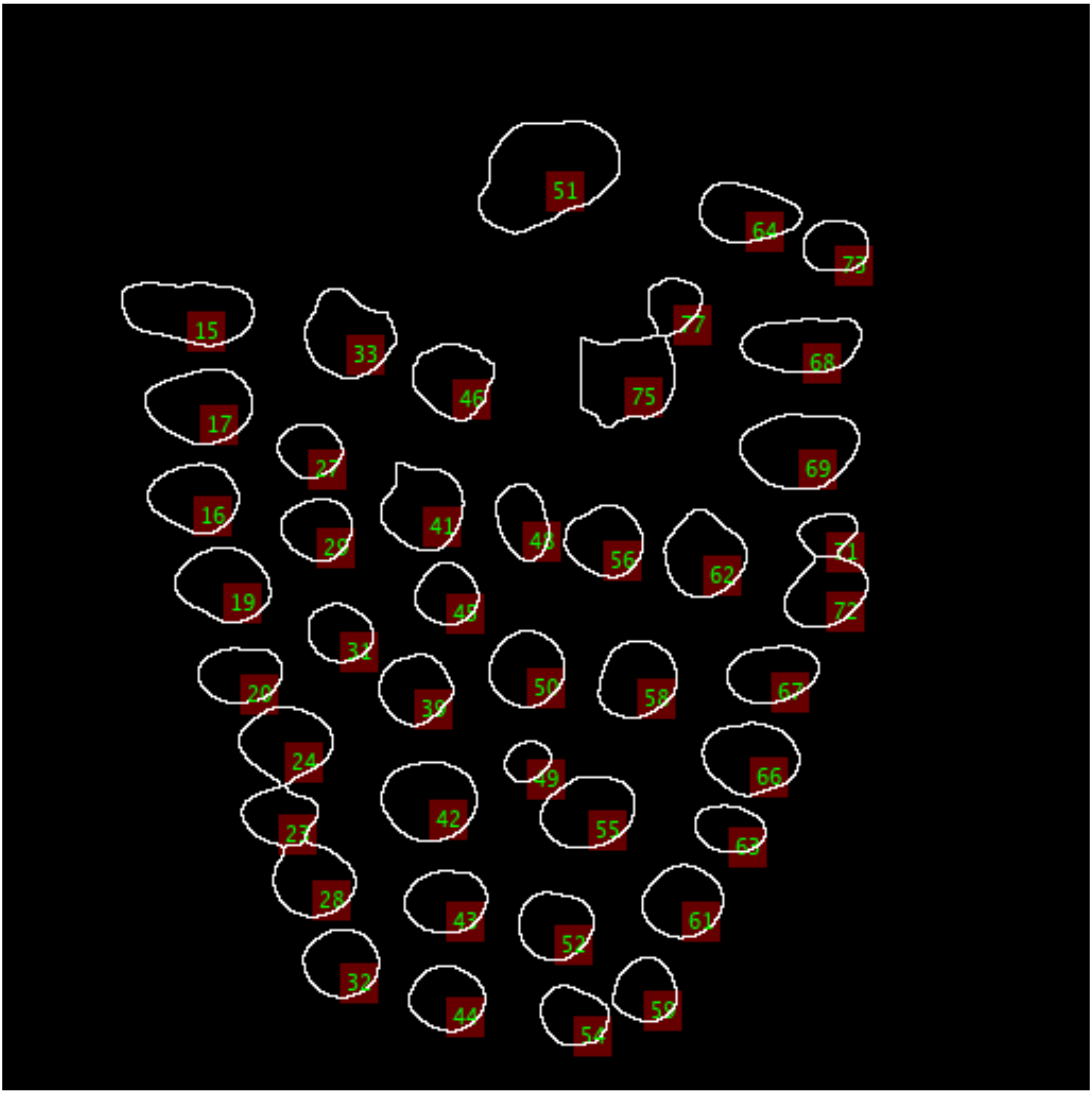

51
64
73
15
33
77
68
46
75
17
27
69
16
41
48
56
62
71
29
19
45
72
31
20
50
58
67
39
24
49
66
42
55
23
63
28
61
43
52
32
59
44
54

12h

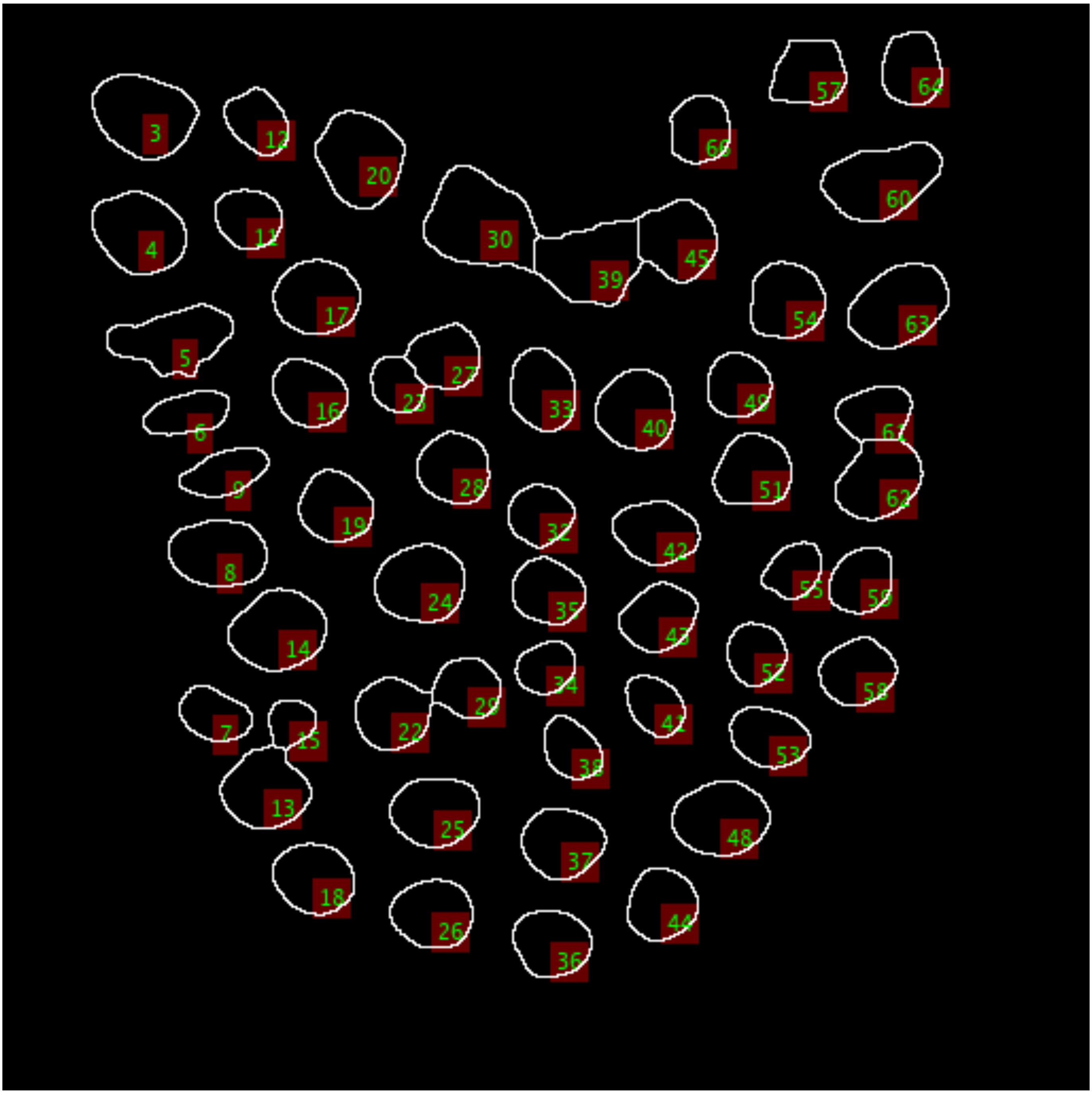

57
64
3
12
20
60
30
4
11
45
39
17
54
63
5
27
33
40
49
16
6
28
51
62
9
19
32
42
8
24
35
55
14
43
52
58
34
7
15
22
29
41
38
53
13
25
48
37
18
44
26
36

18h

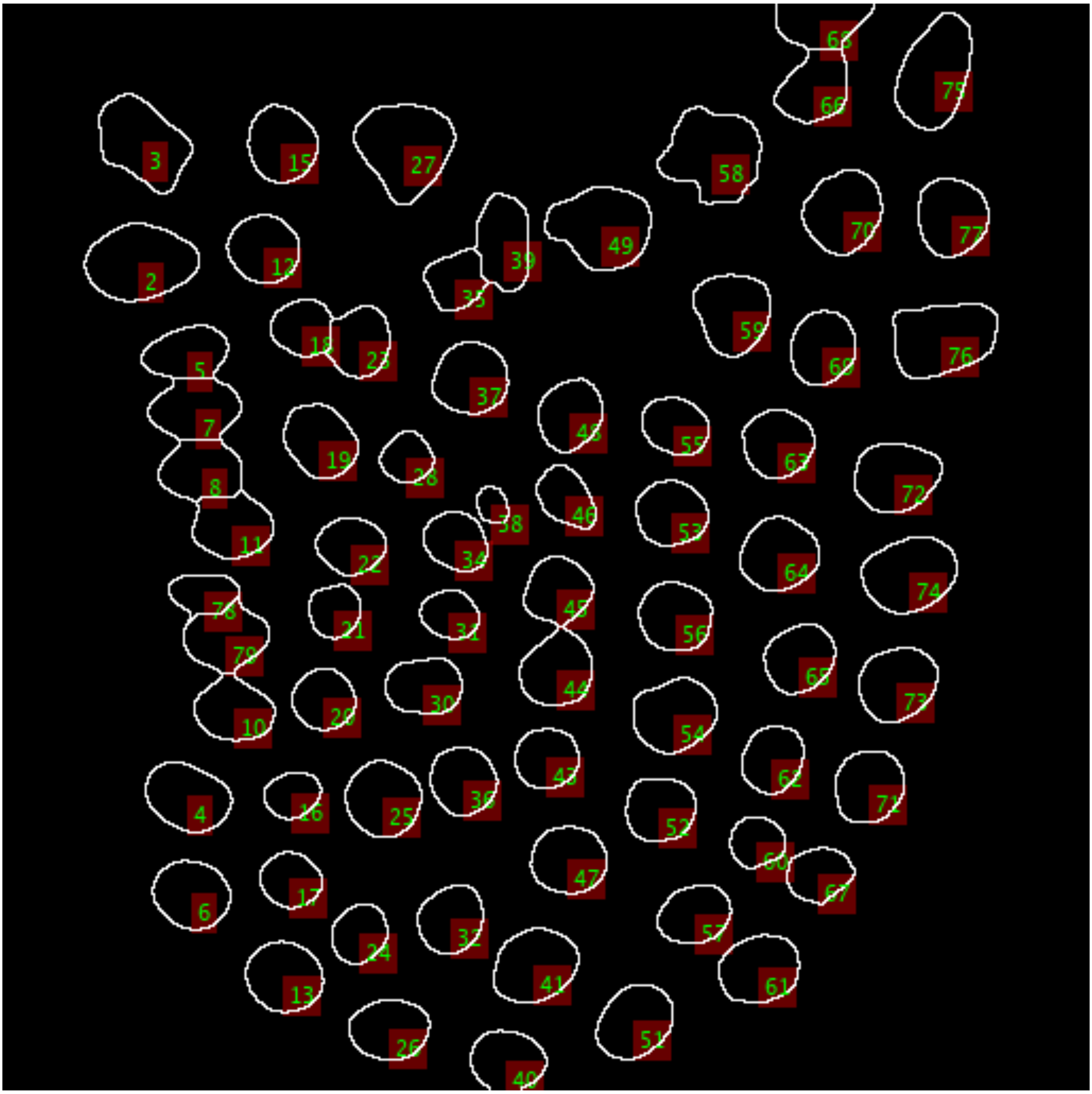

24h

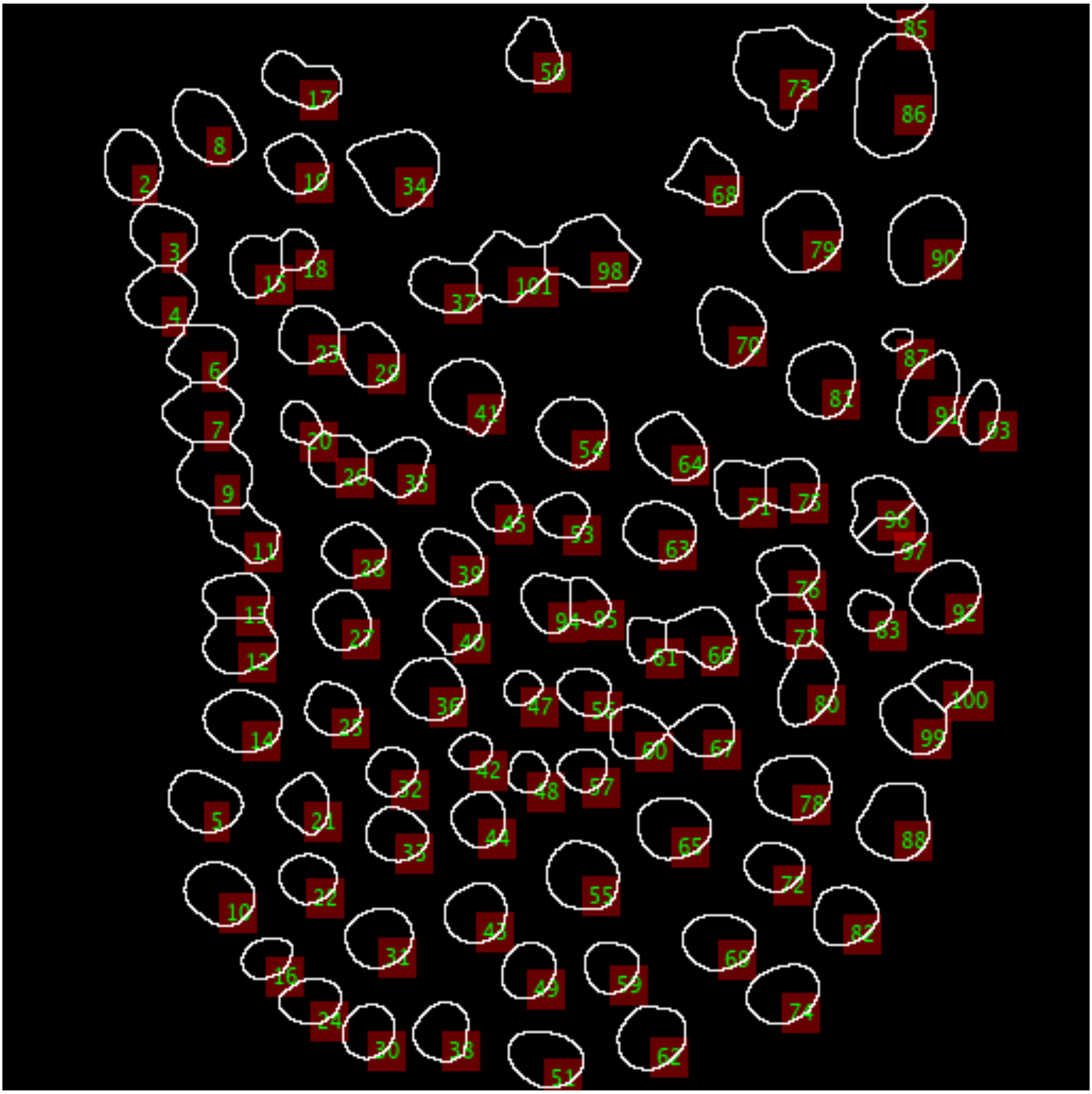

30h

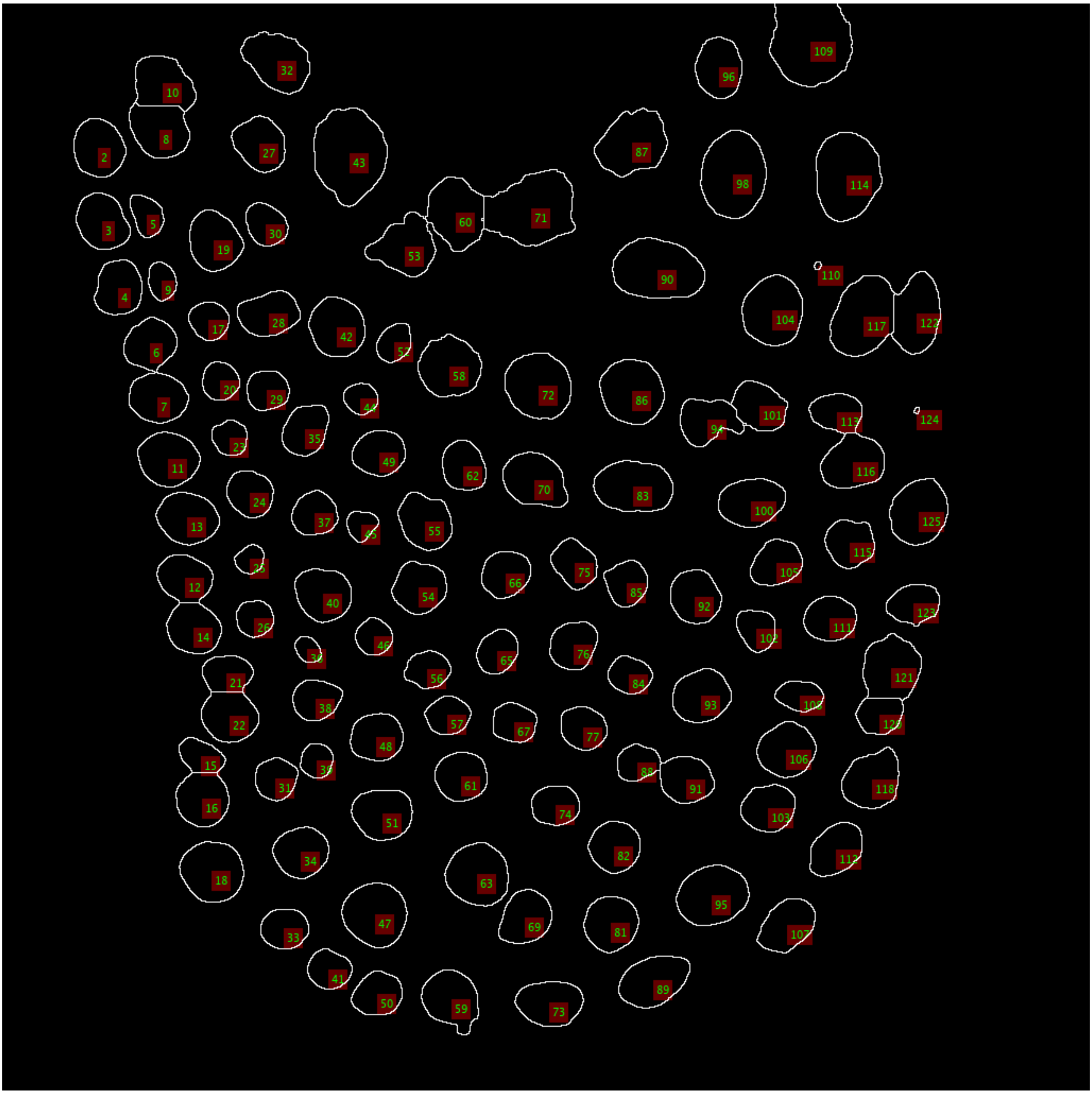

109
96
32
10
87
8
27
43
2
98
114
60
71
3
5
30
19
53
90
110
4
9
104
117
122
28
17
42
52
6
58
72
86
20
29
44
101
112
124
7
35
94
23
49
11
62
70
83
116
24
100
37
45
55
13
125
115
25
75
105
66
12
85
40
54
92
123
14
26
111
102
46
39
76
65
56
121
21
84
38
93
108
22
57
67
77
120
48
15
88
106
36
31
61
91
118
16
74
103
51
82
34
113
18
63
95
69
81
47
33
107
41
89
50
59
73

36h

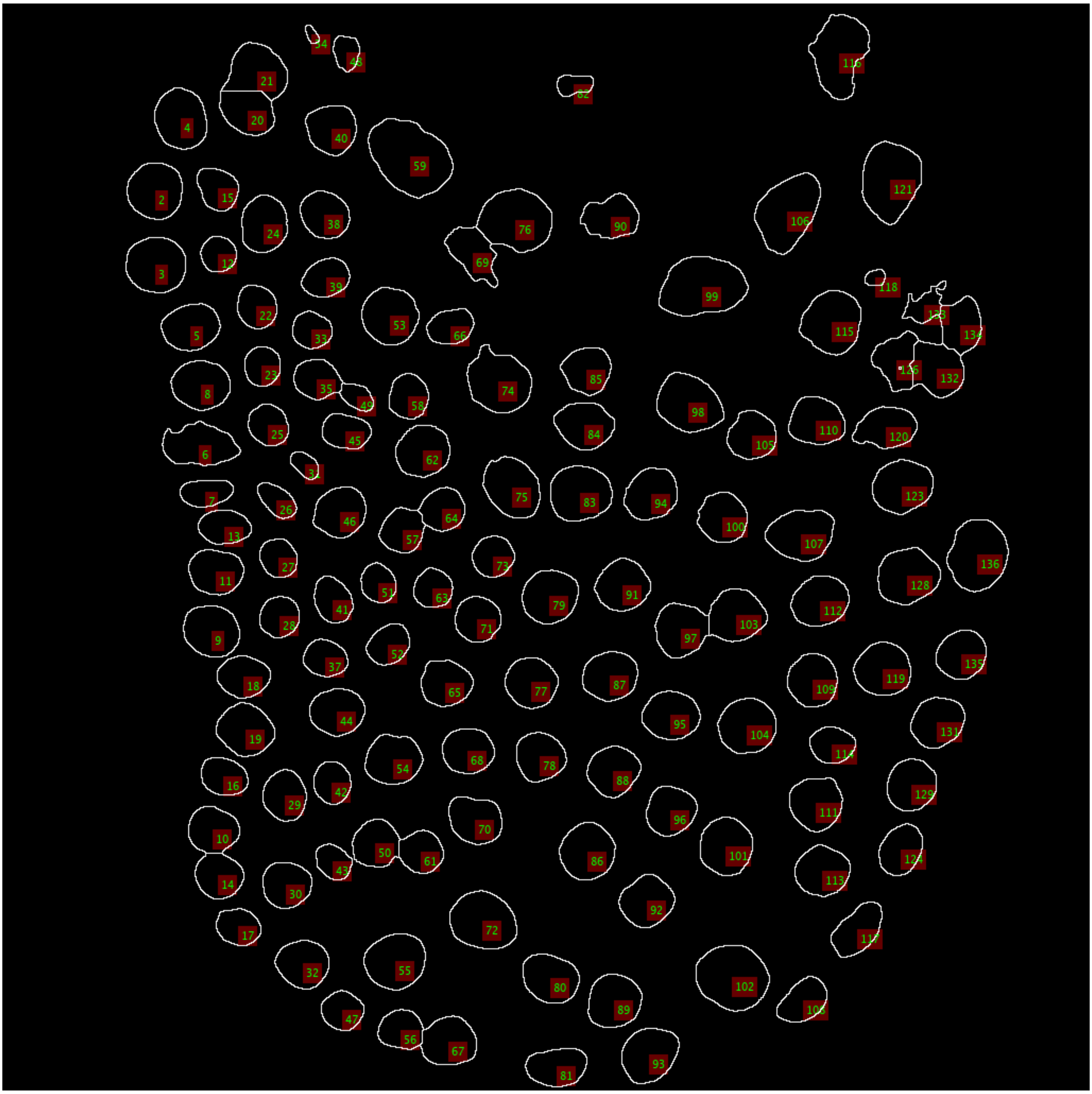

42h

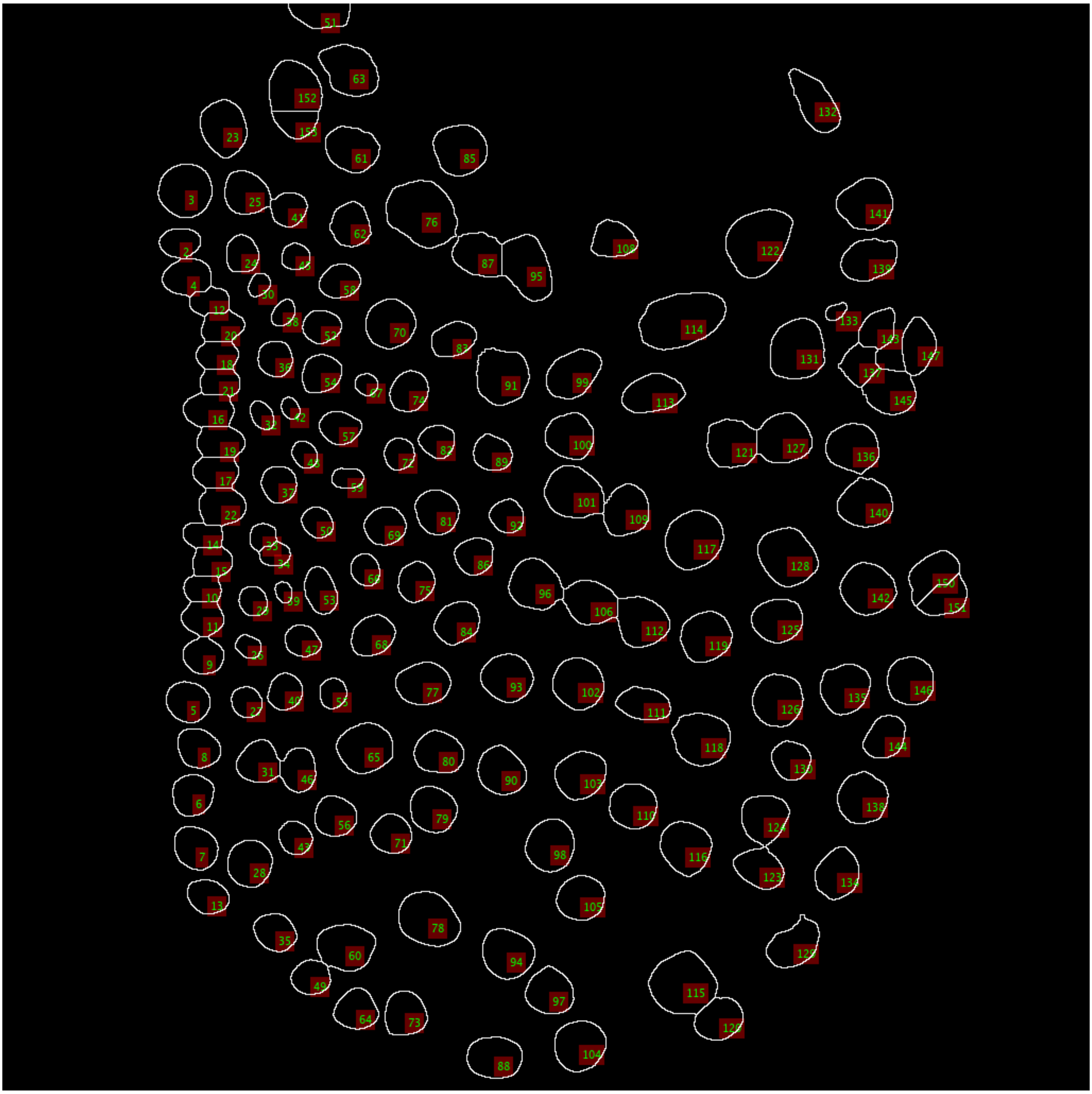

48h

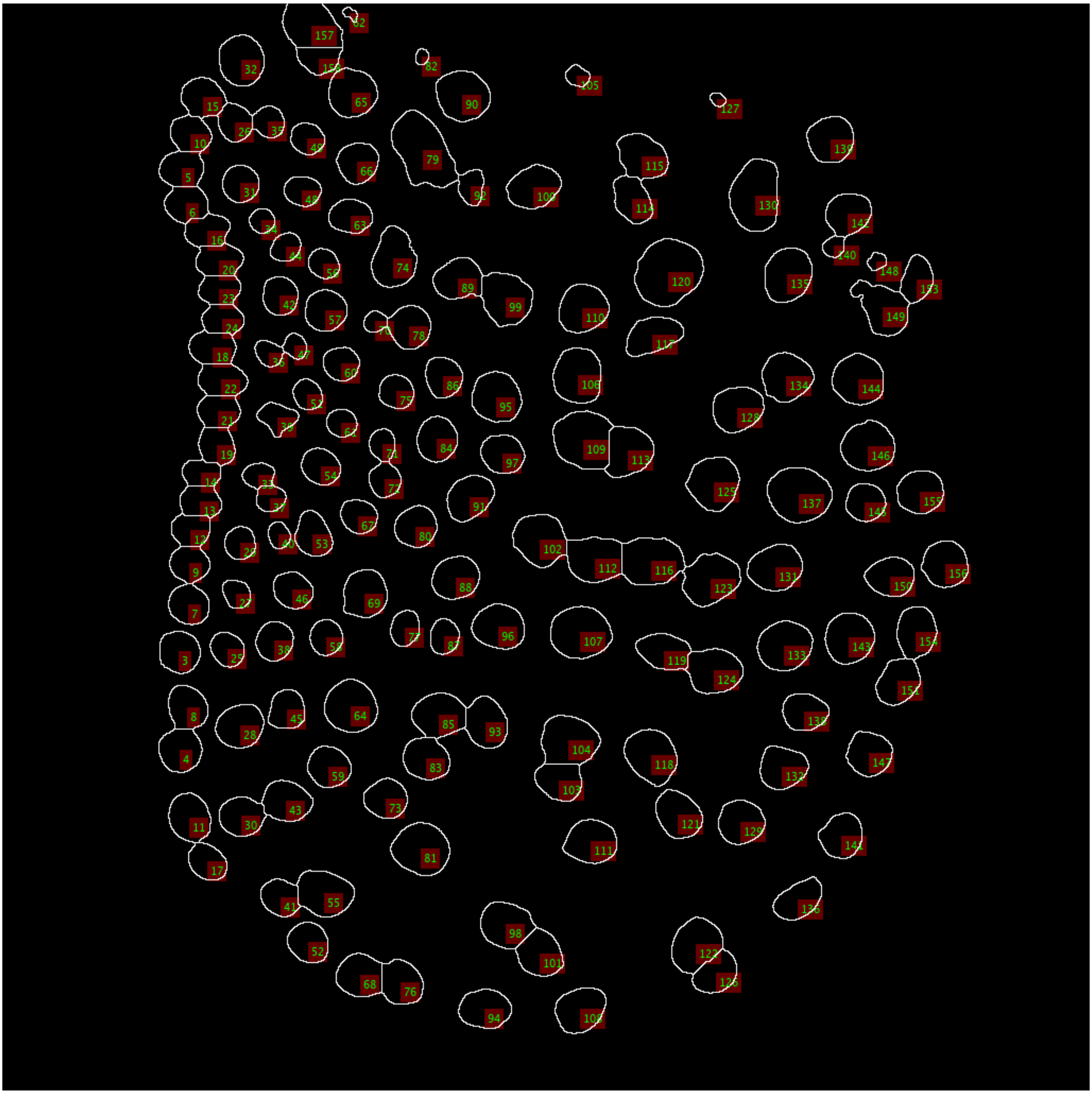

54h

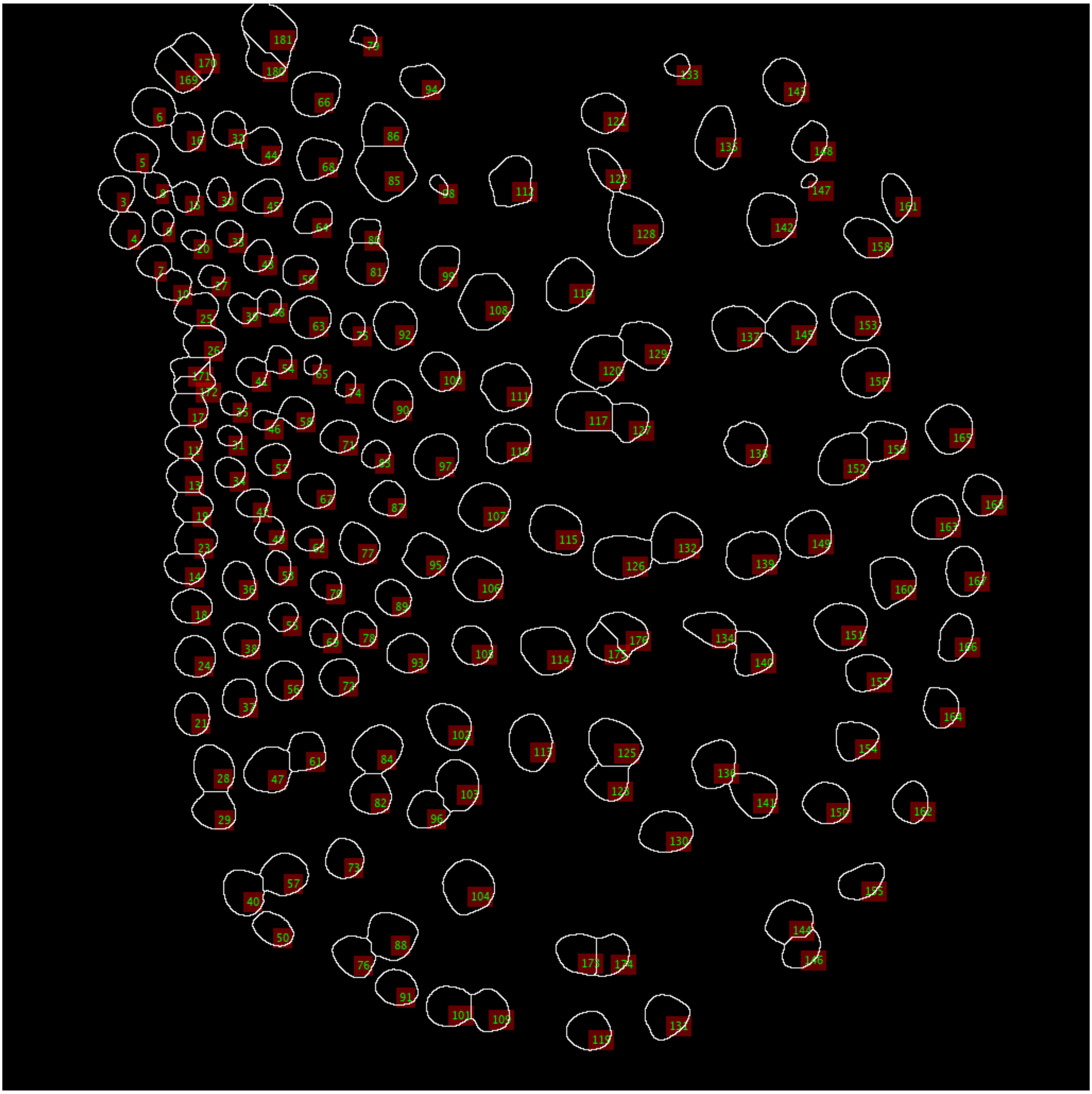

60h

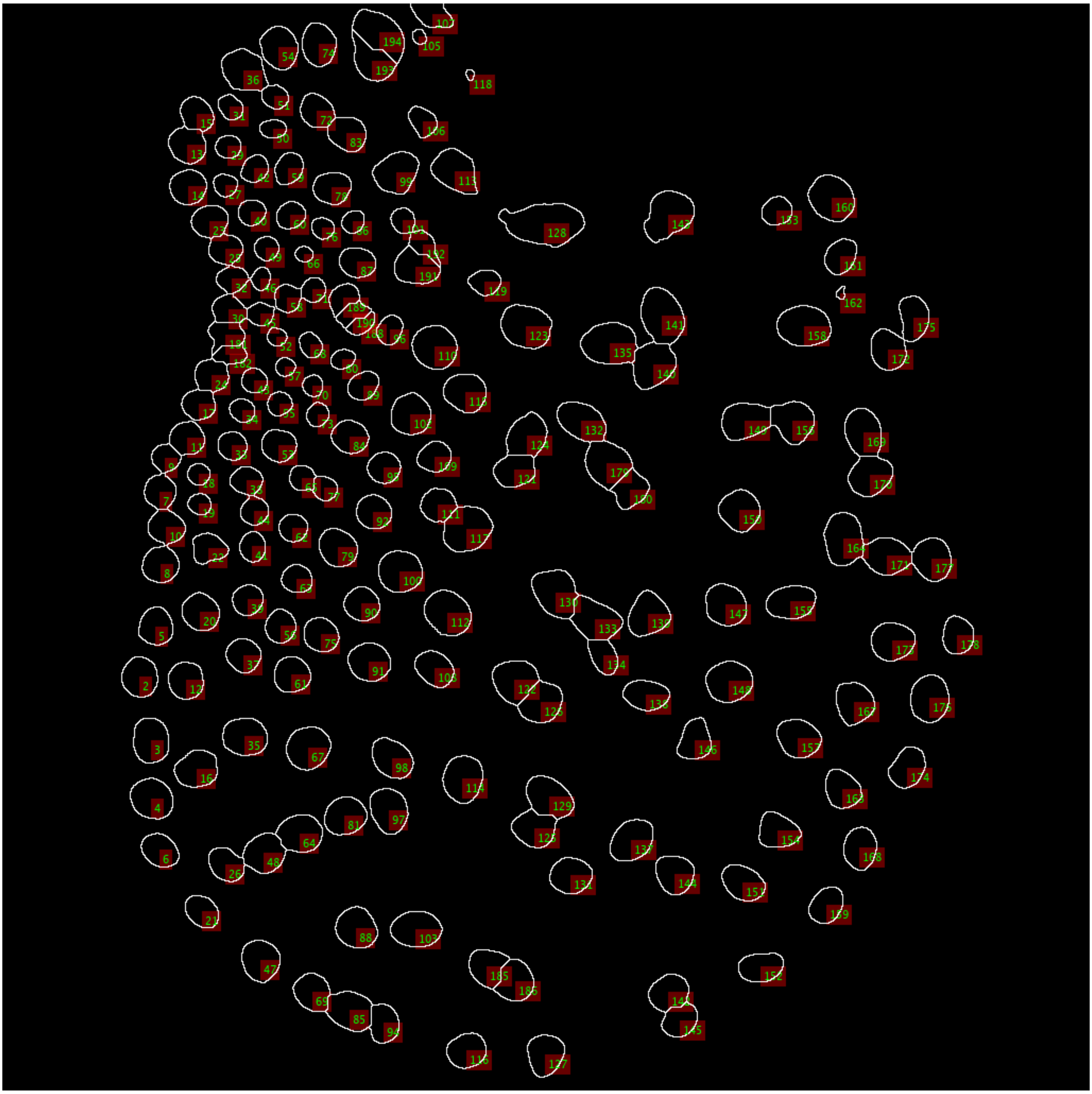

66h

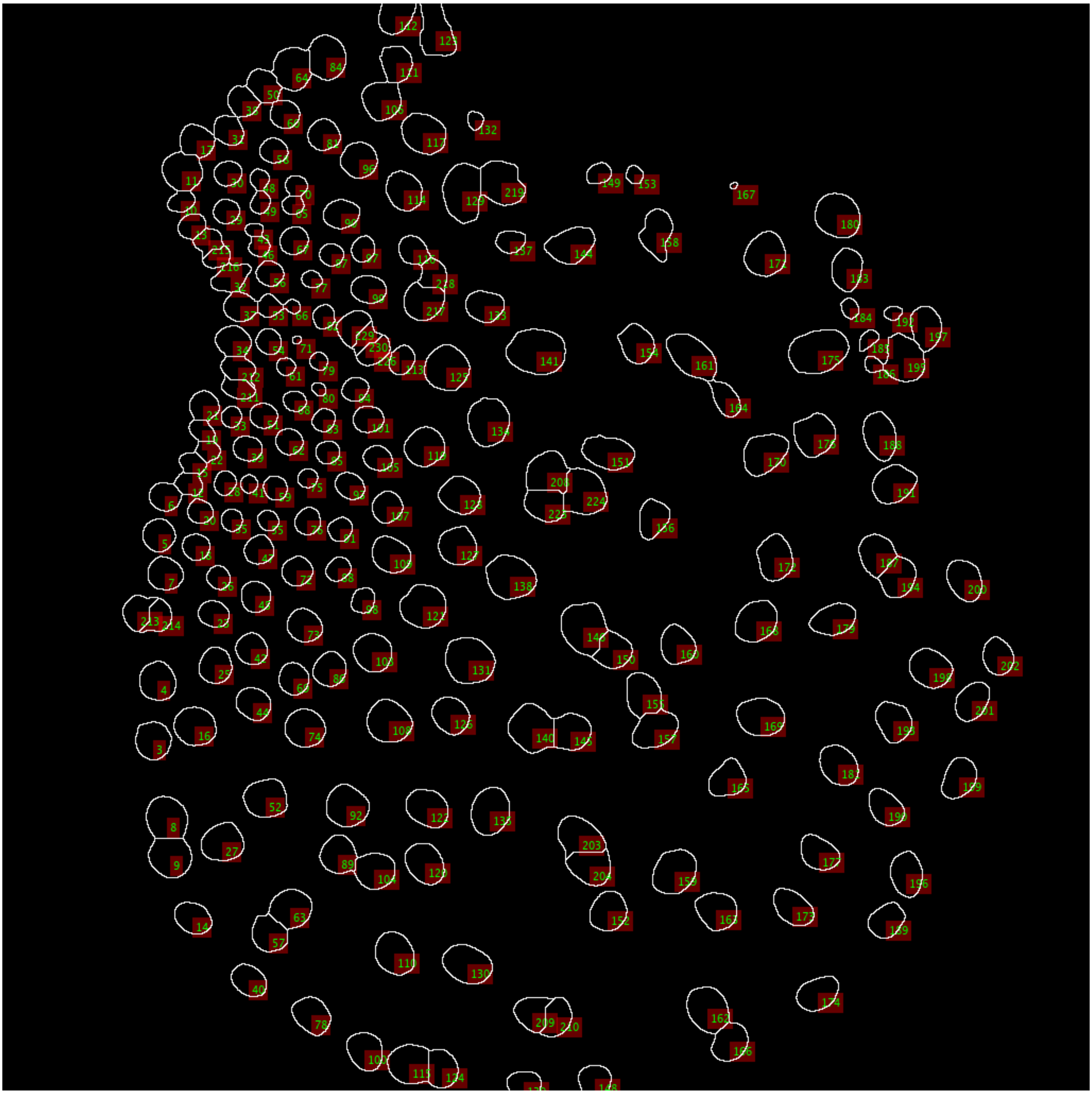

72h

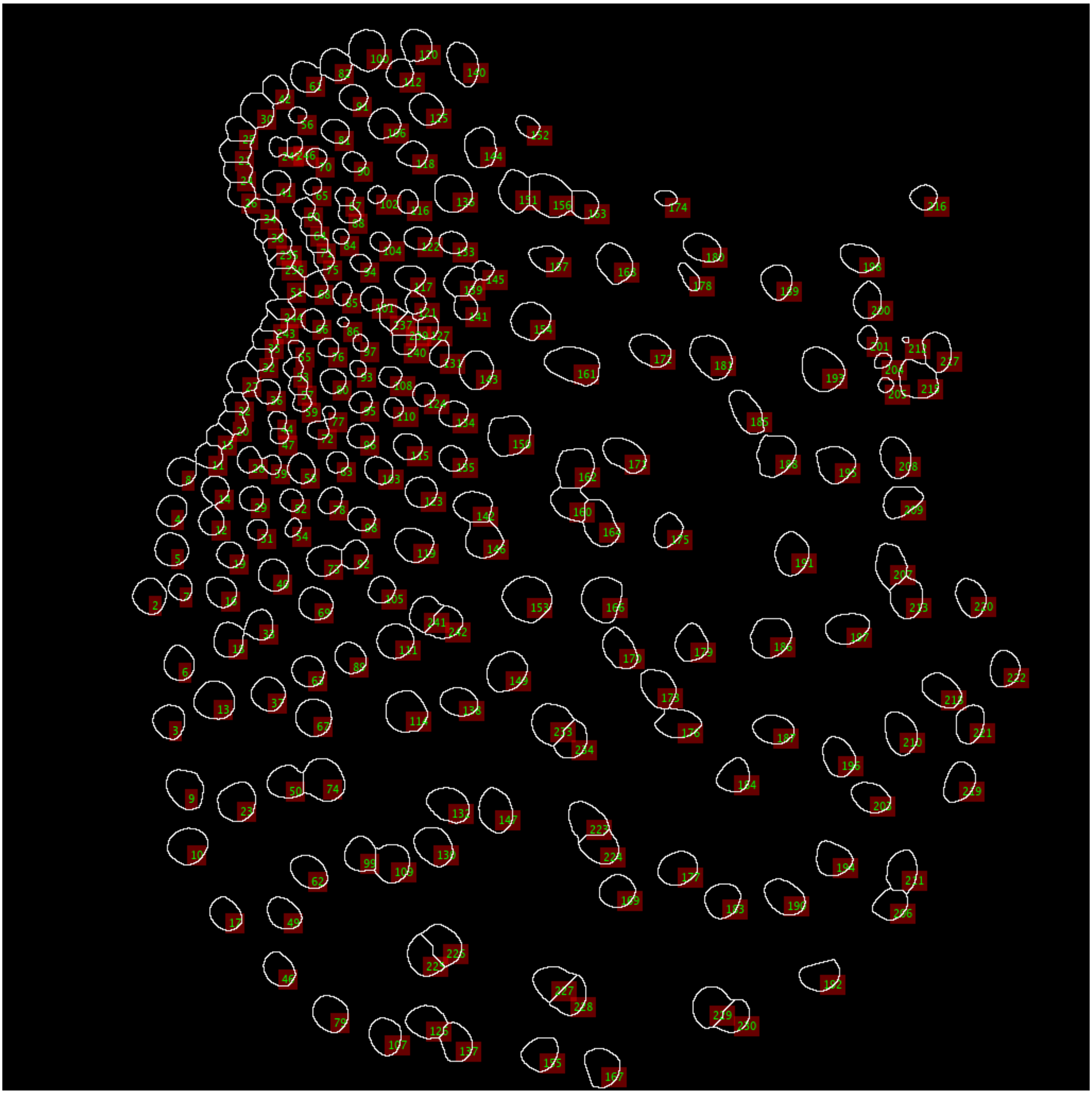

78h

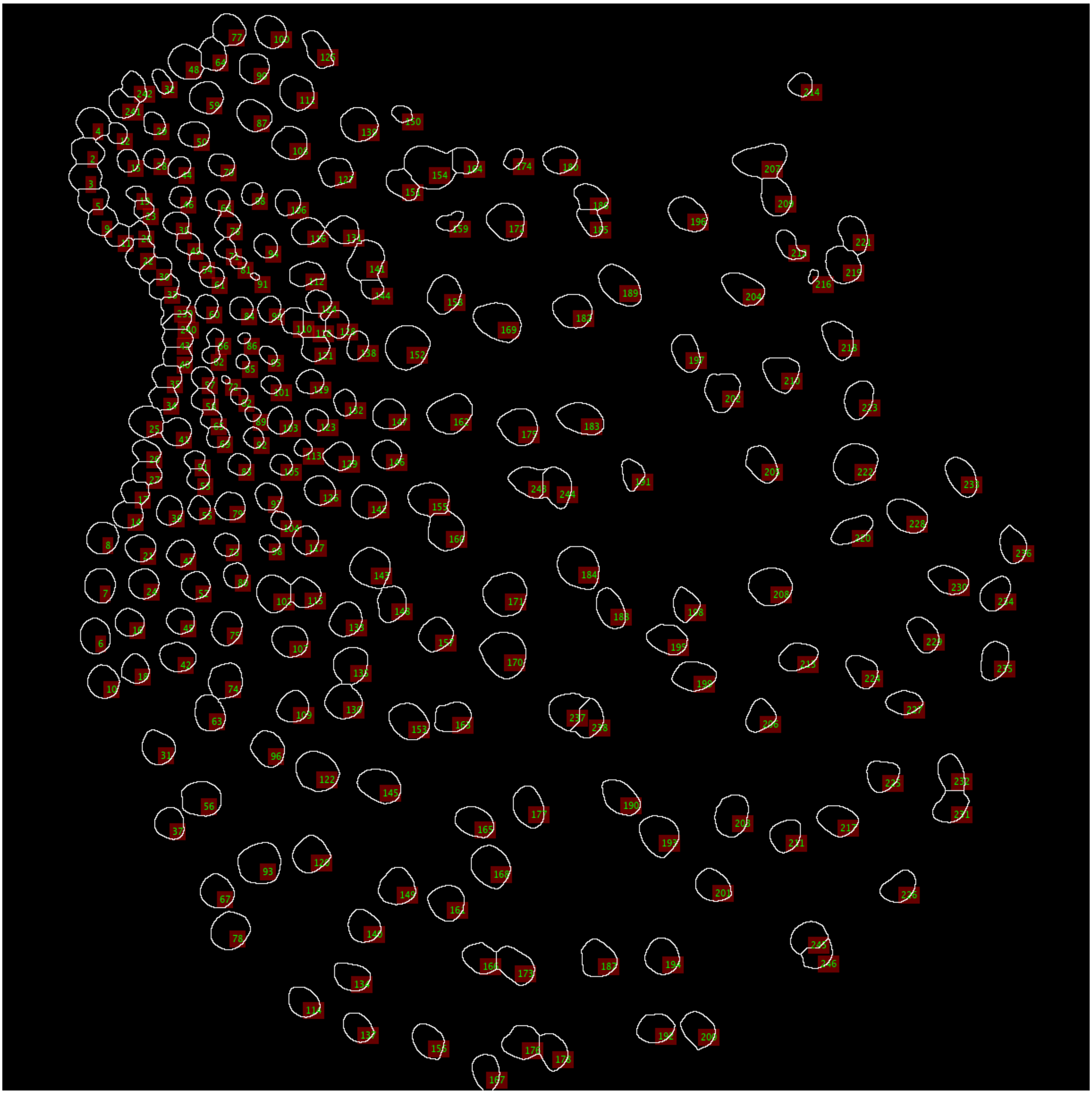

84h

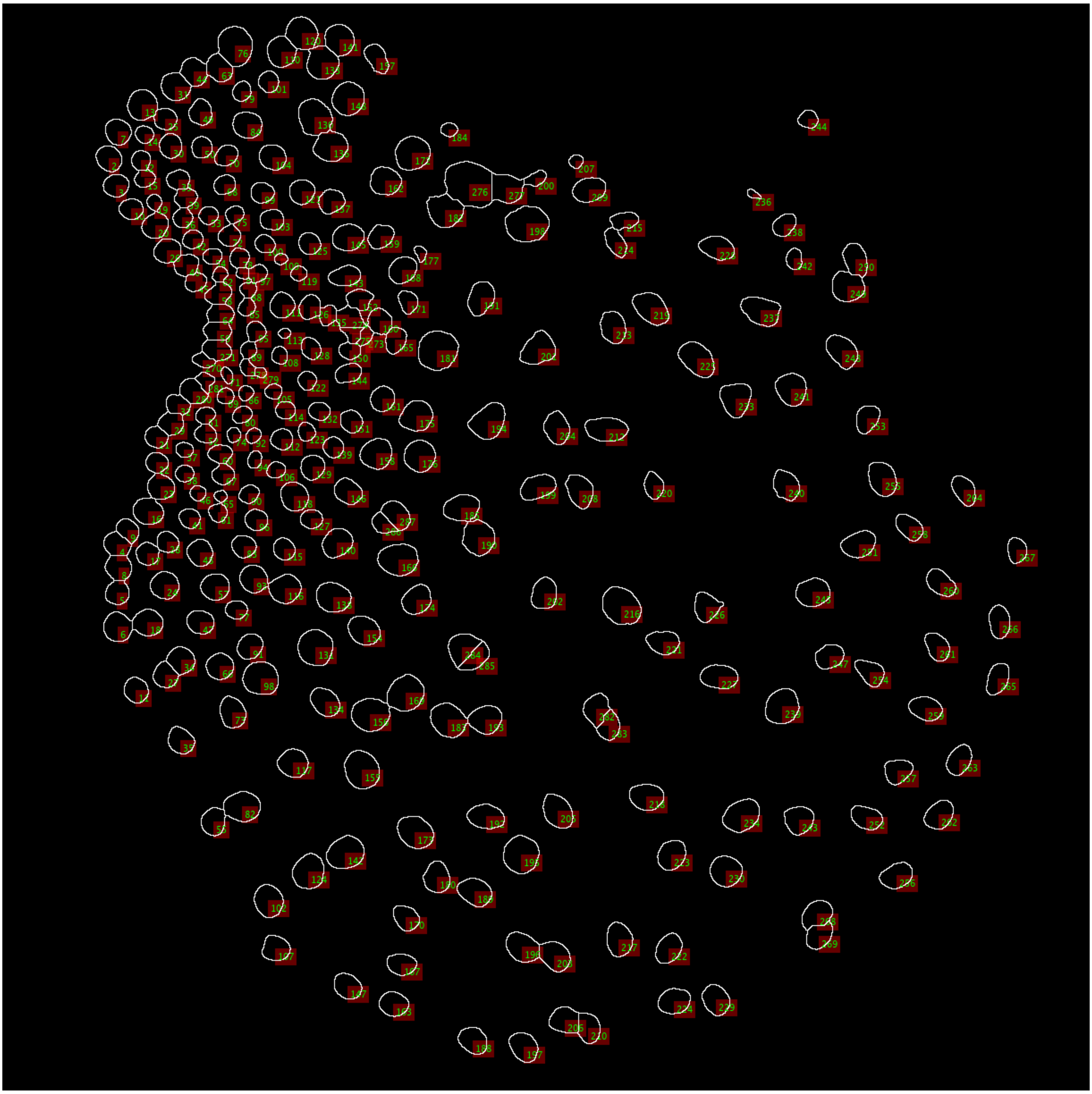

90h

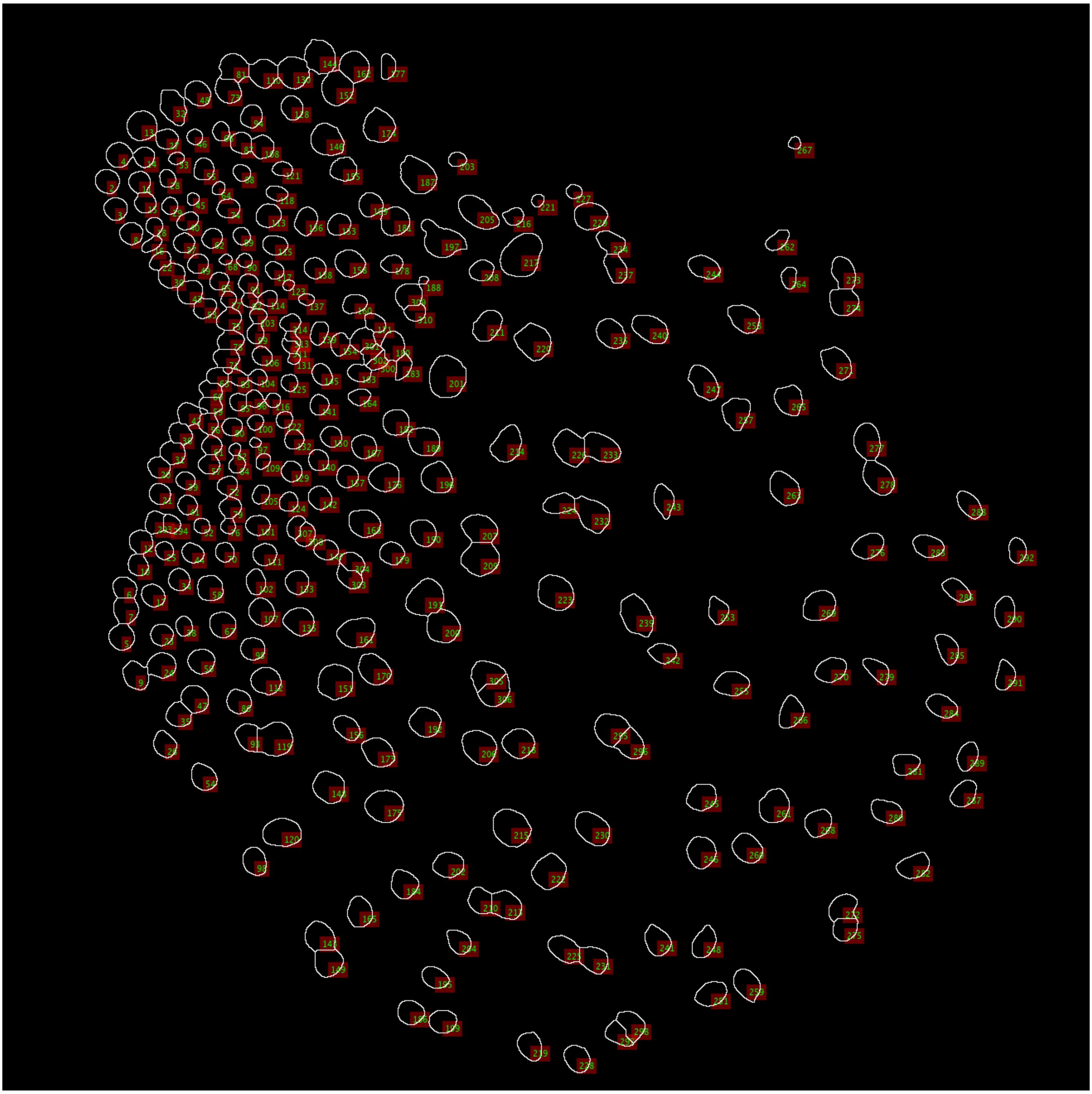

96h

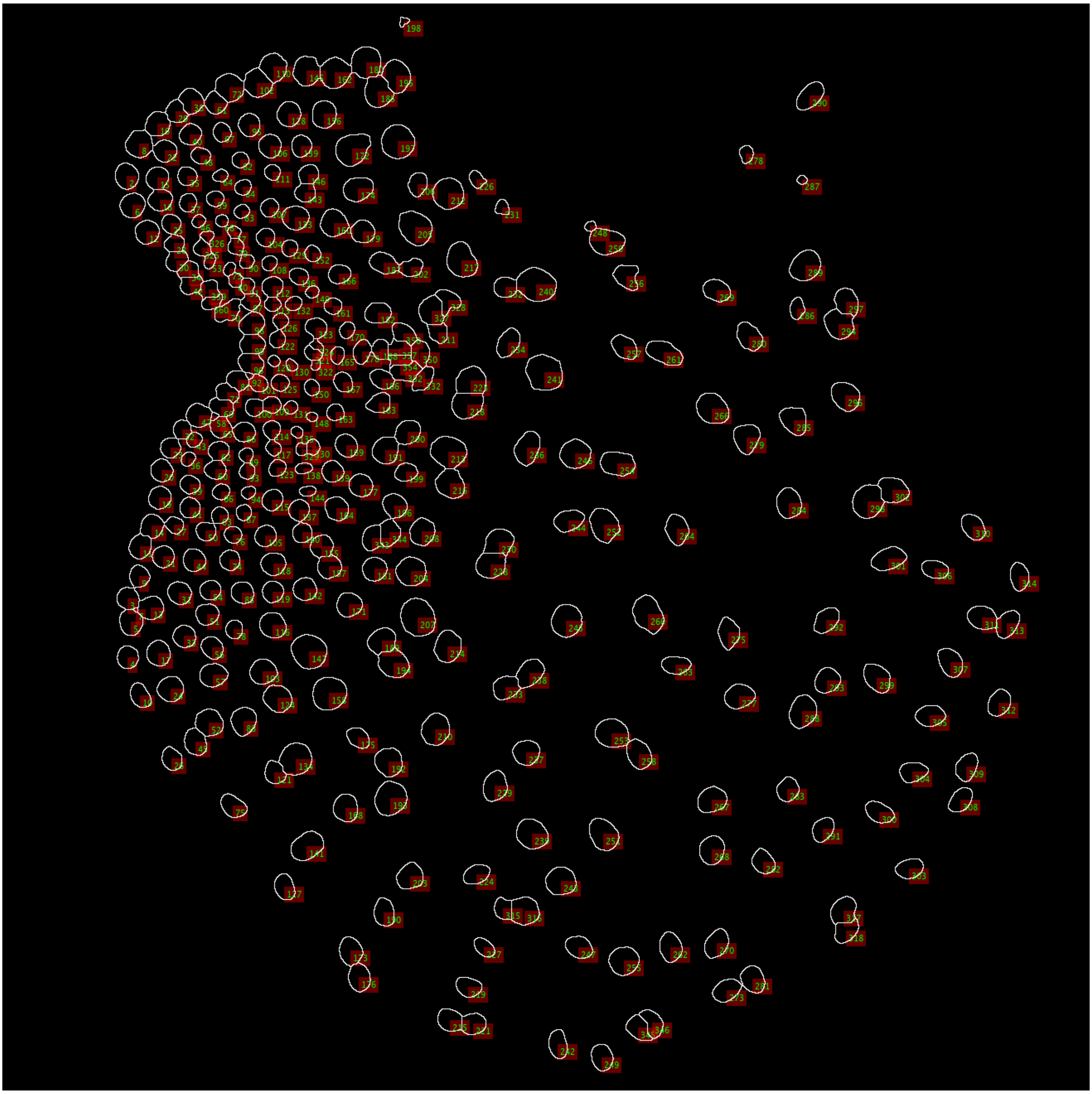

102h

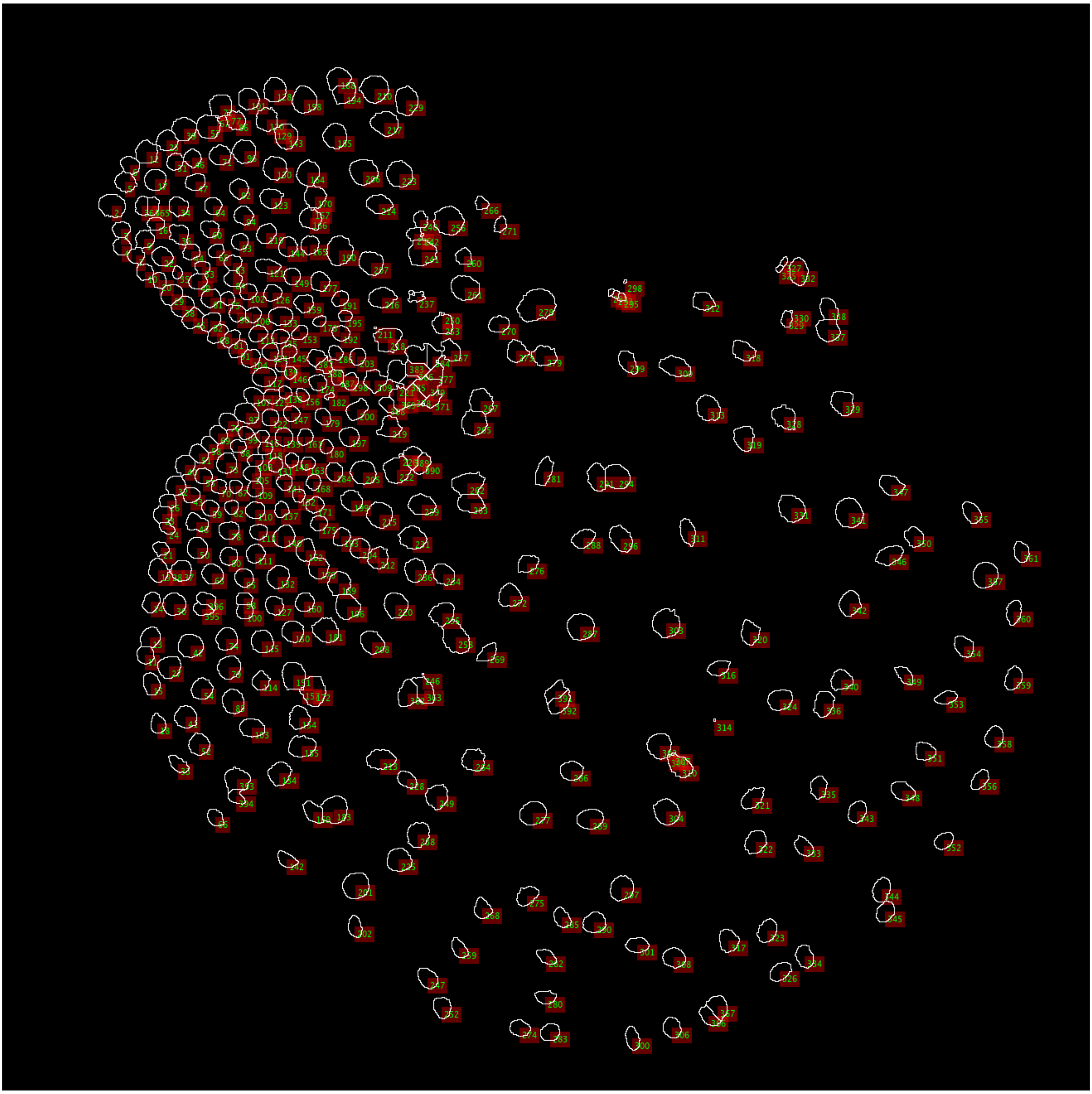

108h

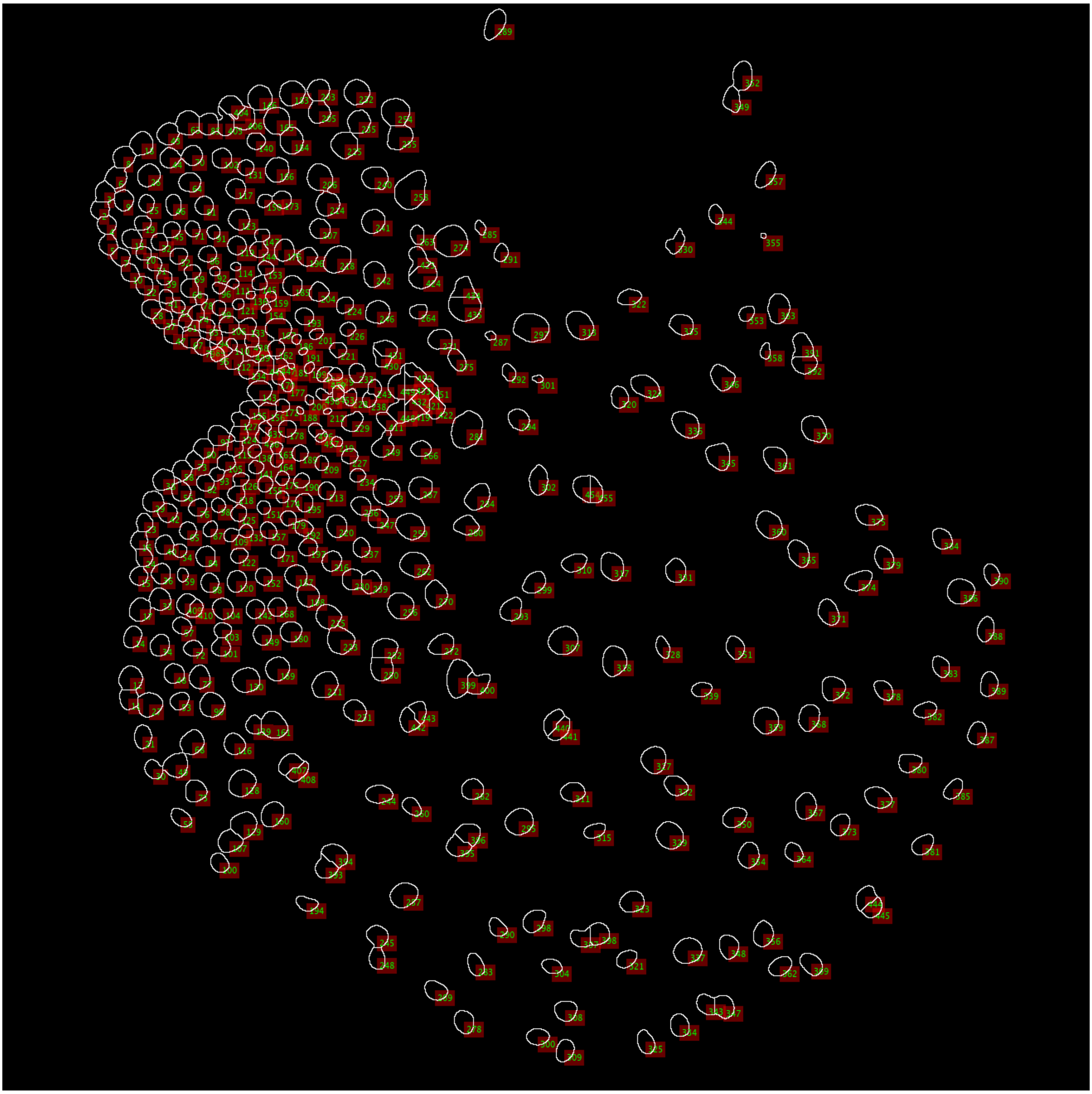

Supplement: Supplementary file 1 — Supplementary information [file 42003_2022_3627_MOESM1_ESM.pdf]
