# Supplementary material for: Positional cues and cell division dynamics drive meristem development and archegonium formation in Ceratopteris gametophytes
Source: Commun Biol. 2022 Jul 1;5:650. doi: 10.1038/s42003-022-03627-y (PMC9249879; doi:10.1038/s42003-022-03627-y)
Supplement: Supplementary file 2 — Description of Additional Supplementary Files [file 42003_2022_3627_MOESM2_ESM.pdf]

Description of Additional Supplementary Files

File name: Supplementary Data 1
Description: Data for Fig. 4. Number of cell division events for each lineage every six hours. The ID of each cell is shown in Supplementary Fig. 19

File name: Supplementary Data 2
Description: Data for Fig. 5. Number of cell division events for each progenitor cell and its descendants during the indicated time intervals. The ID of each cell is shown in Supplementary Fig. 19.

File name: Supplementary Data 3
Description: Data for Fig. 6. Number of cell division events from 30h to 108h. The ID of each cell is shown in Supplementary Fig. 19.

File name: Supplementary Data 4
Description: Data for Fig. 7b-g. Distances between archegonia and the meristem notch during gametophyte development.

File name: Supplementary Data 5
Description: Data for Supplementary Fig. 10d. Number of cell division events within the 54-108h time frame in the marginal cells and their adjacent inner cells from three gametophytes.

File name: Supplementary Movie 1
Description: 3D rotational view of the hermaphroditic gametophyte at 9 DAI shown in Fig. 1b. The Ceratopteris prothallus develops a flat sheet of cells, and H2B-GFP labels each nucleus in the prothallus. Green: H2B-GFP, Purple: PI stain.

File name: Supplementary Movie 2
Description: 3D rotational view of the hermaphroditic gametophyte at 11 DAI shown in Fig. 1c. The Ceratopteris prothallus develops a flat sheet of cells, and H2B-GFP labels each nucleus in the prothallus Green: H2B-GFP, Purple: PI stain.

File name: Supplementary Movie 3
Description: 3D rotational view of the hermaphroditic gametophyte with two developing archegonia at 13 DAI, as shown in Fig. 1g. H2B-GFP labels each nucleus of archegonia. Green: H2B-GFP, Purple: PI stain.
